# Sequence signatures extracted from proximal promoters can be used to predict distal enhancers

**DOI:** 10.1186/gb-2013-14-10-r117

**Published:** 2013-10-24

**Authors:** Leila Taher, Robin P Smith, Mee J Kim, Nadav Ahituv, Ivan Ovcharenko

**Affiliations:** 1 Computational Biology Branch, National Center for Biotechnology Information, National Library of Medicine, National Institutes of Health, Bethesda, MD, 20894, USA; 2 Institute for Biostatistics and Informatics in Medicine and Ageing Research, University of Rostock, Rostock, 18057, Germany; 3 Department of Bioengineering and Therapeutic Sciences, University of California San Francisco, San Francisco, CA, 94158, USA; 4 Institute for Human Genetics, University of California San Francisco, San Francisco, CA, 94158, USA

## Abstract

**Background:**

Gene expression is controlled by proximal promoters and distal regulatory elements such as enhancers. While the activity of some promoters can be invariant across tissues, enhancers tend to be highly tissue-specific.

**Results:**

We compiled sets of tissue-specific promoters based on gene expression profiles of 79 human tissues and cell types. Putative transcription factor binding sites within each set of sequences were used to train a support vector machine classifier capable of distinguishing tissue-specific promoters from control sequences. We obtained reliable classifiers for 92% of the tissues, with an area under the receiver operating characteristic curve between 60% (for subthalamic nucleus promoters) and 98% (for heart promoters). We next used these classifiers to identify tissue-specific enhancers, scanning distal non-coding sequences in the loci of the 200 most highly and lowly expressed genes. Thirty percent of reliable classifiers produced consistent enhancer predictions, with significantly higher densities in the loci of the most highly expressed compared to lowly expressed genes. Liver enhancer predictions were assessed *in vivo* using the hydrodynamic tail vein injection assay. Fifty-eight percent of the predictions yielded significant enhancer activity in the mouse liver, whereas a control set of five sequences was completely negative.

**Conclusions:**

We conclude that promoters of tissue-specific genes often contain unambiguous tissue-specific signatures that can be learned and used for the *de novo* prediction of enhancers.

## Background

A fundamental question in biology is how cells and tissues differentiate and maintain their identity from essentially the same genome. Wide variation in spatial, temporal and condition-dependent expression patterns of more than 20,000 genes in the human genome [1] is required for the establishment and maintenance of different cell fates and environmental responses. Tissue-specific genes are often implicated in distinct developmental and metabolic pathways and therefore may constitute good candidates for biomarkers or drug targets.

The control of gene transcription is mediated by transcription factors (TFs), which interact in a sequence-specific manner with DNA motifs, known as TF binding sites. The promoter is frequently divided into a basal core, covering approximately 100 bp upstream of the transcription start site (TSS), and a proximal promoter, which extends up to a few hundred base pairs and typically contains multiple TF binding sites [2,3]. In addition to promoters, other *cis*-regulatory sequences, such as enhancers, are specifically bound by TFs and are central players in the control of transcription in multicellular eukaryotes. The regulation of promoters by distal enhancers involves DNA looping or scanning and/or higher-order conformation changes in chromatin [4-6], resulting in an increase in the local concentration of TFs in the vicinity of a promoter and the initiation or enhancement of transcription. It has been long recognized that proximal promoters and enhancers are functionally similar, and virtually undistinguishable from each other (see, for example, [7,8]).

Both enhancers and promoters have been shown to contain DNA motifs for specific TFs, depending on their tissue-specific activities (for example, [8-10]). In particular, CpG-depleted promoters are enriched with DNA motifs [11], suggesting a distinct regulatory mechanism from CpG-rich promoters. The transcription complex LDB1, which involves GATA1, GATA2, TAL1, LMO2, and RUNX1, and has been extensively studied in the context of the differentiation of erythroid cells, illuminates this distinction. Whereas LDB1 binds mostly within CpG-depleted promoters, it only binds downstream of CpG-rich promoters, often within the first intron of their target gene [12]. Additional evidence suggests that such DNA motifs representing putative TF binding sites are predictive of promoter activity, including tissue-specific expression of their target gene (for example, [13,14]). In addition, DNA motif enrichment analyses have shown that DNA motifs are highly predictive of enhancer activity [15-18].

Unlike promoters, enhancers can act over very long distances. Based on the relative location of conserved non-coding elements (CNEs) in the human genome, early estimates suggested that a large number of enhancers are more than 250 kilobases (kb) away from their target gene [19]. For example, a conserved enhancer of *Shh* that is associated with polydactyly is located 1 megabase (Mb) upstream of *Shh*, within an intron of another gene [20]. Furthermore, similar approaches have determined that the regulatory elements controlling the transcription of SOX9 are scattered over 1 Mb upstream of its TSS [21,22]. More recently, genome-wide chromatin interaction analyses have confirmed that such long-range interactions are indeed widespread, providing evidence that the vast majority of enhancers target genes other than their nearest genes [23,24]. Because of their genomic distribution and poorly characterized sequence features, enhancers have been difficult to identify. Only the advent of high-throughput sequencing technologies has led to large-scale screens for regulatory sequences that are now starting to reveal complete regulatory networks and signal transduction pathways in higher eukaryotes [25]. Such screens, however, represent a snapshot of a single cell type and set of conditions, and conclusions cannot, therefore, be easily generalized.

Previous studies have focused on identifying sequence features in either promoters or enhancers, and constructing models that describe these genomic elements, individually. Here, we show how the presence and/or absence of motifs in the promoter regions of genes with tissue-specific expression profiles can be used to reliably identify distal enhancers with analogous tissue-specific activity. Predicted enhancers are highly enriched in the loci of concordantly expressed genes (for instance, in the case of predicted liver enhancers, they are five-fold more abundant in the loci of most highly expressed liver genes than in the loci of lowly expressed liver genes), and overlap significantly with chromatin signatures predictive of enhancer activity. Experimental validation in mice supports the high accuracy of the presented method in predicting tissue-specific enhancers. With the advent of new technologies and the resulting deluge of expression data, approaches exploiting sequence features shared between promoters and enhancers hold great promise to understanding the *cis*-regulatory code encrypted in the genomes of higher organisms.

## Results and discussion

### Promoters of tissue-specific genes contain tissue-specific signatures

Although most promoters drive basal levels of transcription ubiquitously, some promoters are capable of controlling transcription in a tissue- and/or temporal-specific manner [26-29]. Genes controlled by these types of promoters are expressed in specific tissues and developmental stages, and may be induced by endogenous or exogenous factors. Here, we set out to systematically test whether promoters of genes that exhibit a particular expression profile in a given tissue contain sequence signatures that confer tissue-specificity and separate them from ubiquitous promoters. For this purpose, we collected the promoter regions (from 25 kb upstream to 0.5 kb downstream of the TSS; see Materials and methods) of the top 200 highly expressed genes in 79 different tissues and cell types [30] (see Materials and methods). As negative controls, we selected the promoters of the 200 least expressed genes in the same set of tissues. Although expression breadth was not considered in the construction of such gene sets, most of the genes in the sets are only expressed at high (or low) levels in the tissue of interest. Even if some genes are expressed across several tissues at high or low levels, the sets are highly non-overlapping (Supplementary notes in Additional file 1). Thus, while the individual genes in a given set are not strictly tissue-specific, the set itself is.

**Figure 1:**
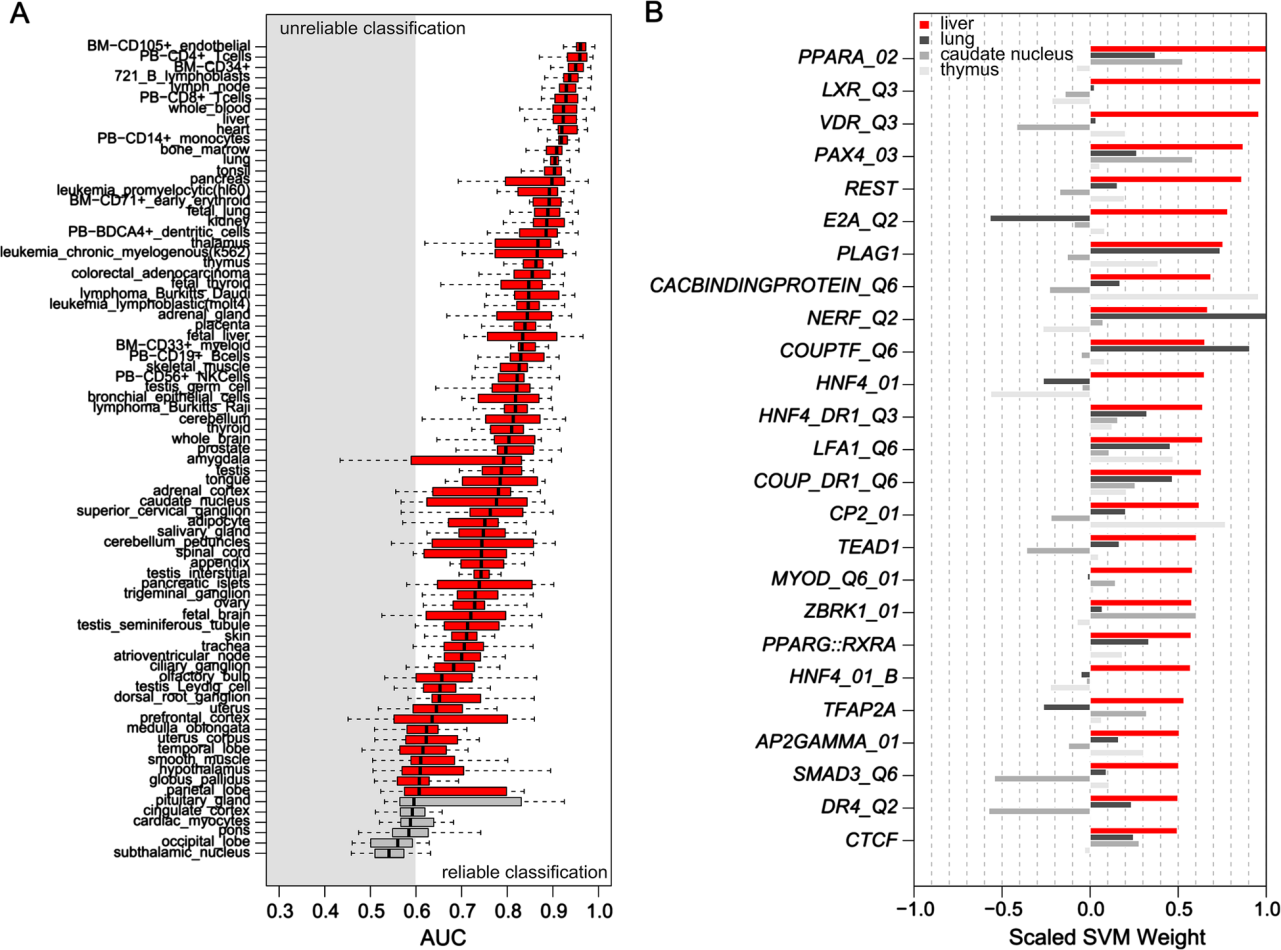
**DNA motifs in human promoters predict tissue-specific expression. (A)** Area under the receiver operating characteristic (ROC) curve for 79 models trained and tested on promoters of genes highly expressed in 79 different tissues. The AUC is an overall summary of diagnostic accuracy. AUC equals 0.5 when the ROC curve corresponds to random chance and 1.0 for perfect accuracy. Reliable models (with median AUC ≥0.6) are displayed in red, while unreliable models (with median AUC ≤0.6) are displayed in gray. Models were evaluated in a five-fold cross-validation setting. **(B)** Motifs with the greatest predictive power for the liver model. The weights w of the motifs (see Materials and methods) are given in red. Motif weights have been scaled to [-1, 1], where 1 represents the scaled weight of the motif with highest predictive power, and -1 the scaled weight of the motif with the lowest negative predictive power (signs are preserved; see Materials and methods). The names of the features are listed near the baseline of the graph. For comparison, we include weights w for the same motif in the lung, caudate nucleus, thymus models (in different shades of gray). Similarities among the genes that were used to train the models - which reflect functional relatedness among tissues - explain similarities in the predictive power of the motif. Thus, 15% of genes that are highly expressed in liver are also highly expressed in lung, while less than 5% are in caudate nucleus and thymus.

To assess the role of promoters in determining tissue-specific expression, we trained a support vector machine (SVM) for each of the 79 tissues. More precisely, to discriminate between promoters of most highly expressed and inhibited genes in each tissue, the classifiers relied on *in silico* occurrences of TF binding sites within their evolutionary conserved regions (see Materials and methods; Supplementary notes in Additional file 1). We evaluated the models’ ability to accurately predict expression using the area under the receiver operating characteristic curve (AUC) in a five-fold cross-validation framework. Most models (73/79) can reliably distinguish promoters of genes most highly expressed in a given tissue from those of lowly expressed genes by identifying TFs associated with these tissues with median AUC values between 0.60 and 1.00 (Figure 1A). To be specific, for half of the models (39/79), we obtained median AUC values higher than 0.8. Moreover, when we tested a model trained on a particular tissue on the promoters of genes expressed in another tissue, we obtained relatively high AUC values mainly for related tissues (Figure S1 in Additional file 1), confirming that the models rely on tissue-specific motifs. We even obtained high AUC values for models in which, at first glance, we could not detect any significantly enriched motifs, such as for BM-CD71+ early erythroid cells. This result suggests the existence of different subsets of promoters, with characteristic sequence features. Modest performance is likely explained by lack of sequence features and/or relatively high heterogeneity of the promoters in the training set of the model. Thus, our models performed well even in the presence of a relatively large fraction of promoters overlapping CpG islands, but yielded higher AUC values when trained on CpG-poor promoters (with the mean fraction of promoters overlapping CpG islands being 0.58 for reliable models, as compared with 0.67 for unreliable models; Figure S2 in Additional file 1; Pearson’s r^2^ = 0.1 with *P*-value = 0.001). Since genes expressed in the brain are strongly associated with CpG islands [31,32], many of the models yielding low AUC values involved brain tissues. The performance of the models is also negatively correlated with the fraction of promoters enriched in TATA-box motifs (with the mean fraction of promoters containing TATA boxes being 0.49 for reliable models, as compared with 0.57 for unreliable models; Figure S3 in Additional file 1; r^2^ = 0.4, *P*-value = 2.8 × 10^-11^). Additionally, promoters of most highly expressed genes in reliable models are less conserved at the TSS compared to those in poor models (with average percentage of sequence identity between human and mouse of 0.63 for reliable models, as compared with 0.70 for unreliable models; Figure S4 in Additional file 1; r^2^ = 0.4, *P*-value = 4.4 × 10^-10^). The genes regulated by these promoters exhibit similar conservation trends. This result suggests that extensive use of promoters with tissue-specific activity could have arisen as a means to facilitate the acquisition of novel gene functions.

We next observed that many of the most highly predictive motifs for tissue-specific gene expression (that is, those with the largest positive weights; see Materials and methods) for reliable models are known to be involved in the regulation of the corresponding tissue. For instance, motifs with the highest predictive power (among the top 2%) for the liver model included binding sites for HNF4A, PPARA, NR1H3, and NR2F2 (Additional file 2), which are among TFs that have been experimentally shown to control hepatic function and development [33-36]. This analysis is limited in that TFs may recognize similar binding sites and in that motif databases are partially redundant. Thus, establishing the identity of the TF that may be binding to particular motifs is not trivial. However, taken as a whole, these observations suggest that our model specifically captures key regulators of the liver transcriptional network (Figure 1B). Also, as expected, binding sites for the same TFs characterize models for tissues with similar gene expression profiles. For example, most brain tissues share binding sites for members of the Hox and Pax families of TFs (Additional file 3), confirming the correlation between motifs with high predictive power and tissue-specific regulation.

In summary, the strong predictive value of the motifs identified in promoter regions confirms that they are highly associated with tissue-specific gene expression, and substantiates the involvement of promoters in the regulation of tissue-specific expression.

### Promoter signatures identify tissue-specific enhancers

We next assessed whether the models describing tissue-specific promoter activity could be exploited to discover enhancers. For this purpose, we applied each of the 73 reliable models trained on promoter regions to predict enhancers in the loci of genes that were among the 200 most highly or lowly expressed in the corresponding tissue (see Materials and methods). We evaluated only evolutionarily conserved non-coding sequences across the human and mouse genomes located at least 2.5 kb upstream and 0.5 kb downstream of the nearest TSS (see Materials and methods). Recent studies suggest that only about 50% are conserved in mammals, the remainder constituting lineage-specific elements (for example, [37-41]). This fraction, however, is expected to depend on the particular tissue where the enhancers are active. On the other hand, only 10% of the genomic sequence is conserved between mammals. This makes conservation an effective filter for enhancer identification. Indeed, integrating sequence analysis with comparative genomics has been shown to reveal important subsets of enhancers (for example, [17,18,42,43]). While restricting the analysis to conserved sequences implies a reduction in sensitivity, we considered this filter essential to increase the specificity of our approach. While tissue-specific enhancers often regulate gene expression over longer distances, they tend to be enriched near genes that are expressed and functional in the tissue of interest [40,44,45]. Hence, differences in the enrichment of candidate tissue-specific enhancers between the loci of genes most highly and lowly expressed in the corresponding tissue could be used as an indicator of whether the predicted enhancers do indeed drive tissue-specific expression.

**Figure 2:**
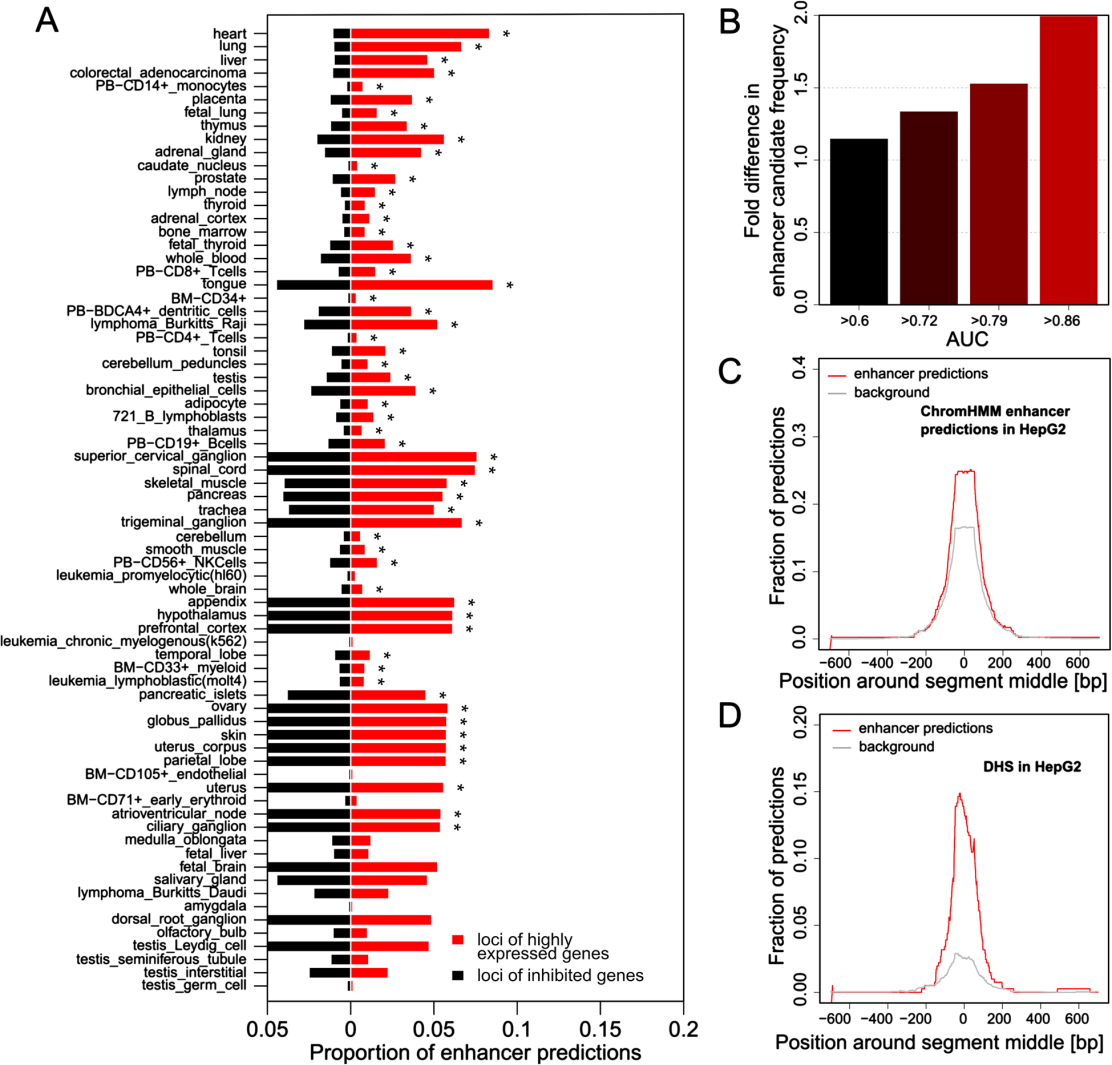
**Genome-wide enhancer predictions. (A)** The number of enhancer predictions in the loci of highly expressed genes divided by the total number of sequences scanned in the loci of highly expressed genes (in red), as compared to the number of enhancer predictions in the loci of lowly expressed genes divided by the total number of sequences scanned in the loci of lowly expressed genes (in black), for 71 promoter-based models. Statistically significant differences are indicated by asterisks (*P*-values ≤0.05, Fisher’s exact test). **(B)** Correlation between the fold enrichment between the proportions of enhancer predictions in the loci of highly expressed and lowly expressed genes with the cross-validation accuracy of the corresponding promoter-based models. **(C)** Overlap of liver enhancer predictions with strong enhancers predicted by ChromHMM in HepG2 cell lines [51], compared to random sequences with similar length in the loci of genes highly expressed in liver. **(D)** Overlap of liver enhancer predictions with DNase I hypersensitivity sites (DHS) in HepG2 cell lines from the ENCODE project, compared to random sequences with similar length in the loci of genes highly expressed in liver.

In 78% of tissues, our enhancer predictions are enriched in the loci of the 200 most highly expressed genes as compared to lowly expressed genes (*P*-values ≤0.05, Fisher’s exact test; Figure 2A; see Materials and methods for details). The most pronounced enrichment in physiologically normal tissue was observed in heart, lung, and liver, with fold differences of at least 4.5. Moreover, for 44% of the tissues, we also found predictions in a significantly larger fraction of loci of most highly expressed genes as compared to loci of lowly expressed genes. For instance, we observed candidate liver enhancers in the 60% of the loci of most highly expressed genes, but only in 43% of the loci of lowly expressed genes (*P*-value = 0.01, computed with Fisher’s exact test; Figure S5 in Additional file 1). Finally, for 26% of the tissues the scores of the candidate enhancers were significantly greater in the loci of most highly expressed genes as compared with those in the loci of lowly expressed genes (*P*-value ≤0.05, Wilcoxon rank-sum test; Figure S6 in Additional file 1), suggesting that increasing the stringency of the prediction threshold would result in even stronger associations. In total, enhancer predictions in the loci of most highly expressed genes differed significantly from their counterparts in the loci of lowly expressed genes for 85% of the tissues examined according to at least one of the above-mentioned criteria, with 14% (10) of the tissues exhibiting significant differences according to all of them (Table 1; Additional file 3).

**Table 1:** Tissues for which the promoter models produce the most robust sets of predictions for enhancers

**Tissue**	**Number of enhancer predictions**		**Fraction of loci with enhancer predictions**		**Prediction scores**	**AUC**
	**Fold enrichment**	***P*-value**	**Fold enrichment**	***P*-value**	***P*-value**	
Adrenal gland	2.70	1.39 × 10^-73^	1.13	4.92 × 10^-2^	2.64 × 10^-5^	0.83
Colorectal adenocarcinoma	4.63	1.79 × 10^-155^	1.25	2.12 × 10^-3^	4.11 × 10^-8^	0.85
Heart	7.78	1.21 × 10^-277^	1.31	2.82 × 10^-4^	2.19 × 10^-13^	0.93
Kidney	2.76	1.84 × 10^-91^	1.34	1.07 × 10^-6^	1.48 × 10^-7^	0.89
Liver	4.69	4.99 × 10^-93^	1.40	4.75 × 10^-4^	9.08 × 10^-5^	0.92
Lung	6.53	1.22 × 10^-220^	1.50	9.60 × 10^-8^	3.11 × 10^-6^	0.91
Placenta	2.99	4.18 × 10^-83^	1.28	4.13 × 10^-4^	5.76 × 10^-4^	0.83
Prefrontal cortex	1.23	2.27 × 10^-10^	1.21	3.47 × 10^-6^	2.71 × 10^-2^	0.66
Spinal cord	1.50	8.27 × 10^-38^	1.15	2.39 × 10^-4^	1.38 × 10^-2^	0.72
Tongue	1.92	3.17 × 10^-62^	1.10	3.85 × 10^-2^	4.82 × 10^-5^	0.78

Only promoter-based models yielding AUC greater than 0.6 were considered in this analysis. The performance of each model in predicting enhancers was assessed by the significance of the difference between the relative number of enhancer predictions in the loci of highly expressed and lowly expressed genes with respect to the total number of scanned sequences, the significance of the difference between the fraction of loci of highly and lowly expressed genes comprising enhancer predictions, and the significance of the difference between the scores of enhancer predictions in predictions in the loci of highly and lowly expressed genes (see Materials and methods). *P*-values were computed using Fisher’s exact test and Wilcoxon rank-sum test.

Fold enrichment between the proportions of enhancer predictions in the loci of the 200 most highly and lowly expressed genes is strongly correlated with the accuracy of the promoter models (Figure 2B). Promoter models that performed only modestly, such as those based on brain tissues (AUC ≤0.70), had limited success in predicting enhancers locus-wide (with fold enrichments reaching at most 1.3), while well-performing promoter models (AUC ≥0.90), such as those for heart, liver, kidney, and lung, achieved greater fold enrichments of at least 2.8 (for kidney). We also observed slightly higher fold enrichments between the proportions of enhancer predictions in the loci of most highly and lowly expressed genes when the difference in GC content between the former and the latter was relatively large (log2 ratio of 0.13 as compared to -0.01 for lower fold enrichments between the proportions of enhancer predictions in the loci of highly and lowly expressed genes, *P*-value = 3.2 × 10^-11^, Wilcoxon rank-sum test), suggesting a role for the GC content in the control of tissue-specific expression.

In general, enhancers predicted in the loci of the 200 most highly expressed genes in a given tissue were found to overlap extensively with experimental and computational enhancer marks characteristic of functional regulatory regions. For instance, candidate tissue-specific enhancers were found to be significantly enriched (*P*-value ≤0.05, Fisher’s exact test) in binding sites for TFs within regulatory networks that are known to be important in the respective tissues, such as MYC and NFKB1 in heart, and HNF4A and SP1 in liver [46-49] (data not shown). The combined collection of enhancers predicted in the loci of the 200 most highly expressed genes in each of the tissues considered significantly overlap with ORegAnno, a manually curated collection of regulatory sequences [50], featuring a two-fold enrichment (*P*-value <0.001, computed based on 1,000 randomized sequences genome-wide). Also, our enhancer predictions are enriched for specific epigenetic histone marks generally associated with distal transcriptional regulation, as suggested by 41% of predicted enhancers overlapping ChromHMM predictions for strong and weak enhancers (1.5-fold enrichment, *P*-value <0.001, computed based on 1,000 randomized sequences genome-wide [51]). In addition, our predictions are significantly associated with the enhancer chromatin signature H3K4me1 (1.3-fold enrichment, *P*-value ≤0.001, computed based on 1,000 randomized sequences genome-wide) and DNase I hypersensitive sites (DHSs) in different human cell lines, with a total of 42% of predicted enhancers overlapping 1% of the DHSs (1.6-fold enrichment, *P*-value <0.001, computed based on 1,000 randomized sequences genome-wide). In particular, liver enhancer predictions extensively overlap with different enhancer marks, such as p300 binding, chromatin marks, and DHSs (Figure S7 in Additional file 1). For example, 29% of liver enhancer predictions overlap chromatin marks and ChromHMM enhancer predictions for the HepG2 hepatocellular carcinoma cell line, providing additional evidence for the tissue-specificity of the activity of the predicted enhancers (Figures 2C,D; Figure S8 in Additional file 1). Substantial overlap is also observed for other classifiers with DHSs (Figure S9 in Additional file 1). Finally, we found that enhancer predictions are significantly enriched in matching p300 embryonic brain, limb, and heart enhancers (2.5-fold enrichment, *P*-value <0.001, computed based on 1,000 randomized sequences genome-wide, [45,52]).

Taken together, these observations are consistent with our promoter-based models being able to predict enhancers that drive specific expression of neighboring genes in different tissues.

### Experimental assays validate tissue-specific activity of promoter-based enhancer predictions

The most reliable evidence for the accuracy of our promoter-based models in predicting tissue-specific enhancers is the experimental verification of their regulatory activity *in vivo*. Substantiated by the consistent results from the computational analysis, we chose to validate a subset of liver enhancer predictions in the loci of highly expressed liver genes using a mouse liver reporter assay [53,54]. We selected, as described in detail below, 12 out of the total of approximately 400 regions with predicted liver enhancer activity (Table 2) and 5 regions with no predicted activity as controls (Table 3) for functional testing. Importantly, we tried to ensure that the enhancer predictions tested were not significantly different from the whole set of predictions, and chose controls exclusively based on their score. Thus, differences between enhancer predictions and controls observed for other sequence properties simply reflect an association between high scores and the existence of functional constraints, rather than bias in the selection of the sequences. Liver enhancer predictions selected for validation had an average score of 1.79, and were distributed across the complete range of scores (Figure S10A in Additional file 1). Additionally, liver enhancer predictions selected for validation are located at an average distance to the nearest TSS of 7.3 kb, and are not significantly different from the entire set of liver enhancer predictions (Figure S10B in Additional file 1). Also, liver predictions selected for validation did not exhibit statistically significant differences in the level of evolutionary constraint compared to the entire set of liver predictions, with an average phastCons score [55,56] of 0.38 (Figure S10C in Additional file 1). Finally, while half of the regions with predicted liver enhancer activity were selected randomly, the remaining half was selected randomly among those predictions overlapping with strong enhancer predictions by ChromHMM in HepG2 cell lines [51]. Controls were selected randomly among sequences that had scores in the bottom half of the score distribution for the full set of scanned sequences, had an average score of -1.70, were located 27.5 kb away from the nearest TSS, and had an average phastCons score of 0.37. Each liver enhancer prediction and control was cloned upstream of a minimal promoter element and the luciferase reporter gene (pGL4.23; Promega). Each construct was then injected using the hydrodynamic tail vein injection assay into at least three different mice, and liver enhancer activity was assayed after 24 h by measuring luciferase levels (see Materials and methods).

**Table 2:** ***In vivo *** **assay of 12 liver enhancer predictions in mouse**

**ID**	**Coordinates [hg18]**	**Score**	**Location**	**Activity**	**Chromatin state**^**a**^
E1	**Chr16:30009197-30009301**	**3.07**	**Intronic ( *TBX6 *)**	**Yes**	**No**
E7	Chr10:82023332-82023434	2.60	Intergenic (3' UTR of *MAT1A*)	No	Yes
E2	Chr17:69962796-69962894	2.21	Intergenic (4.5 kb downstream of *GPRC5C*)	No	No
E8	**Chr1:31679030-31679149**	**1.94**	**Intronic ( *SERINC2 *)**	**Yes**	**Yes**
E12	**Chr3:134934911-134935091**	**1.81**	**Intronic (TF)**	**Yes**	**Yes**
E4	**Chr11:72138832-72139119**	**1.83**	**Intronic (ARAP1)**	**Yes**	**No**
E5	**Chr17:69957921-69958023**	**1.30**	**Intergenic (3′ UTR of *GPRC5C*)**	**Yes**	**No**
E10	**Chr17:69951076-69951329**	**1.57**	**Intronic ( *GPRC5C *)**	**Yes**	**Yes**
E3	Chr11:72162942-72163179	1.47	Intronic (*STARD10*)	No	No
E9	Chr11:72168912-72169046	1.33	Intronic (*STARD10*)	No	Yes
E11	**Chr11:72166225-72166509**	**1.36**	**Intronic ( *STARD10 *)**	**Yes**	**Yes**
E6	Chr17:17439720-17439913	1.04	Intergenic (4 kb upstream of *PEMT*)	No	No

^a^Overlaps with 'strong enhancer' Chromatin State Segmentation by HMM from Broad Institute, MIT, and MGH in HepG2 cell lines. Enhancer predictions for which we observed *in vivo* activity in mouse liver are highlighted in bold.

**Table 3:** ***In vivo *** **assay of regions with no predicted regulatory activity (controls)**

**ID**	**Coordinates [hg18]**	**Score**	**Location**	**Activity**
C1	Chr15:56263227-56263340	-2.22	Intronic (*AQP9*)	No
C2	Chr6:26030369-26030485	-2.03	Intronic (*SLC17A2*)	No
C3	Chr22:19494975-19495083	-1.26	Intronic (*PI4KA*)	No
C4	Chr5:138482622-138482789	-1.67	Intronic (*SIL1*)	No
C5	Chr9:96485498-96485627	-1.32	Intergenic (50 kbp upstream of *FBP1* and C9orf3)	No

**Figure 3:**
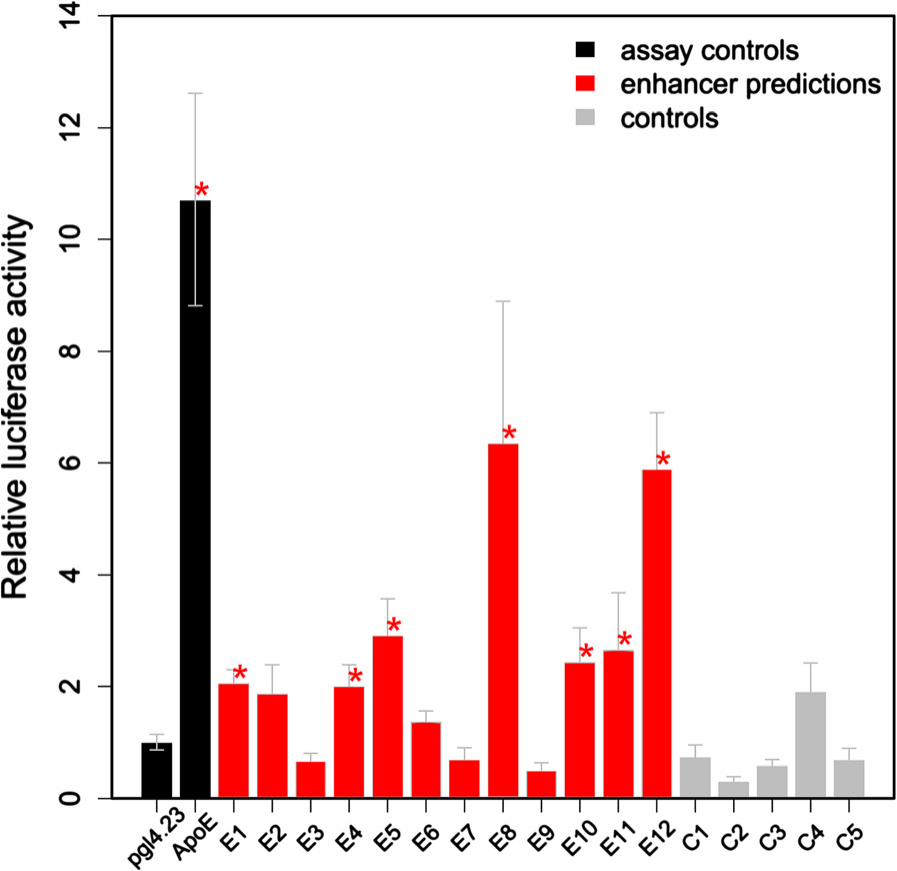
**Experimental validation of liver enhancer predictions using the hydrodynamic tail vein enhancer assay.** On each injection day, we also injected an empty pGL4.23[luc2] vector and a known liver enhancer of the *ApoE* gene as negative and positive controls, respectively. At least three mice were injected per construct. Statistical significance was tested using Student’s *t*-test followed by multiple testing adjustment with Benjamini-Hochberg’s method. The asterisks indicate statistical significance to control at adjusted *P*-value ≤0.05.

We observed statistically significant enhancer activity for 7/12 (58%) enhancer predictions compared to empty-vector-injected mice, with no significant difference depending on how predictions were selected (two-tailed Fisher’s exact test). The significant increase in luciferase activity driven by liver enhancer predictions ranged from 2.0- to 6.4-fold relative to the empty vector. By comparison, 0/5 of the controls activated the luciferase reporter (false discovery rate adjusted q < 0.05; Figure 3, Tables 1 and 2; Additional file 4). These data confirm that a large fraction of our liver enhancer predictions function as enhancers *in vivo*, regulating expression in the liver.

### Promoter-based models have the potential of shedding light on the human regulatory landscape

We applied each of the 73 reliable models trained on promoter regions to the entire sequence of the human genome. We scanned approximately 1,200,000 non-promoter CNEs across the human and mouse genomes for enhancer signatures (see Materials and methods). No model generated more than 160,000 enhancer predictions, with an average of approximately 51,000. We observed substantial overlap among enhancer predictions for related tissues (Figure S11 in Additional file 1), in part reflecting the resemblance between promoter-based models of tissues with similar gene expression profiles, but also indicating the existence of shared regulatory pathways. Thus, from all sequences scanned, approximately 900,000 (73%) were considered enhancer predictions for at least one of the models, with an average of approximately 12,000 non-redundant enhancer predictions per tissue, consistent with current ChIP-seq findings [29]. Although we estimate the false positive rate at approximately 5% based on the number of enhancer predictions in the loci of lowly expressed genes, a caveat of our approach is that local differences in the composition of the human genome could result in overall higher false positive rates. Also, consistent with the literature (for example, [57]), we found that most loci in the genome contain more than one enhancer. Indeed, without considering redundancy among predictions, we predict an average of four enhancers per locus per model, with the exact number depending on the tissue (Figure S12 in Additional file 1).

We then analyzed the distribution of enhancer predictions across the genome relative to genes. From all sequences that were classified as enhancer predictions by at least one of the models, 55% mapped within intronic regions, 43% mapped within intergenic regions, and the remaining 2% to UTRs. The trend is consistent for all tissues, in that the proportion of intronic enhancer predictions is always greater than that of intergenic predictions. Overall, tissue-specific enhancer predictions tend to be located closer to TSSs, and in particular, near TSSs of highly expressed genes in matching tissues. For example, there was more than 3-fold enrichment in liver enhancer predictions within 100 kb of the TSS of the 200 most highly expressed genes in the liver (*P*-value <0.001, computed based on 1,000 randomized sequences genome-wide), a number that increased to 4-fold enrichment within 10 kb of the TSS (*P*-value <0.001, computed based on 1,000 randomized sequences genome-wide). Furthermore, stronger enhancer predictions are closer to TSSs than weaker predictions, with, for instance, the strongest 1% of liver enhancer predictions being located 40 kb away from the nearest TSS as compared to 73 kb for the complete set of liver enhancer predictions. These results are in agreement with the literature, and suggest that the functional relevance of a genomic region depends on its position relative to the TSS [58]. Our enhancer predictions are enriched near genes annotated with relevant gene ontology terms. For example, we found more than five-fold enrichment in liver enhancer predictions within the loci of genes associated with ‘positive regulation of hepatic stellate cell activation’, ‘liver development’, and ‘positive regulation of hepatocyte differentiation’ (*P*-values <0.05, Fisher’s exact test), as well as enrichment for genes with critical liver functions, such as ‘positive regulation of cholesterol metabolic process’ (*P*-value = 2.7 × 10^-14^, Fisher’s exact test), ‘triglyceride lipase activity’ (*P*-value = 6.0 × 10^-8^, Fisher’s exact test), and sucrose, maltose, and trehalose metabolic processes (all *P*-values <0.05, Fisher’s exact test).

Although all our tissue-specific enhancer predictions were selected from conserved non-coding sequences across the human and mouse genomes, they exhibit different levels of conservation according to their phastCons scores (Figure S13 in Additional file 1). For example, liver and heart enhancer predictions in the loci of highly expressed genes are significantly more conserved than the sequences used as basis for making predictions (0.41 versus 0.34, and 0.43 versus 0.37, with *P*-values 1.1 × 10^-32^ and 5.6 × 10^-33^, respectively, calculated using the Wilcoxon rank-sum test). For models that did not perform well in terms of their fold enrichment between the proportion of enhancer predictions in the loci of highly and lowly expressed genes (for example, skin and fetal brain), we observed significantly less constrained predictions. We observed similar trends when we applied our promoter-based classifiers to investigate unconstrained sequences (see Supplementary notes in Additional file 1).

In summary, our results indicate the existence of largely disjoint sets of tissue-specific regulatory sequences located in the neighborhood of their potential target genes. They also confirm an important role for evolutionarily constrained sequences, in that 73% of sequences conserved across mammals exhibit regulatory potential. Finally, consistent with previous studies, they support a role for both promoters and enhancers in determining spatiotemporal patterns of gene expression.

## Conclusions

By analyzing the sequence of promoters of tissue-specific genes, we confirmed that tissue-specific promoters and enhancers share TF binding motifs within the loci of their cognate genes. Moreover, we observed that regulatory information in the promoters of tissue-specific genes is predictive of the enhancers targeting these genes. For 73/79 tissues, we could reliably distinguish between highly and lowly expressed genes based exclusively on the presence or absence of putative motifs (AUC ≥60%). Although similar cut-offs have been recently employed (for example, [18,59]), we recognize that the half of the models exhibiting modest performances (AUC ≤80%) might have limited predictive value. It is, however, important to note that the reported AUCs represent the lower bound of the classifier accuracy due to the fact that the strength of the tissue-specificity enhancer signal is expected to vary among the promoters of tissue-specific genes. Promoters containing only weak signals will inherently deflate the classification AUC estimates. To further address the performance of the classifiers at predicting tissue-specific enhancers, we introduced a panel of independent computational and experimental tests, which ultimately validated our analysis. Many of the TFs binding to the motifs that are identified as relevant to each of these models are known to play a fundamental role in the development or maintenance of normal function of the corresponding tissues. We showed that the motifs found in promoter regions can be used to predict enhancers with matching tissue-specificity. The accuracy of our tissue-specific enhancer predictions by promoter-based models is supported by a highly significant association of enhancer predictions with the genes most highly expressed in a given tissue, and by a significant overlap of predictions with experimentally identified tissue-specific enhancers.

More importantly, 58% (7/12) of liver enhancer predictions generated by the promoter-based model drove luciferase expression in the liver following hydrodynamic tail vein injection in mice, whereas none of the five negative controls did.

Six of the seven validated liver enhancers were located within introns (for the genes *TBX6*, *SERINC2*, *TF*, *ARAP1*, *STARD10*, and *GPRC5C*), while the remaining prediction was in the immediate vicinity of *GPRC5C*. These genes have been previously reported as moderately to highly expressed in liver and gallbladder [60]. For example, although the specific function of *GPRC5C* is unknown, the gene is highly expressed in the liver and has been suggested to play a role in signaling events when induced by retinoic acid [61]. In addition to other motifs, liver enhancer predictions that exhibited luciferase activity contained predicted binding sites for 5 to 11 out of 27 known liver TFs (Additional file 5). Although each of the critical liver TFs PPARA, PPARG, NR2F2, and HNF4A had binding sites in 6/7 (86%) sequences, no single TF had a binding site in all 7 sequences that exhibited luciferase activity, highlighting the ability of our method to model flexible regulatory sequence encryptions. In turn, each of the 7 sequences included binding sites for at least 2 of these 4 TFs, and 4/7 (57%) for all 4. The 5 liver enhancer predictions that exhibited no significant luciferase activity contained binding sites for 4 to 8 out of 27 known liver TFs. HNF4A had binding sites in all 5 sequences, PPARA and NR2F2 had binding sites in 4/5 (80%) sequences, and PPARG in 3/5 (60%). With one exception, each of the sequences included binding sites for at least 2 of these 4 TFs, and 3/5 (60%) for all 4. For comparison, 87% of all sequences scored by the liver promoter-based model contained 1 to 18 out of 27 known liver TFs. HNF4A had binding sites in 28% of the sequences. Only 11% of the sequences had binding sites for at least two TFs among PPARA, PPARG, NR2F2, and HNF4A, and 7% for all four TFs. Despite limitations in the accuracy of the TF binding site predictions and despite the fact that many motifs may be nonfunctional [62], our results suggest that particular combinations of TFs, rather than single TFs, are necessary to establish liver transcription. In addition, the function of assayed sequences may be subject to activation or inhibition by additional *cis*- and *trans*-regulatory elements. For example, enhancer activity might be induced by hormones or drugs under particular conditions [63] or depend on neighboring functional elements that are absent in the construct used for the experiment. This and other phenomena could produce false-negative observations in the reporter assays. In any case, the experimental data presented here provide independent and robust validation of the enhancer predictions obtained with the promoter-based models, and lend further support for the hypothesis that the specificity of interactions between enhancers and promoters is at least partly due to the binding of tissue-specific TFs.

Our models predict multiple tissue-specific enhancers per locus and per tissue, as well as multiple tissues or domains of activity for most enhancers. This redundancy, which has long been reported (for example, [64-66]), may serve to increase the robustness of the regulatory network [67]. Furthermore, it is likely that apparently redundant enhancers activate gene expression in different cell types and/or under different developmental stages or conditions [68-71]. The genomic distribution of enhancers is also likely to vary depending on the function of their target genes. For example, the loci of transcription factors and developmental genes are known to contain particularly high densities of CNEs, many of which act as distal enhancers [72-77]. More recently, advances in technical approaches, such as chromosome conformation capture and its derivatives, have confirmed these findings independent of sequence conservation [24,78]. We observed that relatively long loci, such as those of genes expressed in brain tissues, featured more enhancer predictions per locus compared to short loci. However, some compact loci, such as those of genes highly expressed in liver, lung, and heart, contained a relatively large number of enhancer predictions, providing evidence for a particular need for fine-tuning the expression level in these tissues. Furthermore, the level of conservation of enhancer sequences is likely to depend, as other studies suggests (for example, [79,80]), on their particularly activity, although we found that, for all models, a large proportion of the enhancer predictions is likely to be conserved across mammals.

Finally, our results add further evidence for a significant role of both promoters and enhancers in determining tissue specificity. This role is supported by several examples from the literature [14,81,82]. Different enhancer-promoter preferences would provide an additional level of transcriptional control, assisting in establishing the favorable interactions, for instance, between enhancers and their cognate promoters when they are distant, or between enhancers and their cognate promoters within a gene cluster. The intimate coordination of promoters and enhancers in regulating tissue-specific transcription has immediate practical consequences. It makes it possible to describe the complex regulatory landscape of higher eukaryotes, and eventually identify regulatory elements located hundreds of kilobases away from their target gene, based solely on the analysis of proximal regulatory elements. DNA microarrays and, more recently, RNA-seq are currently being used to profile the transcriptomes of a diverse range of cell/tissue types, conditions, and species. As more expression data become available, particularly in the context of large projects such as ENCODE [83] and the 1000 Genome Project [84], it is our belief that the application of approaches such as the one we are proposing here will result in important new insights and improve our understanding of transcriptional regulation. Such projects are also generating a wealth of epigenetics information that can be easily integrated with our models to reveal genomic signatures controlling transcription.

## Materials and methods

### Gene annotation and expression data

GNF Novartis Gene Expression Atlas version 2 [30] was extracted from the gnfAtlas2 table and mapped to the RefSeq [85] genes using the knownToGnfAtlas2 and kgXref tables (all tables are available in the UCSC Genome Browser database [86]). Thereby, we obtained expression profiles in 79 tissues (721 B lymphoblasts, BM-CD105+ endothelial, BM-CD33+ myeloid, BM-CD34+, BM-CD71+ early erythroid, PB-BDCA4+ dentritic cells, PB-CD14+ monocytes, PB-CD19+ B cells, PB-CD4+ T cells, PB-CD56+ natural killer (NK) cells, PB-CD8+ T cells, adipocyte, adrenal cortex, adrenal gland, amygdala, appendix, atrioventricular node, bone marrow, bronchial epithelial cells, cardiac myocytes, caudate nucleus, cerebellum, cerebellum peduncles, ciliary ganglion, cingulate cortex, colorectal adenocarcinoma, dorsal root ganglion, fetal brain, fetal liver, fetal lung, fetal thyroid, globus pallidus, heart, hypothalamus, kidney, leukemia chronic myelogenous (k562), leukemia lymphoblastic (molt4), leukemia promyelocytic (hl60), liver, lung, lymph node, lymphoma Burkitts Daudi, lymphoma Burkitts Raji, medulla oblongata, occipital lobe, olfactory bulb, ovary, pancreas, pancreatic islets, parietal lobe, pituitary gland, placenta, pons, prefrontal cortex, prostate, salivary gland, skeletal muscle, skin, smooth muscle, spinal cord, subthalamic nucleus, superior cervical ganglion, temporal lobe, testis Leydig cell, testis, testis germ cell, testis interstitial, testis seminiferous tubule, thalamus, thymus, thyroid, tongue, tonsil, trachea, trigeminal ganglion, uterus, uterus corpus, whole blood, whole brain) for 13,977 human genes. Overall, 5,023 genes were considered 'most highly expressed' in at least one of the 79 tissues. Additionally, 6,531 genes were least expressed in these tissues.

### Locus definition

In order to define gene loci, we first clustered together all overlapping transcripts in the refGene.txt and knownGene.txt tables (available in the UCSC Genome Browser database [86]), and then assigned the closest half of the intergenic sequence separating two genes to each of the corresponding gene loci. Although the genes that are closest to the enhancers are reasonable target genes, there are many known cases of enhancers located in introns of genes that are not their targets, as well as enhancers several kilobases away from their targets, with unrelated genes in between. Current integrative approaches result only in modest improvement in enhancer-target gene associations (for example, [87]), often requiring non-available data. Recently, a method based on Hi-C has been introduced to identify genome-wide functional domains based on higher-order chromatin interactions [5]. However, comparisons between alternative methods are limited because of the lack of an appropriate reference or gold standard.

### Promoter annotation and definition for promoter modeling

Promoter regions were defined as encompassing a 3 kb region (2.5 kb upstream and 0.5 kb downstream of the TSS), relative to 5′ TSSs of all transcripts annotated in RefSeq [85]. Although the total length is arbitrary, it intends to span both the core and proximal promoter regions. In most cases, the signal that turned out to be relevant for the models was detected within 500 bp of the TSS (Figure S14 in Additional file 1).

Gene expression values for each of the promoters of the most highly and least expressed genes in each of the 79 tissues considered were extracted from [88]. Probe IDs were converted to UCSC Known Gene IDs using [89]. Subsequently, UCSC Known Gene IDs were converted to gene symbols and RefSeq IDs using [90]. Expression values for transcripts with the same gene symbol were averaged together. The 200 most highly and least expressed genes with different gene symbols were selected. TSSs of all RefSeq IDs associated with those gene symbols were then used to define 3 kb promoter regions.

### Sequence conservation of promoter regions

Sequence identity of promoter regions was determined based on genome-genome alignment of human and mouse (from the net/chain track at UCSC [86]), using the hg18 and mm9 genome assembly, respectively.

### Sequence conservation of coding regions

As an indicator of coding conservation across species we used the proportion of orthologs of human genes found in other eukaryotic species (HomoloGene Build 64 [91]).

### Motif occurrences

Presence or absence of putative motifs was determined scanning the sequence for 775 motifs in TRANSFAC [92] and JASPAR [93-95] using MAST [96] with default parameters.

#### Motif over-representation in promoter regions

Over-representation of 775 motifs representing TF binding sites in TRANSFAC and JASPAR among promoter regions of the 200 most highly expressed genes in each of the 79 tissues considered was determined by comparing the promoter regions of the 200 most highly expressed genes to the promoters of the 200 least expressed genes in the corresponding tissue. The entire length of the promoter region (-2.5 kb to +0.5 kb with respect to the TSS) was searched for motif occurrences with MAST. The numbers of putative TF binding site occurrences in each set of promoters were compared using the Wilcoxon rank-sum test.

### Transcription factors associated with transcription factor binding sites

TF annotation for position-weight matrices (PWMs) was obtained from TRANSFAC [92], JASPAR [93-95], and the Broad Institute (MSigDB [97]).

### CpG islands and TATA-box motifs

Annotation for CpG islands was obtained from the 'cpgIslandExt' UCSC track of the hg18 assembly of the human genome database [86]. Presence or absence of TATA-box motifs in promoter regions was determined by scanning the sequence for TATA-box motifs in TRANSFAC [92] using MAST [96] with default parameters.

### Separating promoters of most highly and lowly expressed genes

#### Training data

The promoter regions (-2.5 kb to +0.5 kb with respect to the TSS, based on RefSeq annotation [85]) of the 200 most highly expressed genes (positive set) were compared to the promoters of 200 genes with the lowest expression (negative set) in each of the 79 considered tissues.

#### Sequence representation

Next, we converted the DNA sequence of each promoter into a set of TF binding site feature vectors. We first identified all CNEs (at least 70% sequence identity between human and mouse [98]) within each promoter sequence. Next, we ran the program MAST [96] with default parameters to identify motif occurrences in the CNEs matching 775 known TF binding sites from the TRANSFAC [92] and JASPAR [93-95] databases. With this information, each CNE was then transformed into a 775-dimensional TF binding site feature vector, where each feature corresponds to the number of the corresponding TF binding site occurrences in the sequence of the CNE. There were 2.4 feature vectors (one per CNE) in a promoter, on average.

For a given classifier, the training set contained as many feature vectors as the number of CNEs found in the promoters of the 200 most highly expressed genes (positive set) plus the number of CNEs found in the promoters of the 200 genes with the lowest expression (negative set). Because promoter regions may overlap, the sets included only unique CNEs.

#### Classifiers

Linear SVMs [99] were used to find features relevant to distinguish between the CNEs in promoters associated with highly expressed genes (positive class) and those in promoters associated with lowly expressed genes (negative class). For each tissue, we trained a SVM on an average of 553 feature vectors representing CNEs in the promoter regions of highly expressed genes and 525 feature vectors representing CNEs in the promoter regions of lowly expressed genes. We optimized the weight of the positive class *w*_1_ by performing a grid search. The optimal value was chosen from $w_{1}=\frac{n^{+}}{n^{-}}\gamma$, where $\gamma \in \left\{\frac{1}{3},\frac{2}{3},1,\frac{4}{3},\frac{5}{3}\right\}$, *n*^+^ is the number of signal sequences, and *n*^-^ is the number of control sequences.

A double-loop cross-validation was used to assess the accuracy of the classifier. In each fold of the cross-validation, we used four-fifths of the members of the positive and negative classes to identify a 'consistent' set within the positive class. This strategy is aimed at identifying sequences that are consistent with each other, in an effort to reduce the natural heterogeneity of the promoter sets. More precisely, in each fold of the cross-validation, for each promoter *P* in the positive class in the four-fifths of the data that was used to identify a consistent set, we trained a model excluding all sequences associated with *P*. Subsequently, we used that model to score each of the sequences associated with *P*. Finally, among those sequences, we randomly selected two positive-scoring ones to represent *P* in a 'consistent' positive set. After repeating this for all promoters in the positive class, we obtained a 'consistent' positive set. This consistent positive set was used together with the remaining one-fifth of the members of the negative class to train a final classifier. The accuracy of this final classifier was evaluated using a standard five-fold cross-validation. The entire procedure was repeated for each of the five cross-validation folds, and the cross-validation was repeated five times. AUC was used as criterion for optimality. This double-loop cross-validation has been successfully applied to the enhancer prediction problem in the past (for example, [17]).

Figure S15 in Additional file 1 illustrates the variation of the size of the consistent positive set for the 79 tissues considered. In our cross-validation framework, the consistent positive set contained an average of 157 CNEs, representing 35% of the training data. However, the size of the consistent positive set depends on the particular tissue, ranging from 39 (10%) to 269 (41%) CNEs for PB-CD56+ NK cells and medulla oblongata, respectively. For PB-CD56+ NK cells the consistent positive set also contained the smallest fraction of CNEs, while the largest fraction was obtained for uterus corpus (51%).

##### Linear SVMs

Training a linear SVM classifier is equivalent to solving the following constrained optimization problem [100]:

Given the training samples $T={\left\{\left(x_{i},y_{i}\right)|x_{i}\in {\mathbb{R}}^{p},y_{i}\in \left\{-1,1\right\}\right\}}_{i=1}^{n}$, find the values of w, b and ξ_i_ that minimize

$$\frac{1}{2}w^{T}w+C\sum_{i=1}^{n}\xi_{i}$$

satisfying the constraints

$$y_{i}\left(w^{T}x_{i}+b\right)\geq 1-\xi_{i}\forall i=1,\ldots ,n$$

and

$$\xi_{i}\geq 0\forall i=1,\ldots ,n$$

The decision function of the classifier for an unknown sample x is given by:

$$f\left(x\right)=\mathrm{sign}\left(w^{T}x+b\right)$$

The dual form of this problem can be described as follows: Given the training samples $T={\left\{\left(x_{i},y_{i}\right)|x_{i}\in {\mathbb{R}}^{p},y_{i}\in \left\{-1,1\right\}\right\}}_{i=1}^{n}$, find the values ${\left\{\alpha_{i}\right\}}_{i=1}^{n}$ that maximize

$$\sum_{i}\alpha_{i}-\frac{1}{2}\sum_{i=1}^{n}\sum_{j=1}^{n}\alpha_{i}\alpha_{j}y_{i}y_{j}x_{i}^{T}x_{j}$$

satisfying the constraints

$$0\leq \alpha_{i}\leq C\forall i=1,\ldots ,n$$

and

$$\sum_{i=1}^{n}a_{i}y_{i}=0.$$

Samples x_i_ for which a_i_ ≥ 0 are called support vectors.

The vector w can be computed in terms of α_i_ as:

$$w=\sum_{i=1}^{n}a_{i}y_{i}x_{i}$$

and, therefore, contains the weighted features of the support vectors.

##### SVM parameter selection

Linear SVMs have only one parameter, *C*, which controls the trade-off between errors on the training data and margin maximization. We found that the performance of the Hb enhancer classifier was relatively stable with respect to changes in *C*. We estimated *C* based on the training data as ${\left[\frac{1}{n}\sum_{i=1}^{n}\left|x_{i}\right|\right]}^{-2}$. Misclassifications are penalized differently depending on the class of sequences, proportionally to the total number of sequences in each class.

### Predictive power of the motifs

After obtaining a linear SVM model, the weight vector w can be used to decide the relevance of each feature [101]. The larger |w_j_|, the more important role of feature *j* in the decision function. On these grounds, we used the weights *w*_*j*_ to assess the predictive power of each motif.

#### Scaled SVM weights

To make motif weights comparable across different SVM classifiers, we scaled them preserving their sign according to:

$$\mathrm{scaled}\;w_{j}=\left\{\begin{array}{cc} -\left(1-\frac{w_{j}-w_{\min}}{-w_{\min}}\right), & \mathrm{if}\;w_{j}<0\\ \frac{w_{j}}{w_{\max}}, & \mathrm{if}\;w_{j}\geq 0 \end{array}\right),$$

where

$$w_{\min}=\left\{\begin{array}{cc} \min_{j}\left\{w_{j}\right\}, & \mathrm{if}\;\min_{j}\left\{w_{j}\right\}<0\\ 0, & \mathrm{otherwise} \end{array}\right)$$

and

$$w_{\max}=\left\{\begin{array}{cc} \max_{j}\left\{w_{j}\right\}, & \mathrm{if}\;\max_{j}\left\{w_{j}\right\}>0\\ 0, & \mathrm{otherwise} \end{array}\right)$$

### GC content of transcription factor binding sites

Sequence motifs representing motifs are usually encoded as PWMs. A PWM is a matrix containing the relative frequency of each of the four possible nucleotides at each position of a motif, which are estimates of the corresponding probabilities.

To obtain the GC content of a motif, we calculated and averaged the probability of observing G or C at each position of the corresponding PWM.

In order to assess the contribution of the GC content to the performance of the promoter-based enhancer models, we trained 5 models using the aforementioned strategy, each time replacing the original 775 PWMs by an equally large collection of PWMs, in which the nucleotide probabilities of each PWM have been randomly permuted.

### Difference in GC content between two loci

Differences in GC content between loci of highly and lowly expressed genes were expressed as the natural logarithm of the ratio between the GC content of the loci of highly expressed genes and the GC content of the loci of lowly expressed genes.

### Enhancer predictions

We applied our promoter-based models as genome-wide predictors of human enhancers to both conserved and non-conserved sequences. In particular, for a given tissue, when we refer to predictions in the loci of the (200) most highly and lowly expressed genes, we imply predictions in the loci of the 200 genes with highest and lowest expression levels whose promoters were used to train the corresponding classifier.

#### Prediction of conserved enhancers

First, we selected CNEs with at least 70% identity across the human and mouse genomes [98] located at least 2.5 kb upstream and 0.5 kb downstream of TSSs annotated in refGene.txt and knownGene.txt tables (available in the UCSC Genome Browser database [86]). Thus, we scored approximately 1,200,000 CNEs across the human genome, with an average length of 249 bp. In particular, the loci of the 200 most highly expressed genes in any of the 73 tissues considered comprised, on average, 85 CNEs, and comprised a total of 500,000 CNEs, while the loci of the 200 genes with lowest expression in any of the 73 tissues considered included an average of 108 and a total of 750,000, respectively.

#### Prediction of non-conserved enhancers

Second, we scanned the genome using a sliding window approach. Windows overlapping the sequence 2.5 kb upstream and 0.5 kb downstream of the nearest TSS according to the refGene.txt and knownGene.txt tables (available in the UCSC Genome Browser database [86]) were excluded from further analysis. For the size of the window, we chose the average length of the conserved region between human and mouse [98], namely 230 bps. The sliding window is shifted by 115 bps. A given sequence was considered an enhancer prediction (or enhancer candidate) if its score was greater than *s* = *min*(0, *δ*), where *δ* is the lowest score of the top 5% sequences scored in the control loci.

### Computational evaluation of genome-wide enhancer predictions

#### Functional analysis

To assess whether these elements disproportionally occur near genes with particular functions, we obtained the Gene Ontology [102] (CVS version 1.2811, GOC Validation Date March 28, 2012) annotations of the closest neighboring UCSC known genes [103] for all non-coding elements, and assigned those annotations to each element. Gene-to-GO mapping was achieved by combining the UCSC refGene.txt and knownGene.txt tables and GOA [104] association table using UniProt IDs. *P*-values were corrected for multiple testing using Bonferroni’s method [105].

#### Fold enrichment of enhancer predictions in the loci of the 200 most highly expressed genes as compared to the loci of lowly expressed genes

In order to account for differences in the length of the loci, we did not directly compare the number of enhancer predictions in the loci of the 200 most highly expressed genes in a given tissue with the number of enhancer predictions in the loci of lowly expressed genes in that same tissue, but the numbers of enhancer predictions divided by the numbers of scanned sequences for loci of highly and lowly expressed genes. Therefore, the fold enrichments in Table 1 and Additional file 3 were computed as the ratio of two proportions: (i) the total number of enhancers predicted in the loci of the 200 most highly expressed genes divided by the total number of sequences scanned in the loci of highly expressed genes; and (ii) the total number of enhancers predicted in the loci of lowly expressed genes divided by the total number of sequences scanned in the loci of lowly expressed genes. For the 73 tissues evaluated and focusing only on CNEs across the human and mouse genomes, these proportions averaged 0.04 for loci of highly expressed genes, and 0.03 for loci of lowly expressed genes. In the case of whole-loci predictions, these proportions averaged 0.03 for loci of the 200 most highly expressed genes, and 0.02 for loci of lowly expressed genes.

#### Fraction of loci comprising enhancer predictions

The fraction of loci comprising enhancer predictions was defined as the number of loci in which at least one of the scanned sequences was considered an enhancer prediction divided by the total number of loci to which we applied the classifier. Therefore, the fold enrichments in Table 1 and Additional file 3 were computed as the ratio of two ratios: (i) the total number of loci of highly expressed genes comprising at least one enhancer prediction each divided by the total number of loci of highly expressed genes comprising at least one scanned sequence each; and (ii) the total number of loci of lowly expressed genes comprising at least one enhancer prediction each divided by the total number of loci of lowly expressed genes comprising at least one scanned sequence each. Each of the latter ratios ranges between 0 (no loci comprising enhancer predictions) and 1 (all loci comprising scanned sequences also comprise enhancer predictions). For the 73 tissues evaluated and focusing only on CNEs across the human and mouse genomes, 59% of the loci of highly expressed genes comprised at least one enhancer prediction, while 52% of the loci of lowly expressed genes did.

#### Overlap between predictions and different enhancer marks

Predictions resulting from the 73 reliable promoter-based classifiers were combined into a set of non-redundant predictions and overlapped with different enhancer marks. Additionally, when specifically stated, we report overlaps with predictions for particular promoter-based classifiers - for example, the classifier trained on liver promoters.

##### Overlap with p300

Genomic regions enriched for p300 in mouse forebrain, midbrain, limb, and heart tissues were extracted from Additional files 3, 4 and 5[45], and mapped to the human genome (hg18) using LiftOver [106]. Genomic regions identified in forebrain, midbrain, limb, and heart were combined into one dataset. Overlapping genomic regions were clustered together.

##### Overlap with DNase I hypersensitivity sites

DNase I hypersensitivity data ('narrow peaks') for 86 human cell lines from the ENCODE project [25,107] were downloaded from the UCSC browser [108,109], converted to the hg18 assembly using LiftOver [106], and combined into one dataset. Overlapping genomic regions were clustered together. This resulted in a total of 1,722,559 non-overlapping regions with an average length of 253 bp. We then computed the intersection between the set of non-redundant enhancer predictions identified by any of the 73 promoter-based models and this DNase I hypersensitivity data dataset. Liver enhancer predictions, in particular, were also compared with DNase I hypersensitivity data in HepG2. Predictions for enhancers in other tissues were compared with DNase I hypersensitivity data in closely related ENCODE tissues and cell lines (Figure S9 in Additional file 1).

##### Overlap with histone modification marks

Histone mark data (H3K4me1, H3K27ac) for 11 human cell lines from the ENCODE project [25,107] were downloaded from the UCSC browser [108,110], converted to the hg18 assembly using LiftOver [106], and combined into one dataset. Overlapping genomic regions were clustered together. This resulted in a total of 189,889 non-overlapping regions with an average length of 6,275 bp. We then computed the intersection between the set of non-redundant enhancer predictions identified by any of the 73 promoter-based models and this histone mark dataset. Liver enhancer predictions, in particular, were also compared with histone marks in HepG2.

##### Overlap with ChromHMM predictions

Weak and strong enhancers identified in nine human cell lines (HSMM, GM12878, HUVEC, H1-hESC, K562, HepG2, NHEK, HMEC, NHLF) using ChromHMM [51] were downloaded from the UCSC browser [108,111], converted to the hg18 assembly using LiftOver [106], and combined into one dataset. Overlapping genomic regions were clustered together. This resulted in a total of 399,500 non-overlapping regions with an average length of 1,504 bp. We then computed the intersection between the set of non-redundant enhancer predictions identified by any of the 73 promoter-based models and the ChromHMM dataset. Liver enhancer predictions, in particular, were also compared with ChromHMM enhancers in HepG2.

#### Conservation analysis

PhastCons conservation scores [56] were based on alignment of 28 vertebrate species and an 18 species placental mammal subset, respectively [55].

#### In vivo validation *of liver enhancer predictions*

Sequences selected for *in vivo* validation were PCR-amplified using TopTaq (Qiagen, Hilden, Germany) from human genomic DNA (Roche, Basel, Switzerland), purified using the QIAquick PCR purification kit (Qiagen) and cloned into the pENTR-dTOPO vector (Life Technologies, Carlsbad, CA, USA). Proper insertion and orientation was confirmed by colony PCR, after which positive clones were transferred into the pGL4.23[luc2] vector (Promega) using the Gateway system (Life Technologies). Sequence and orientation of the insert were re-verified by Sanger sequencing, and approximately 200 μg of endotoxin-free plasmid DNA was isolated using the EndoFree Plasmid Midi prep (Qiagen).

For the hydrodynamic tail vein assay, 10 μg of each assayed sequence in pGL4.23[luc2] was injected along with 2 μg of pGL4.74[hRluc/TK] vector to correct for injection efficiency, into at least three CD1 mice (Charles River Laboratories, Wilmington, MA, USA) using the TransIT EE hydrodynamic gene delivery system (Mirus Bio LCC, Madison, WI, USA) according to the manufacturer’s protocol. Negative (empty pGL4.23[luc2]) and positive (*ApoE* liver enhancer [110,112]) controls (n = 3 to 5) were also injected at each injection date/experiment. After 24 hours, livers were harvested and homogenized in passive lysis buffer (Promega), followed by centrifugation at 4°C for 30 minutes at 14,000 rpm. Firefly and Renilla luciferase activity in the supernatant (diluted 1:20) were measured on a Synergy 2 microplate reader (BioTek Instruments, Winooski, VT, USA) in technical replicates of four for each liver, using the Dual-Luciferase reporter assay system (Promega). The ratios for firefly luciferase:Renilla luciferase were determined and expressed as relative luciferase activity. All mouse work was approved by the UCSF Institutional Animal Care and Use Committee.

## Abbreviations

AUC: Area under the receiver operating characteristic curve; bp: Base pair; CNE: Conserved non-coding element; DHS: DNase I hypersensitive site; NK: Natural killer; PWM: Position-weight matrix; SVM: Support vector machine; TF: Transcription factor; TSS: Transcription start site; UTR: Untranslated region.

## Competing interests

The authors declare that they have no competing interests.

## Authors’ contributions

IO conceived the work and supervised the analysis. LT carried out the analysis. RPS and MJK conducted the mouse experiments. NA supervised the experiments. The manuscript was written by LT and IO, with contributions from the other authors. All authors read and approved the final manuscript.

## Supplementary Material

Additional file 1 Figures S1 to 15 and Supplementary notes. Click here for file

Additional file 2: Table S1 A table listing the motif ranks for the promoter-based models. Click here for file

Additional file 3: Table S2 A table summarizing the performance of the promoter-based models. Click here for file

Additional file 4: Table S3 A summary of results obtained with the hydrodynamic tail vein injection assay. Click here for file

Additional file 5: Table S4 A list of transcription factors known to be relevant for liver function. Click here for file

## References

[B1] Clamp M Fry B Kamal M Xie X Cuff J Lin MF Kellis M Lindblad-Toh K Lander ES Distinguishing protein-coding and noncoding genes in the human genome Proc Natl Acad Sci U S A 2007 14 19428 19433 10.1073/pnas.0709013104 18040051 PMC2148306

[B2] Smale ST Kadonaga JT The RNA polymerase II core promoter Annu Rev Biochem 2003 14 449 479 10.1146/annurev.biochem.72.121801.161520 12651739

[B3] Sandelin A Carninci P Lenhard B Ponjavic J Hayashizaki Y Hume DA Mammalian RNA polymerase II core promoters: insights from genome-wide studies Nat Rev Genet 2007 14 424 436 17486122 10.1038/nrg2026

[B4] Kagey MH Newman JJ Bilodeau S Zhan Y Orlando DA van Berkum NL Ebmeier CC Goossens J Rahl PB Levine SS Taatjes DJ Dekker J Young RA Mediator and cohesin connect gene expression and chromatin architecture Nature 2010 14 430 435 10.1038/nature09380 20720539 PMC2953795

[B5] Dixon JR Selvaraj S Yue F Kim A Li Y Shen Y Hu M Liu JS Ren B Topological domains in mammalian genomes identified by analysis of chromatin interactions Nature 2012 14 376 380 10.1038/nature11082 22495300 PMC3356448

[B6] Nora EP Lajoie BR Schulz EG Giorgetti L Okamoto I Servant N Piolot T van Berkum NL Meisig J Sedat J Gribnau J Barillot E Bluthgen N Dekker J Heard E Spatial partitioning of the regulatory landscape of the X-inactivation centre Nature 2012 14 381 385 10.1038/nature11049 22495304 PMC3555144

[B7] Maniatis T Goodbourn S Fischer JA Regulation of inducible and tissue-specific gene expression Science 1987 14 1237 1245 10.1126/science.3296191 3296191

[B8] Maston GA Evans SK Green MR Transcriptional regulatory elements in the human genome Annu Rev Genomics Hum Genet 2006 14 29 59 10.1146/annurev.genom.7.080505.115623 16719718

[B9] Sakabe NJ Nobrega MA Genome-wide maps of transcription regulatory elements Wiley Interdiscip Rev Syst Biol Med 2010 14 422 437 10.1002/wsbm.70 20836039

[B10] Noonan JP McCallion AS Genomics of long-range regulatory elements Annu Rev Genomics Hum Genet 2010 14 1 23 10.1146/annurev-genom-082509-141651 20438361

[B11] Roider HG Lenhard B Kanhere A Haas SA Vingron M CpG-depleted promoters harbor tissue-specific transcription factor binding signals - implications for motif overrepresentation analyses Nucleic Acids Res 2009 14 6305 6315 10.1093/nar/gkp682 19736212 PMC2770660

[B12] Soler E Andrieu-Soler C de Boer E Bryne JC Thongjuea S Stadhouders R Palstra RJ Stevens M Kockx C van Ijcken W Hou J Steinhoff C Rijkers E Lenhard B Grosveld F The genome-wide dynamics of the binding of Ldb1 complexes during erythroid differentiation Genes Dev 2010 14 277 289 10.1101/gad.551810 20123907 PMC2811829

[B13] Landolin JM Johnson DS Trinklein ND Aldred SF Medina C Shulha H Weng Z Myers RM Sequence features that drive human promoter function and tissue specificity Genome Res 2010 14 890 898 10.1101/gr.100370.109 20501695 PMC2892090

[B14] Smith AD Sumazin P Xuan Z Zhang MQ DNA motifs in human and mouse proximal promoters predict tissue-specific expression Proc Natl Acad Sci U S A 2006 14 6275 6280 10.1073/pnas.0508169103 16606849 PMC1458868

[B15] Gorkin DU Lee D Reed X Fletez-Brant C Bessling SL Loftus SK Beer MA Pavan WJ McCallion AS Integration of ChIP-seq and machine learning reveals enhancers and a predictive regulatory sequence vocabulary in melanocytes Genome Res 2012 14 2290 2301 10.1101/gr.139360.112 23019145 PMC3483558

[B16] Lee D Karchin R Beer MA Discriminative prediction of mammalian enhancers from DNA sequence Genome Res 2011 14 2167 2180 10.1101/gr.121905.111 21875935 PMC3227105

[B17] Narlikar L Sakabe NJ Blanski AA Arimura FE Westlund JM Nobrega MA Ovcharenko I Genome-wide discovery of human heart enhancers Genome Res 2010 14 381 392 10.1101/gr.098657.109 20075146 PMC2840982

[B18] Burzynski GM Reed X Taher L Stine ZE Matsui T Ovcharenko I McCallion AS Systematic elucidation and in vivo validation of sequences enriched in hindbrain transcriptional control Genome Res 2012 14 2278 2289 10.1101/gr.139717.112 22759862 PMC3483557

[B19] Vavouri T McEwen GK Woolfe A Gilks WR Elgar G Defining a genomic radius for long-range enhancer action: duplicated conserved non-coding elements hold the key Trends Genet 2006 14 5 10 10.1016/j.tig.2005.10.005 16290136

[B20] Lettice LA Heaney SJ Purdie LA Li L de Beer P Oostra BA Goode D Elgar G Hill RE de Graaff E A long-range Shh enhancer regulates expression in the developing limb and fin and is associated with preaxial polydactyly Hum Mol Genet 2003 14 1725 1735 10.1093/hmg/ddg180 12837695

[B21] Gordon CT Tan TY Benko S Fitzpatrick D Lyonnet S Farlie PG Long-range regulation at the SOX9 locus in development and disease J Med Genet 2009 14 649 656 10.1136/jmg.2009.068361 19473998

[B22] Bagheri-Fam S Barrionuevo F Dohrmann U Gunther T Schule R Kemler R Mallo M Kanzler B Scherer G Long-range upstream and downstream enhancers control distinct subsets of the complex spatiotemporal Sox9 expression pattern Dev Biol 2006 14 382 397 10.1016/j.ydbio.2005.11.013 16458883

[B23] Thurman RE Rynes E Humbert R Vierstra J Maurano MT Haugen E Sheffield NC Stergachis AB Wang H Vernot B Garg K John S Sandstrom R Bates D Boatman L Canfield TK Diegel M Dunn D Ebersol AK Frum T Giste E Johnson AK Johnson EM Kutyavin T Lajoie B Lee BK Lee K London D Lotakis D Neph S The accessible chromatin landscape of the human genome Nature 2012 14 75 82 10.1038/nature11232 22955617 PMC3721348

[B24] Sanyal A Lajoie BR Jain G Dekker J The long-range interaction landscape of gene promoters Nature 2012 14 109 113 10.1038/nature11279 22955621 PMC3555147

[B25] Bernstein BE Birney E Dunham I Green ED Gunter C Snyder M An integrated encyclopedia of DNA elements in the human genome Nature 2012 14 57 74 10.1038/nature11247 22955616 PMC3439153

[B26] Jacox E Gotea V Ovcharenko I Elnitski L Tissue-specific and ubiquitous expression patterns from alternative promoters of human genes PLoS One 2010 14 e12274 10.1371/journal.pone.0012274 20806066 PMC2923625

[B27] Chen X Wu JM Hornischer K Kel A Wingender E TiProD: the Tissue-specific Promoter Database Nucleic Acids Res 2006 14 D104 D107 10.1093/nar/gkj113 16381824 PMC1347475

[B28] Davuluri RV Suzuki Y Sugano S Plass C Huang TH The functional consequences of alternative promoter use in mammalian genomes Trends Genet 2008 14 167 177 10.1016/j.tig.2008.01.008 18329129

[B29] Shen Y Yue F McCleary DF Ye Z Edsall L Kuan S Wagner U Dixon J Lee L Lobanenkov VV Ren B A map of the cis-regulatory sequences in the mouse genome Nature 2012 14 116 120 10.1038/nature11243 22763441 PMC4041622

[B30] Su AI Wiltshire T Batalov S Lapp H Ching KA Block D Zhang J Soden R Hayakawa M Kreiman G Cooke MP Walker JR Hogenesch JB A gene atlas of the mouse and human protein-encoding transcriptomes Proc Natl Acad Sci U S A 2004 14 6062 6067 10.1073/pnas.0400782101 15075390 PMC395923

[B31] Robinson PN Bohme U Lopez R Mundlos S Nurnberg P Gene-Ontology analysis reveals association of tissue-specific 5' CpG-island genes with development and embryogenesis Hum Mol Genet 2004 14 1969 1978 10.1093/hmg/ddh207 15254011

[B32] Gardiner-Garden M Frommer M Transcripts and CpG islands associated with the pro-opiomelanocortin gene and other neurally expressed genes J Mol Endocrinol 1994 14 365 382 10.1677/jme.0.0120365 7916974

[B33] Aoyama T Peters JM Iritani N Nakajima T Furihata K Hashimoto T Gonzalez FJ Altered constitutive expression of fatty acid-metabolizing enzymes in mice lacking the peroxisome proliferator-activated receptor alpha (PPARalpha) J Biol Chem 1998 14 5678 5684 10.1074/jbc.273.10.5678 9488698

[B34] Pawar A Botolin D Mangelsdorf DJ Jump DB The role of liver X receptor-alpha in the fatty acid regulation of hepatic gene expression J Biol Chem 2003 14 40736 40743 10.1074/jbc.M307973200 12917410

[B35] Zhang P Bennoun M Gogard C Bossard P Leclerc I Kahn A Vasseur-Cognet M Expression of COUP-TFII in metabolic tissues during development Mech Dev 2002 14 109 114 10.1016/S0925-4773(02)00286-1 12385758

[B36] Sladek FM Zhong WM Lai E Darnell JE Jr Liver-enriched transcription factor HNF-4 is a novel member of the steroid hormone receptor superfamily Genes Dev 1990 14 2353 2365 10.1101/gad.4.12b.2353 2279702

[B37] Schmidt D Wilson MD Ballester B Schwalie PC Brown GD Marshall A Kutter C Watt S Martinez-Jimenez CP Mackay S Talianidis I Flicek P Odom D Five-vertebrate ChIP-seq reveals the evolutionary dynamics of transcription factor binding Science 2010 14 1036 1040 10.1126/science.1186176 20378774 PMC3008766

[B38] Odom DT Dowell RD Jacobsen ES Gordon W Danford TW MacIsaac KD Rolfe PA Conboy CM Gifford DK Fraenkel E Tissue-specific transcriptional regulation has diverged significantly between human and mouse Nat Genet 2007 14 730 732 10.1038/ng2047 17529977 PMC3797512

[B39] Boyle AP Davis S Shulha HP Meltzer P Margulies EH Weng Z Furey TS Crawford GE High-resolution mapping and characterization of open chromatin across the genome Cell 2008 14 311 322 10.1016/j.cell.2007.12.014 18243105 PMC2669738

[B40] May D Blow MJ Kaplan T McCulley DJ Jensen BC Akiyama JA Holt A Plajzer-Frick I Shoukry M Wright C Afzal V Simpson PC Rubin EM Black BL Bristow J Pennacchio LA Visel A Large-scale discovery of enhancers from human heart tissue Nat Genet 2012 14 89 93 10.1038/ng.1006 PMC3246570 22138689

[B41] Cotney J Leng J Yin J Reilly SK Demare LE Emera D Ayoub AE Rakic P Noonan JP The evolution of lineage-specific regulatory activities in the human embryonic limb Cell 2013 14 185 196 10.1016/j.cell.2013.05.056 23827682 PMC3785101

[B42] Hardison RC Taylor J Genomic approaches towards finding cis-regulatory modules in animals Nat Rev Genet 2012 14 469 483 10.1038/nrg3242 22705667 PMC3541939

[B43] Pennacchio LA Loots GG Nobrega MA Ovcharenko I Predicting tissue-specific enhancers in the human genome Genome Res 2007 14 201 211 10.1101/gr.5972507 17210927 PMC1781352

[B44] Visel A Rubin EM Pennacchio LA Genomic views of distant-acting enhancers Nature 2009 14 199 205 10.1038/nature08451 19741700 PMC2923221

[B45] Visel A Blow MJ Li Z Zhang T Akiyama JA Holt A Plajzer-Frick I Shoukry M Wright C Chen F Afzal V Ren B Rubin EM Pennacchio LA ChIP-seq accurately predicts tissue-specific activity of enhancers Nature 2009 14 854 858 10.1038/nature07730 19212405 PMC2745234

[B46] Ahuja P Zhao P Angelis E Ruan H Korge P Olson A Wang Y Jin ES Jeffrey FM Portman M Maclellan WR Myc controls transcriptional regulation of cardiac metabolism and mitochondrial biogenesis in response to pathological stress in mice J Clin Invest 2010 14 1494 1505 10.1172/JCI38331 20364083 PMC2860901

[B47] Egea M Meton I Baanante IV Sp1 and Sp3 regulate glucokinase gene transcription in the liver of gilthead sea bream (Sparus aurata) J Mol Endocrinol 2007 14 481 492 10.1677/jme.1.02176 17446237

[B48] Odom DT Zizlsperger N Gordon DB Bell GW Rinaldi NJ Murray HL Volkert TL Schreiber J Rolfe PA Gifford DK Fraenkel E Bell GI Young RA Control of pancreas and liver gene expression by HNF transcription factors Science 2004 14 1378 1381 10.1126/science.1089769 14988562 PMC3012624

[B49] Santos DG Resende MF Mill JG Mansur AJ Krieger JE Pereira AC Nuclear Factor (NF) kappaB polymorphism is associated with heart function in patients with heart failure BMC Med Genet 2010 14 89 20534156 10.1186/1471-2350-11-89 PMC2897791

[B50] Griffith OL Montgomery SB Bernier B Chu B Kasaian K Aerts S Mahony S Sleumer MC Bilenky M Haeussler M Griffith M Gallo SM Giardine B Hooghe B Van Loo P Blanco E Ticoll A Lithwick S Portales-Casamar E Donaldson IJ Robertson G Wadelius C De Bleser P Vlieghe D Halfon MS Wasserman W Hardison R Bergman CM Jones SJ ORegAnno: an open-access community-driven resource for regulatory annotation Nucleic Acids Res 2008 14 D107 D113 10.1093/nar/gkn457 18006570 PMC2239002

[B51] Ernst J Kheradpour P Mikkelsen TS Shoresh N Ward LD Epstein CB Zhang X Wang L Issner R Coyne M Ku M Durham T Kellis M Bernstein BE Mapping and analysis of chromatin state dynamics in nine human cell types Nature 2011 14 43 49 10.1038/nature09906 21441907 PMC3088773

[B52] Blow MJ McCulley DJ Li Z Zhang T Akiyama JA Holt A Plajzer-Frick I Shoukry M Wright C Chen F Afzal V Bristow J Ren B Black BL Rubin EM Visel A Pennacchio LA ChIP-Seq identification of weakly conserved heart enhancers Nat Genet 2010 14 806 810 10.1038/ng.650 20729851 PMC3138496

[B53] Zhang G Budker V Wolff JA High levels of foreign gene expression in hepatocytes after tail vein injections of naked plasmid DNA Hum Gene Ther 1999 14 1735 1737 10.1089/10430349950017734 10428218

[B54] Kim MJ Skewes-Cox P Fukushima H Hesselson S Yee SW Ramsey LB Nguyen L Eshragh JL Castro RA Wen CC Stryke D Johns SJ Ferrin TE Kwok PY Relling MV Giacomini KM Kroetz DL Ahituv N Functional characterization of liver enhancers that regulate drug-associated transporters Clin Pharmacol Ther 2011 14 571 578 10.1038/clpt.2010.353 21368754 PMC3227682

[B55] Miller W Rosenbloom K Hardison RC Hou M Taylor J Raney B Burhans R King DC Baertsch R Blankenberg D Kosakovsky Pond SL Nekrutenko A Giardine B Harris RS Tyekucheva S Diekhans M Pringle TH Murphy WJ Lesk A Weinstock GM Lindblad-Toh K Gibbs RA Lander ES Siepel A Haussler D Kent WJ 28-way vertebrate alignment and conservation track in the UCSC Genome Browser Genome Res 2007 14 1797 1808 10.1101/gr.6761107 17984227 PMC2099589

[B56] Siepel A Bejerano G Pedersen JS Hinrichs AS Hou M Rosenbloom K Clawson H Spieth J Hillier LW Richards S Weinstock GM Wilson RK Gibbs RA Kent WJ Miller W Haussler D Evolutionarily conserved elements in vertebrate, insect, worm, and yeast genomes Genome Res 2005 14 1034 1050 10.1101/gr.3715005 16024819 PMC1182216

[B57] Levine M Tjian R Transcription regulation and animal diversity Nature 2003 14 147 151 10.1038/nature01763 12853946

[B58] MacIsaac KD Lo KA Gordon W Motola S Mazor T Fraenkel E A quantitative model of transcriptional regulation reveals the influence of binding location on expression PLoS Comput Biol 2010 14 e1000773 10.1371/journal.pcbi.1000773 20442865 PMC2861697

[B59] Gisselbrecht SS Barrera LA Porsch M Aboukhalil A Estep PW 3rd Vedenko A Palagi A Kim Y Zhu X Busser BW Gamble CE Iagovitina A Singhania A Michelson AM Bulyk ML Highly parallel assays of tissue-specific enhancers in whole Drosophila embryos Nat Methods 2013 14 774 780 10.1038/nmeth.2558 23852450 PMC3733245

[B60] Uhlen M Oksvold P Fagerberg L Lundberg E Jonasson K Forsberg M Zwahlen M Kampf C Wester K Hober S Wernerus H Björling L Ponten F Towards a knowledge-based Human Protein Atlas Nat Biotechnol 2010 14 1248 1250 10.1038/nbt1210-1248 21139605

[B61] Robbins MJ Michalovich D Hill J Calver AR Medhurst AD Gloger I Sims M Middlemiss DN Pangalos MN Molecular cloning and characterization of two novel retinoic acid-inducible orphan G-protein-coupled receptors (GPRC5B and GPRC5C) Genomics 2000 14 8 18 10.1006/geno.2000.6226 10945465

[B62] Li XY MacArthur S Bourgon R Nix D Pollard DA Iyer VN Hechmer A Simirenko L Stapleton M Luengo Hendriks CL Chu HC Ogawa N Inwood W Sementchenko V Beaton A Weiszmann R Celniker SE Knowles DW Gingeras T Speed TP Eisen MB Biggin MD Transcription factors bind thousands of active and inactive regions in the Drosophila blastoderm PLoS Biol 2008 14 e27 10.1371/journal.pbio.0060027 18271625 PMC2235902

[B63] Yamamoto T Shimano H Inoue N Nakagawa Y Matsuzaka T Takahashi A Yahagi N Sone H Suzuki H Toyoshima H Yamada N Protein kinase A suppresses sterol regulatory element-binding protein-1C expression via phosphorylation of liver X receptor in the liver J Biol Chem 2007 14 11687 11695 17296605 10.1074/jbc.M611911200

[B64] Jongens TA Fowler T Shermoen AW Beckendorf SK Functional redundancy in the tissue-specific enhancer of the Drosophila Sgs-4 gene EMBO J 1988 14 2559 2567 3142764 10.1002/j.1460-2075.1988.tb03105.x PMC457128

[B65] Hoch M Schröder C Seifert E Jäckle H Cis-acting control elements for Krüppel expression in the Drosophila embryo EMBO J 1990 14 2587 2595 2114978 10.1002/j.1460-2075.1990.tb07440.x PMC552291

[B66] Kassis JA Spatial and temporal control elements of the Drosophila engrailed gene Genes Dev 1990 14 433 443 10.1101/gad.4.3.433 2110923

[B67] Hong JW Hendrix DA Levine MS Shadow enhancers as a source of evolutionary novelty Science 2008 14 1314 10.1126/science.1160631 18772429 PMC4257485

[B68] Perry MW Boettiger AN Bothma JP Levine M Shadow enhancers foster robustness of Drosophila gastrulation Curr Biol 2010 14 1562 1567 10.1016/j.cub.2010.07.043 20797865 PMC4257487

[B69] Dunipace L Ozdemir A Stathopoulos A Complex interactions between cis-regulatory modules in native conformation are critical for Drosophila snail expression Development 2011 14 4075 4084 10.1242/dev.069146 21813571 PMC3160101

[B70] Guerrero L Marco-Ferreres R Serrano AL Arredondo JJ Cervera M Secondary enhancers synergise with primary enhancers to guarantee fine-tuned muscle gene expression Dev Biol 2010 14 16 28 10.1016/j.ydbio.2009.10.006 19835855

[B71] Frankel N Davis GK Vargas D Wang S Payre F Stern DL Phenotypic robustness conferred by apparently redundant transcriptional enhancers Nature 2010 14 490 493 10.1038/nature09158 20512118 PMC2909378

[B72] Akalin A Fredman D Arner E Dong X Bryne JC Suzuki H Daub CO Hayashizaki Y Lenhard B Transcriptional features of genomic regulatory blocks Genome Biol 2009 14 R38 10.1186/gb-2009-10-4-r38 19374772 PMC2688929

[B73] Engström PG Ho Sui SJ Drivenes O Becker TS Lenhard B Genomic regulatory blocks underlie extensive microsynteny conservation in insects Genome Res 2007 14 1898 1908 10.1101/gr.6669607 17989259 PMC2099597

[B74] Woolfe A Goodson M Goode DK Snell P McEwen GK Vavouri T Smith SF North P Callaway H Kelly K Walter K Abnizova I Gilks W Edwards YJ Cooke JE Elgar G Highly conserved non-coding sequences are associated with vertebrate development PLoS Biol 2005 14 e7 10.1371/journal.pbio.0030007 15630479 PMC526512

[B75] Nobrega MA Ovcharenko I Afzal V Rubin EM Scanning human gene deserts for long-range enhancers Science 2003 14 413 10.1126/science.1088328 14563999

[B76] Kikuta H Laplante M Navratilova P Komisarczuk AZ Engström PG Fredman D Akalin A Caccamo M Sealy I Howe K Ghislain J Pezeron G Mourrain P Ellingsen S Oates AC Thisse C Thisse B Foucher I Adolf B Geling A Lenhard B Becker TS Genomic regulatory blocks encompass multiple neighboring genes and maintain conserved synteny in vertebrates Genome Res 2007 14 545 555 10.1101/gr.6086307 17387144 PMC1855176

[B77] Sandelin A Bailey P Bruce S Engström PG Klos JM Wasserman WW Ericson J Lenhard B Arrays of ultraconserved non-coding regions span the loci of key developmental genes in vertebrate genomes BMC Genomics 2004 14 99 10.1186/1471-2164-5-99 15613238 PMC544600

[B78] Montavon T Soshnikova N Mascrez B Joye E Thevenet L Splinter E de Laat W Spitz F Duboule D A regulatory archipelago controls Hox genes transcription in digits Cell 2011 14 1132 1145 10.1016/j.cell.2011.10.023 22118467

[B79] Pennacchio LA Ahituv N Moses AM Prabhakar S Nobrega MA Shoukry M Minovitsky S Dubchak I Holt A Lewis KD Plajzer-Frick I Akiyama J De Val S Afzal V Black BL Couronne O Eisen MB Visel A Rubin EM In vivo enhancer analysis of human conserved non-coding sequences Nature 2006 14 499 502 10.1038/nature05295 17086198

[B80] Natarajan A Yardimci GG Sheffield NC Crawford GE Ohler U Predicting cell-type-specific gene expression from regions of open chromatin Genome Res 2012 14 1711 1722 10.1101/gr.135129.111 22955983 PMC3431488

[B81] Schug J Schuller WP Kappen C Salbaum JM Bucan M Stoeckert CJ Jr Promoter features related to tissue specificity as measured by Shannon entropy Genome Biol 2005 14 R33 10.1186/gb-2005-6-4-r33 15833120 PMC1088961

[B82] Merika M Thanos D Enhanceosomes Curr Opin Genet Dev 2001 14 205 208 10.1016/S0959-437X(00)00180-5 11250145

[B83] The ENCODE Project Consortium The ENCODE (ENCyclopedia Of DNA Elements) Project Science 2004 14 636 640 15499007 10.1126/science.1105136

[B84] 1000 Genomes Project Consortium A map of human genome variation from population-scale sequencing Nature 2010 14 1061 1073 10.1038/nature09534 20981092 PMC3042601

[B85] Pruitt KD Tatusova T Maglott DR NCBI reference sequences (RefSeq): a curated non-redundant sequence database of genomes, transcripts and proteins Nucleic Acids Res 2007 14 D61 D65 10.1093/nar/gkl842 17130148 PMC1716718

[B86] Kent WJ Sugnet CW Furey TS Roskin KM Pringle TH Zahler AM Haussler D The human genome browser at UCSC Genome Res 2002 14 996 1006 12045153 10.1101/gr.229102 PMC186604

[B87] Rodelsperger C Guo G Kolanczyk M Pletschacher A Kohler S Bauer S Schulz MH Robinson PN Integrative analysis of genomic, functional and protein interaction data predicts long-range enhancer-target gene interactions Nucleic Acids Res 2011 14 2492 2502 10.1093/nar/gkq1081 21109530 PMC3074119

[B88] gnfAtlas2.txt.gz [ftp://hgdownload.cse.ucsc.edu/goldenPath/hg18/database/gnfAtlas2.txt.gz]

[B89] knownToGnfAtlas2.txt.gz [ftp://hgdownload.cse.ucsc.edu/goldenPath/hg18/database/knownToGnfAtlas2.txt.gz]

[B90] kgXref.txt.gz [ftp://hgdownload.cse.ucsc.edu/goldenPath/hg18/database/kgXref.txt.gz]

[B91] Geer LY Marchler-Bauer A Geer RC Han L He J He S Liu C Shi W Bryant SH The NCBI BioSystems database Nucleic Acids Res 2010 14 D492 D496 10.1093/nar/gkp858 19854944 PMC2808896

[B92] Matys V Kel-Margoulis OV Fricke E Liebich I Land S Barre-Dirrie A Reuter I Chekmenev D Krull M Hornischer K Voss N Stegmaier P Lewicki-Potapov B Saxel H Kel AE Wingender E TRANSFAC and its module TRANSCompel: transcriptional gene regulation in eukaryotes Nucleic Acids Res 2006 14 D108 D110 10.1093/nar/gkj143 16381825 PMC1347505

[B93] Sandelin A Alkema W Engstrom P Wasserman WW Lenhard B JASPAR: an open-access database for eukaryotic transcription factor binding profiles Nucleic Acids Res 2004 14 D91 D94 10.1093/nar/gkh012 14681366 PMC308747

[B94] Vlieghe D Sandelin A De Bleser PJ Vleminckx K Wasserman WW van Roy F Lenhard B A new generation of JASPAR, the open-access repository for transcription factor binding site profiles Nucleic Acids Res 2006 14 D95 D97 10.1093/nar/gkj115 16381983 PMC1347477

[B95] Bryne JC Valen E Tang MH Marstrand T Winther O da Piedade I Krogh A Lenhard B Sandelin A JASPAR, the open access database of transcription factor-binding profiles: new content and tools in the 2008 update Nucleic Acids Res 2008 14 D102 D106 10.1093/nar/gkn449 18006571 PMC2238834

[B96] Bailey TL Gribskov M Methods and statistics for combining motif match scores J Comput Biol 1998 14 211 221 10.1089/cmb.1998.5.211 9672829

[B97] Subramanian A Tamayo P Mootha VK Mukherjee S Ebert BL Gillette MA Paulovich A Pomeroy SL Golub TR Lander ES Mesirov JP Gene set enrichment analysis: a knowledge-based approach for interpreting genome-wide expression profiles Proc Natl Acad Sci U S A 2005 14 15545 15550 10.1073/pnas.0506580102 16199517 PMC1239896

[B98] Loots G Ovcharenko I ECRbase: database of evolutionary conserved regions, promoters, and transcription factor binding sites in vertebrate genomes Bioinformatics 2007 14 122 124 10.1093/bioinformatics/btl546 17090579

[B99] LIBSVM - A Library for Support Vector Machines [http://www.csie.ntu.edu.tw/~cjlin/libsvm]

[B100] Shawe-Taylor J Cristianini N On the generalisation of soft margin algorithms IEEE Trans Inf Theory 2002 14 2721 2735 10.1109/TIT.2002.802647

[B101] Guyon I Weston J Barnhill S Vapnik V Gene selection for cancer classification using support vector machines Mach Learn 2002 14 389 422 10.1023/A:1012487302797

[B102] Ashburner M Ball CA Blake JA Botstein D Butler H Cherry JM Davis AP Dolinski K Dwight SS Eppig JT Harris MA Hill DP Issel-Tarver L Kasarskis A Lewis S Matese JC Richardson JE Ringwald M Rubin GM Sherlock G Gene ontology: tool for the unification of biology, The Gene Ontology Consortium Nat Genet 2000 14 25 29 10.1038/75556 10802651 PMC3037419

[B103] Hsu F Kent WJ Clawson H Kuhn RM Diekhans M Haussler D The UCSC Known Genes Bioinformatics 2006 14 1036 1046 10.1093/bioinformatics/btl048 16500937

[B104] Barrell D Dimmer E Huntley RP Binns D O’Donovan C Apweiler R The GOA database in 2009–an integrated Gene Ontology Annotation resource Nucleic Acids Res 2009 14 396 403 10.1093/nar/gkn803 PMC2686469 18957448

[B105] Abdi H Salkind NJ Bonferroni and Sidak corrections for multiple comparisons Encyclopedia of Measurement and Statistics 2007 Thousand Oaks, CA: Sage Publications 103 107

[B106] Hinrichs AS Karolchik D Baertsch R Barber GP Bejerano G Clawson H Diekhans M Furey TS Harte RA Hsu F Hillman-Jackson J Kuhn RM Pedersen JS Pohl A Raney BJ Rosenbloom KR Siepel A Smith KE Sugnet CW Sultan-Qurraie A Thomas DJ Trumbower H Weber RJ Weirauch M Zweig AS Haussler D Kent WJ The UCSC Genome Browser Database: update 2006 Nucleic Acids Res 2006 14 D590 D598 10.1093/nar/gkj144 16381938 PMC1347506

[B107] ENCODE Project Consortium A user’s guide to the encyclopedia of DNA elements (ENCODE) PLoS Biol 2011 14 e1001046 10.1371/journal.pbio.1001046 21526222 PMC3079585

[B108] Rosenbloom KR Sloan CA Malladi VS Dreszer TR Learned K Kirkup VM Wong MC Maddren M Fang R Heitner SG Lee BT Barber GP Harte RA Diekhans M Long JC Wilder SP Zweig AS Karolchik D Kuhn RM Haussler D Kent WJ ENCODE data in the UCSC Genome Browser: year 5 update Nucleic Acids Res 2013 14 D56 D63 10.1093/nar/gks1172 23193274 PMC3531152

[B109] DNase I Hypersensitivity by Digital DNase I from ENCODE/University of Washington [http://hgdownload.cse.ucsc.edu/goldenPath/hg19/encodeDCC/wgEncodeUwDnase/]

[B110] Histone ChIP-seq dataset from ENCODE/Broad Institute [http://hgdownload.cse.ucsc.edu/goldenPath/hg19/encodeDCC/wgEncodeBroadHistone/]

[B111] HMM chromatin state maps from ENCODE/Broad Institute [http://hgdownload.cse.ucsc.edu/goldenPath/hg19/encodeDCC/wgEncodeBroadHmm/]

[B112] Simonet WS Bucay N Lauer SJ Taylor JM A far-downstream hepatocyte-specific control region directs expression of the linked human apolipoprotein E and C-I genes in transgenic mice J Biol Chem 1993 14 8221 8229 7681840

