# Supplementary material for: Sequence signatures extracted from proximal promoters can be used to predict distal enhancers
Source: Genome Biol. 2013 Oct 24;14(10):R117. doi: 10.1186/gb-2013-14-10-r117 (PMC3983659; doi:10.1186/gb-2013-14-10-r117)
Supplement: Additional file 1 — Figures S1 to 15 and Supplementary notes. [file gb-2013-14-10-r117-S1.pdf]

Additional file 1 for

Sequence signatures extracted from proximal promoters can be used to predict distal enhancers

Leila Taher[1], Robin P. Smith[2], Mee J. Kim[2], Nadav Ahituv[2,*] and Ivan Ovcharenko[1,*]

[1]Computational Biology Branch, National Center for Biotechnology Information, National Library of Medicine, National Institutes of Health, Bethesda, Maryland 20894, United States of America

[2]Department of Bioengineering and Therapeutic Sciences, Institute for Human Genetics, University of California San Francisco, San Francisco, California 94143, United States of America

[*] To whom correspondence should be addressed: ovcharen@nih.gov (Ivan Ovcharenko) and Nadav.Ahituv@ucsf.edu (Nadav Ahituv).

Supplementary Figures

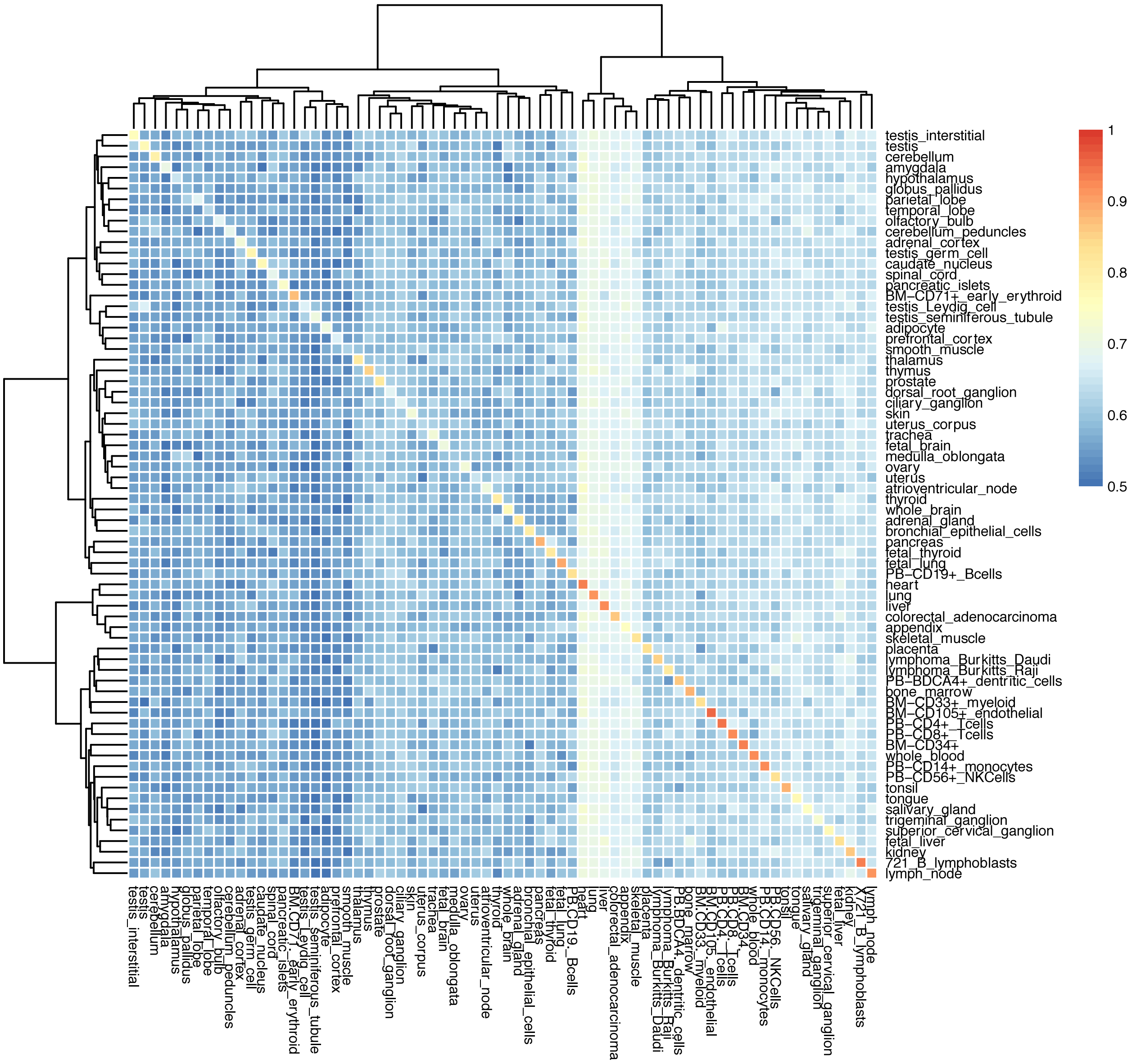

Figure S1. AUC of promoter-based classifiers (on the right) trained on specific tissues and tested on all tissues (at the top). The diagonal corresponds to classifiers trained and tested on the same tissues. Only models for which we obtained a reliable classification $AUC \geq .$ are depicted. For the heatmap, columns were clustered using Euclidean distance and average. Promoters shared between two tissues were excluded from the test set.

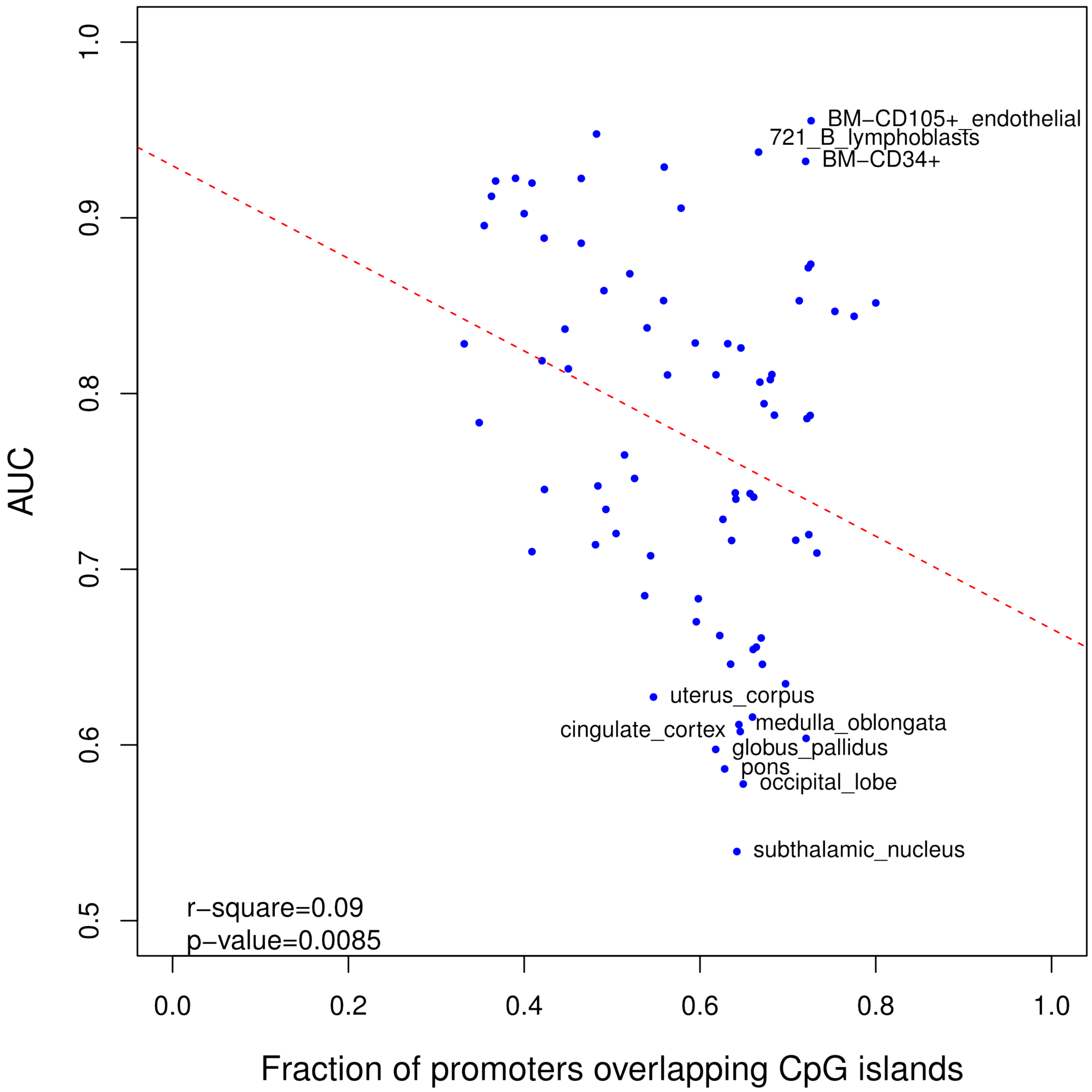

Figure S2. AUC accuracy of promoter-based models evaluated using a 5-fold cross-validation as a function of the fraction of the promoters of the 200 most highly expressed genes in a given tissue that were used for training the models that overlap CpG islands. Only 10 outliers are labeled.

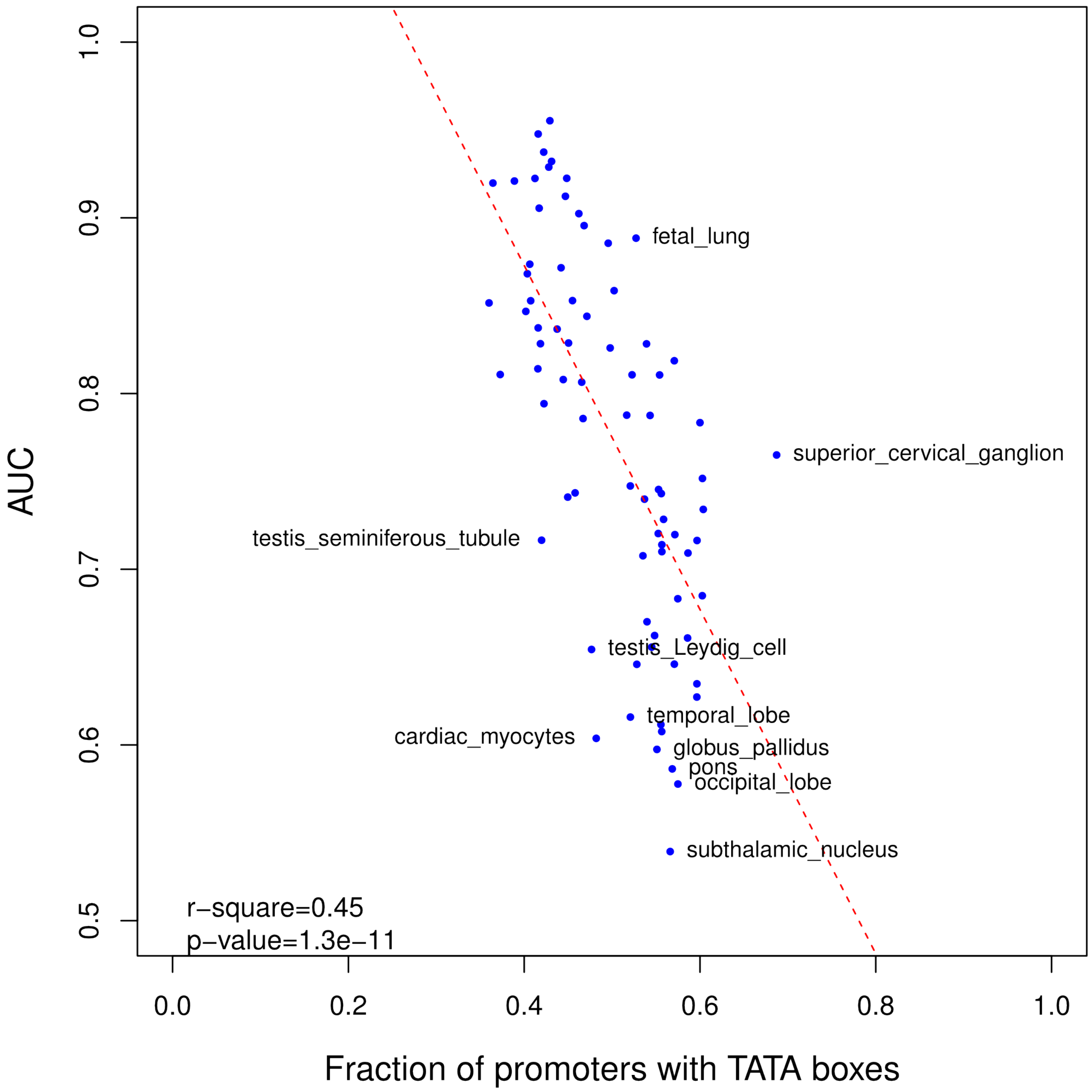

Figure S3. AUC accuracy of promoter-based models evaluated using a 5-fold cross-validation as a function of the fraction of the promoters of the 200 most highly expressed genes in a given tissue that were used for training the models that contain TATA box motifs. Only 10 outliers are labeled.

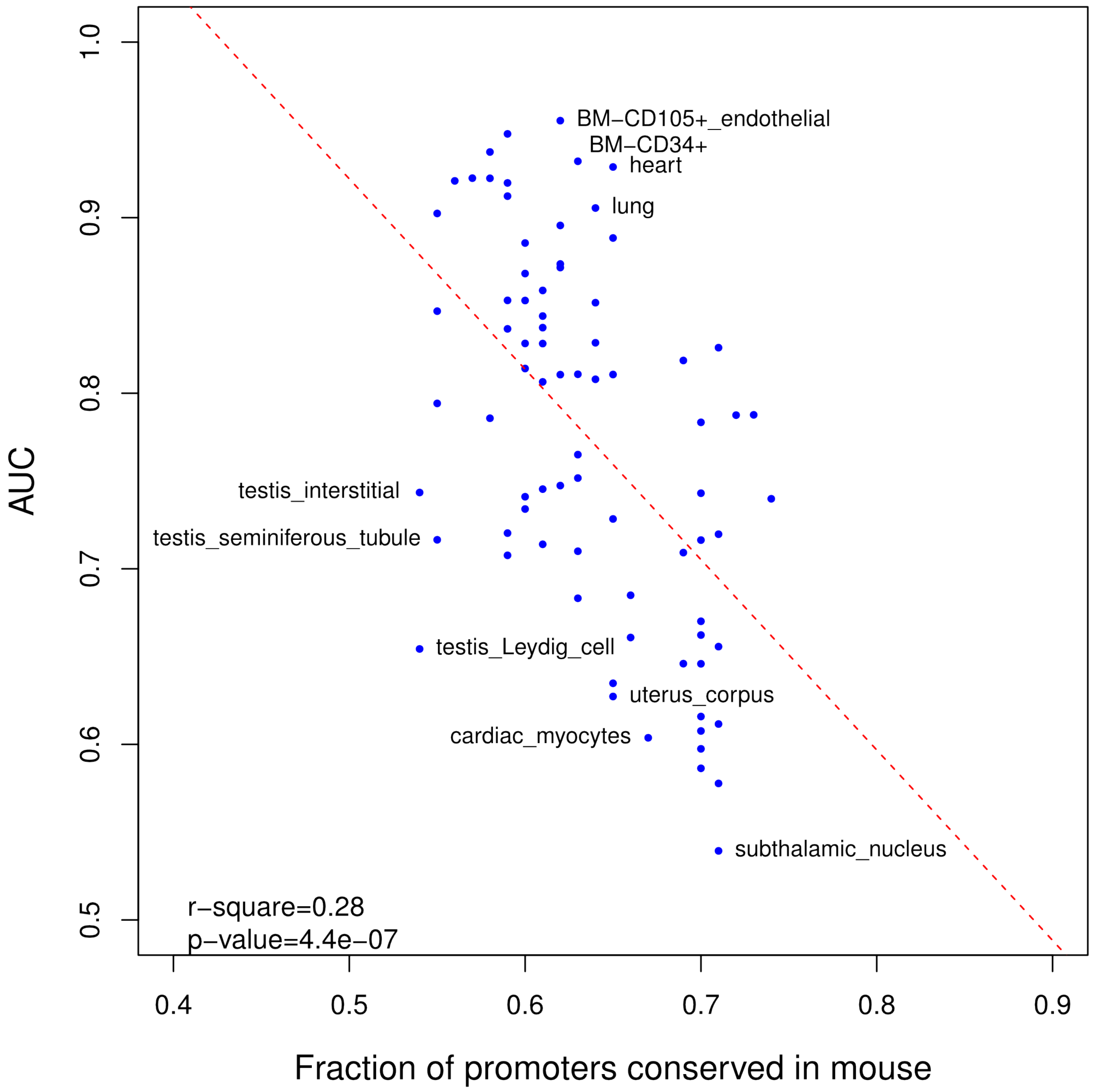

Figure S4. AUC accuracy of promoter-based models evaluated using a 5-fold cross-validation as a function of the average percent of sequence identity between human and mouse within a 50bp-window around the TSS of 200 most highly expressed genes in a given tissue. Only 10 outliers are labeled.

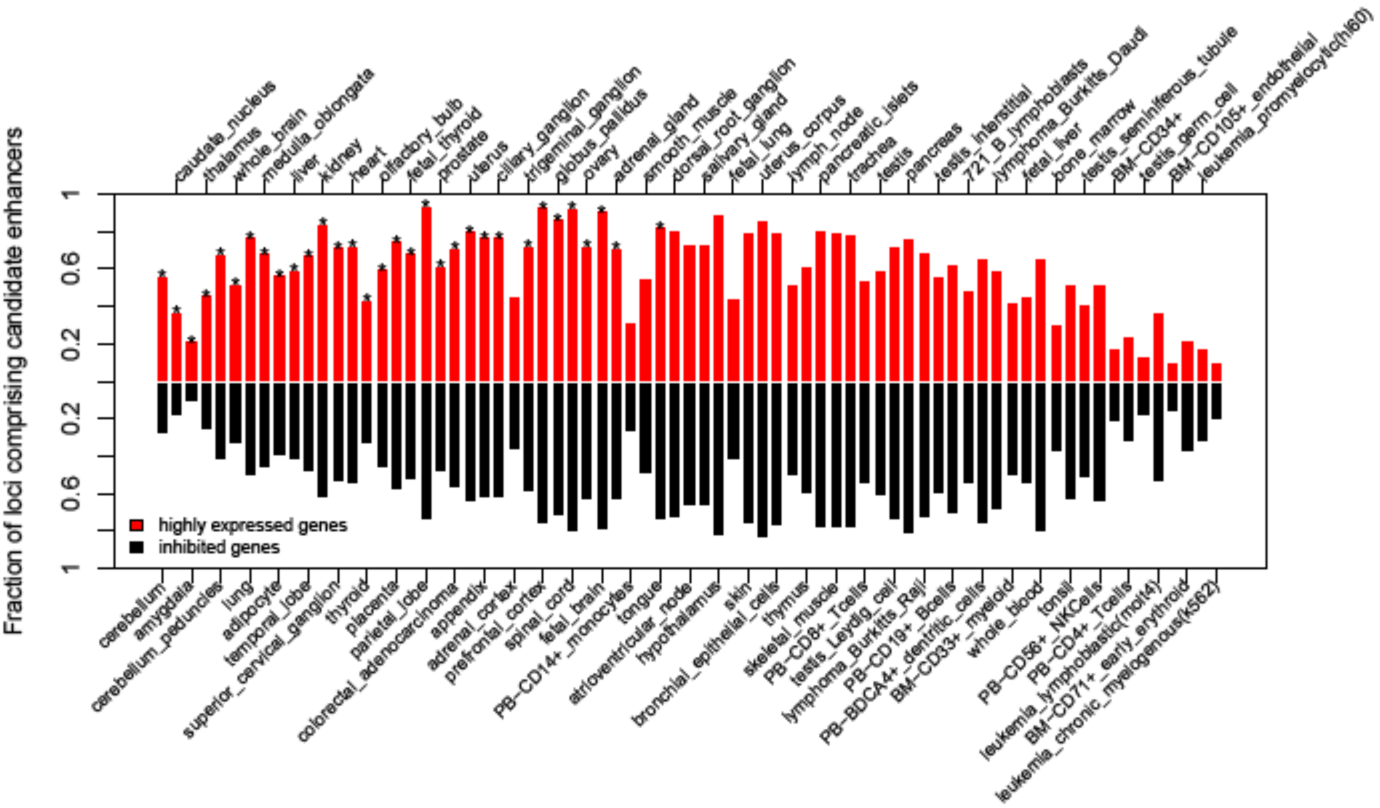

Figure S5. Number of loci in which at least one of the scanned sequences was considered a tissue-specific enhancer prediction divided by the total number of loci to which we applied the classifier, for each of the 71 tissues for which we obtained reliable promoter-based models, and both loci of highly expressed (in red) and inhibited (in black) genes.

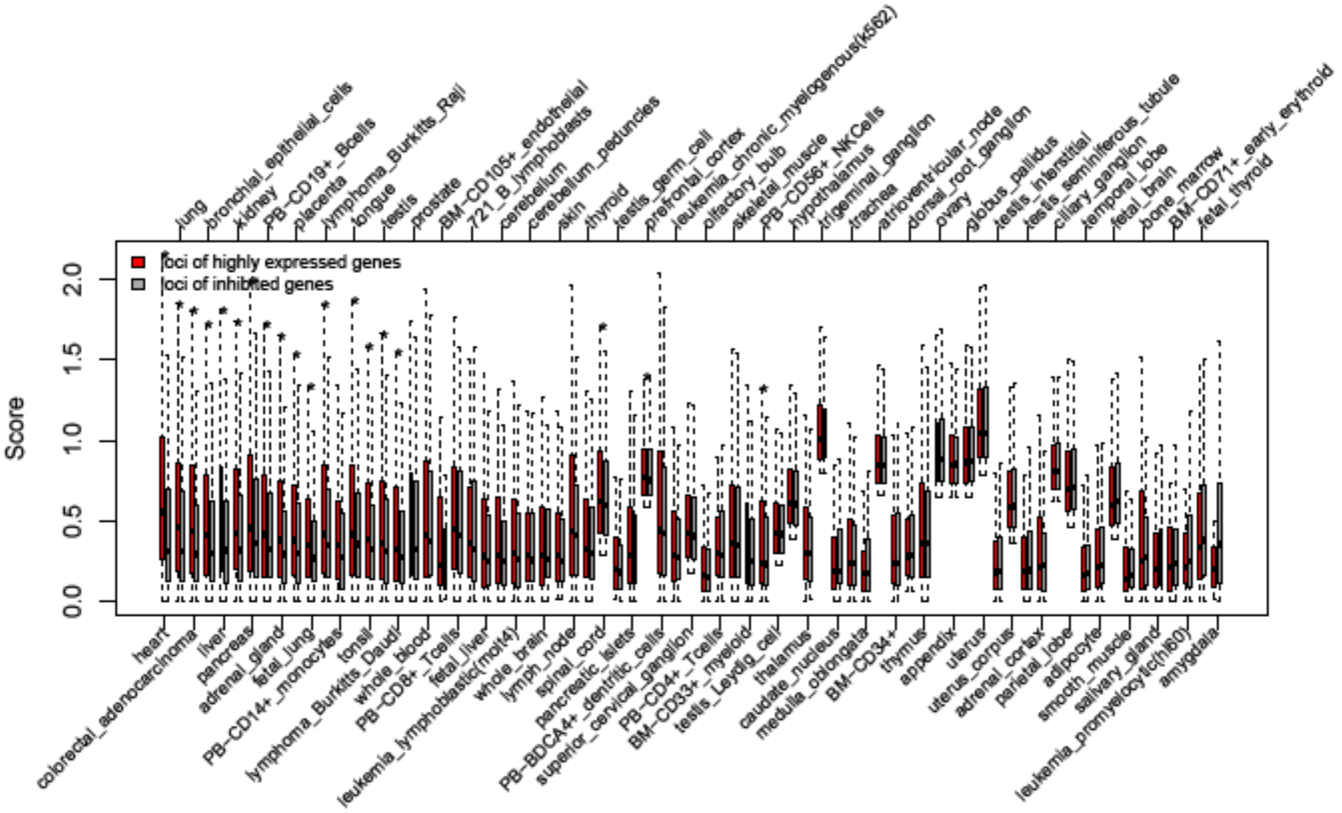

Figure S6. Boxplots showing median and interquartile range for the scores of enhancer predictions in loci of highly expressed (in red) and inhibited (in gray) genes for each of the 71 tissues for which we obtained reliable promoter-based models.

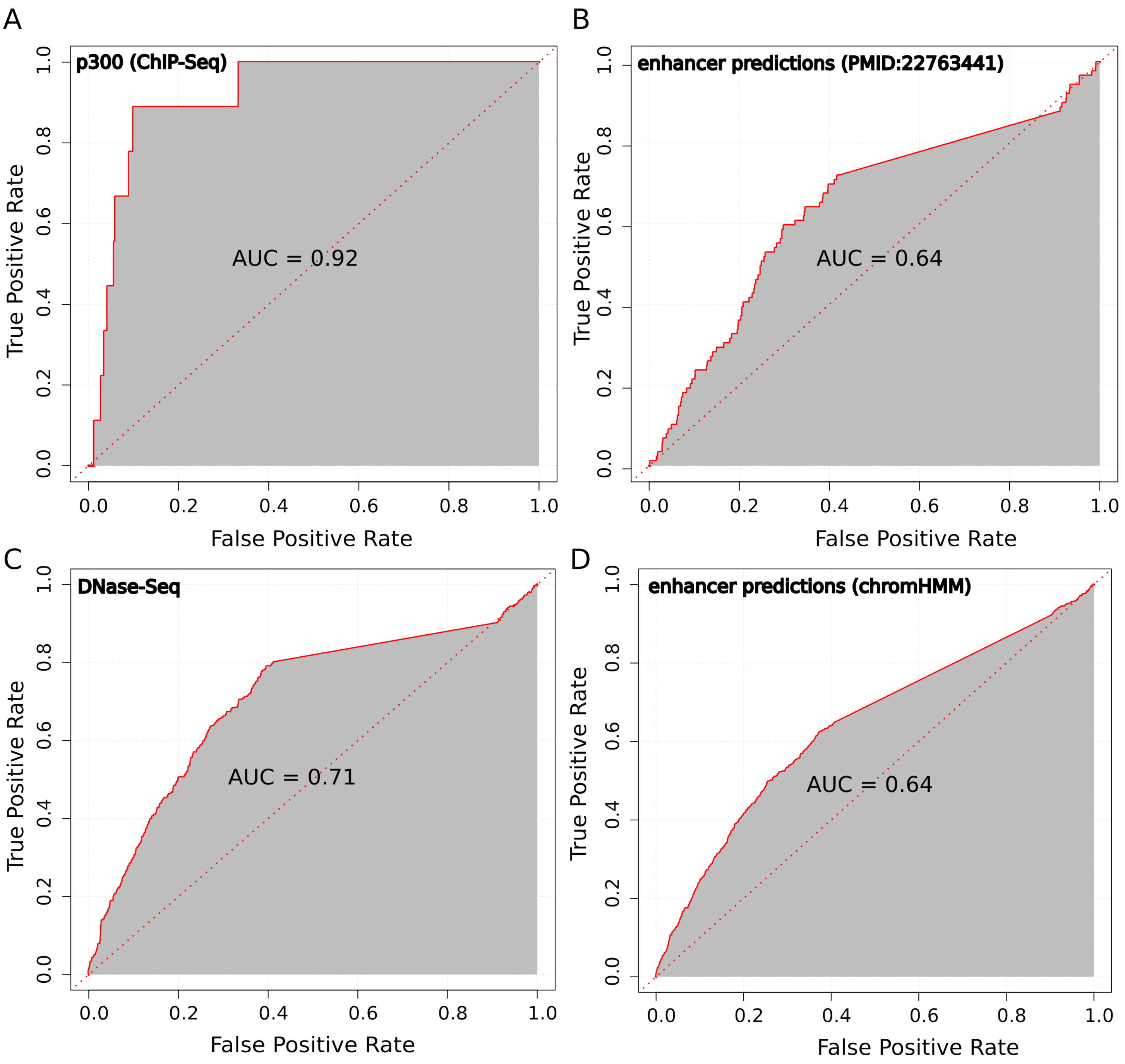

Figure S7. Receiver operating characteristic (ROC) curve evaluating the performance of the liver classifier at predicting conserved liver enhancers in loci of highly expressed genes. Predictions were compared with four different enhancer markers. A) ChIP-seq on p300 in adult mouse liver [1]. B) Enhancers predicted on the basis of chromatin modification marks and p300 [2]. C) Genome-wide DNase hypersensitivity analysis (DNase-Seq) in mouse liver [3] D) Strong enhancers predicted in HepG2 cells using chromHMM [4]. Coordinates in the mouse genome were mapped to the human genome using the LiftOver tool [5].

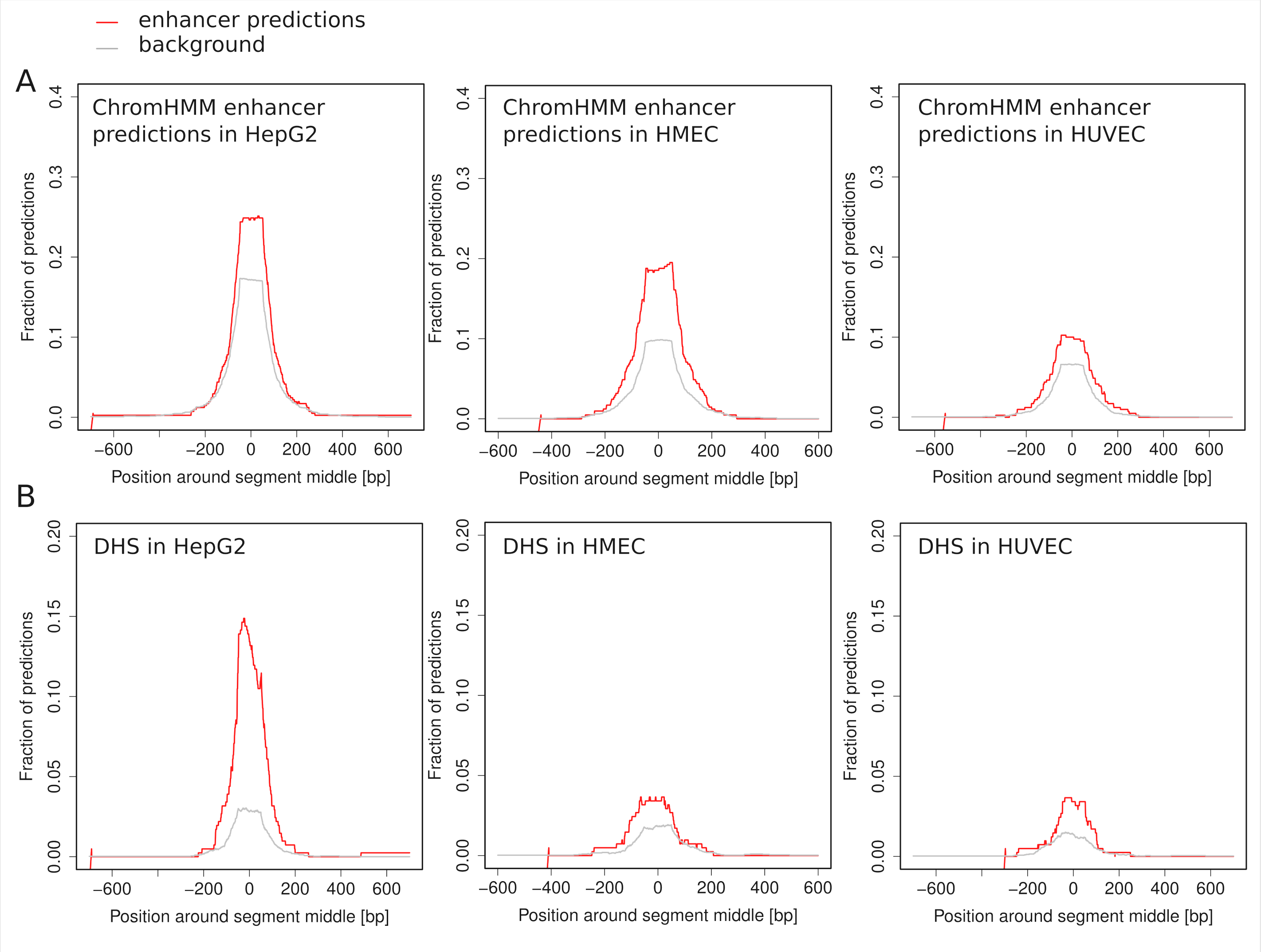

Figure S8. (A) Overlap of liver enhancer predictions with strong enhancers predicted by ChromHMM in in HepG2 cell lines [4], compared to random sequences with similar length in the loci of genes highly expressed in liver. For reference, we have included overlaps in HUVEC and HMEC cell lines. (B) Overlap of liver enhancer predictions with DNase I hypersensitivity sites (DHS) in HepG2 cell lines from the ENCODE project [6], compared to random sequences with similar length in the loci of genes highly expressed in liver. For reference, we have included overlaps in HUVEC and HMEC cell lines.

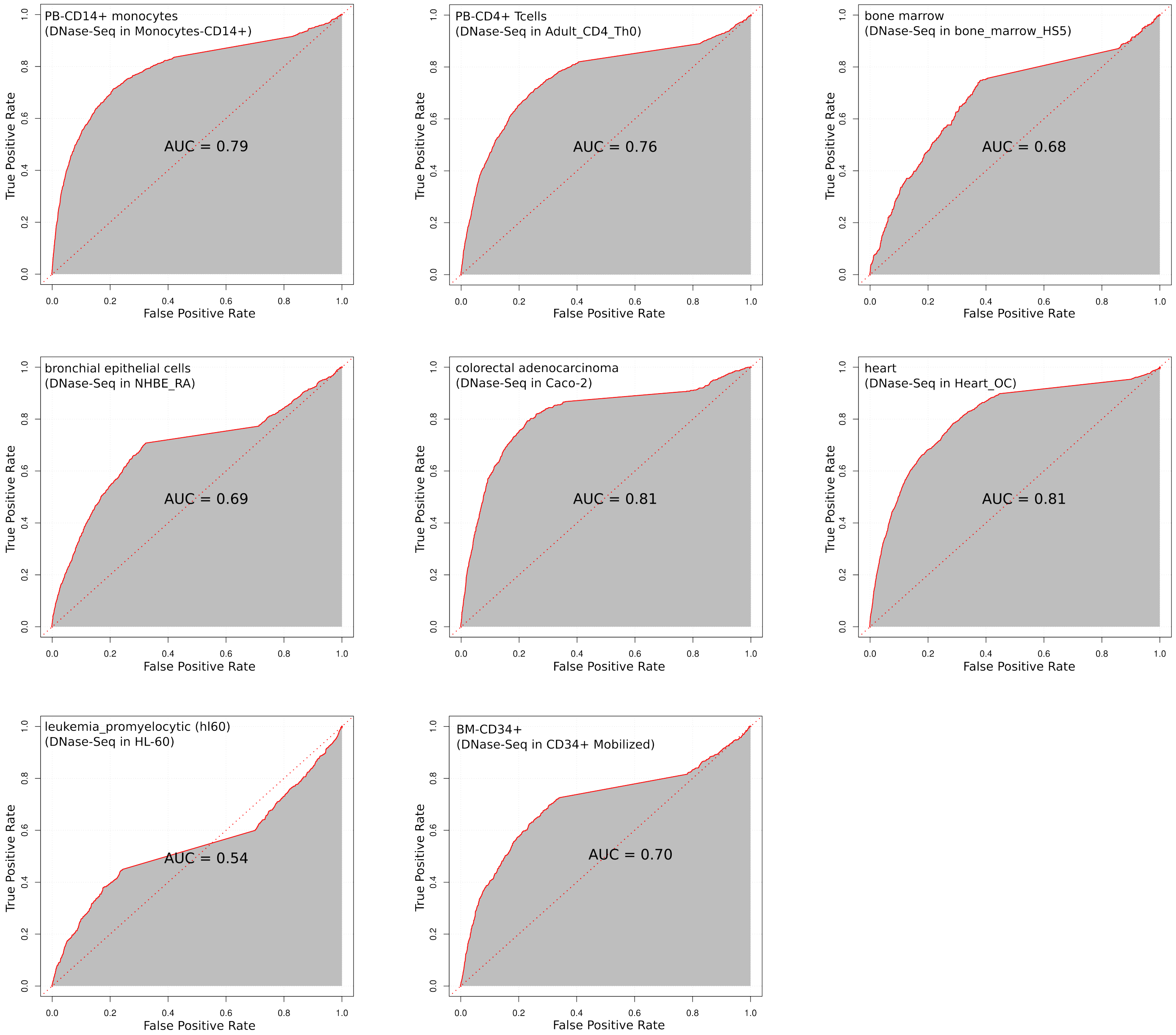

Figure S9. Receiver operating characteristic (ROC) curve evaluating the performance of the 8 classifier at predicting conserved enhancers in loci of highly expressed genes. Predictions were compared with DNase hypersensitivity data (DNase-Seq) from ENCODE for similar tissues/cell lines, as indicated between parentheses. The performance is only poor for leukemia promyelocytic cells.

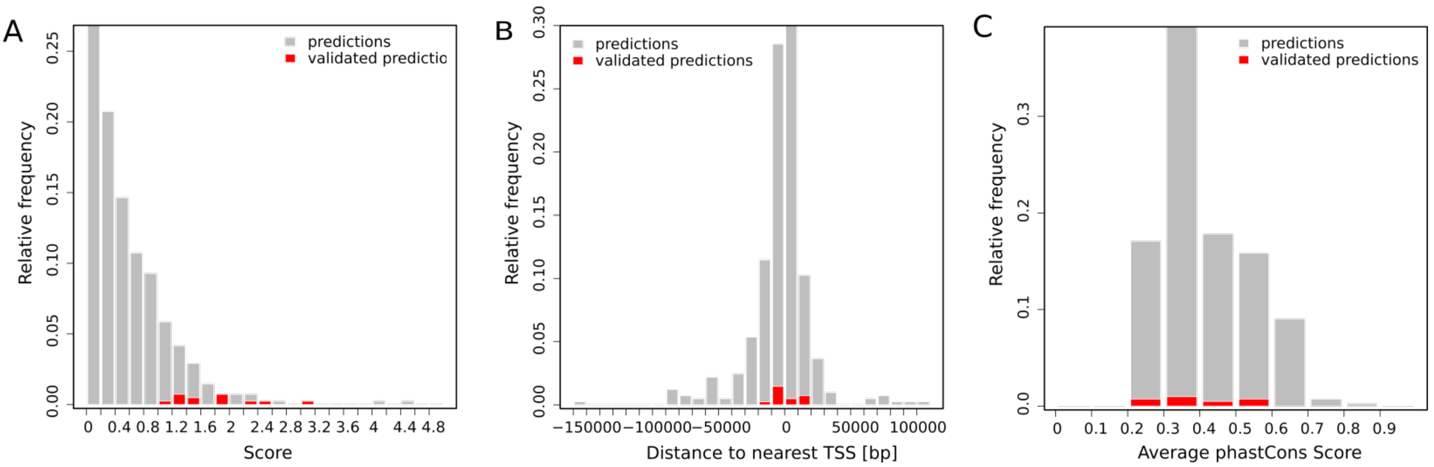

Figure S10. Experimental validation of liver enhancer predictions. (A) Score assigned by the liver promoter-based model to candidate liver enhancers (grey), including those validated experimentally (the corresponding fraction of the total is indicated in red). (B) Distance to the nearest TSS for candidate liver enhancers (grey), including those validated experimentally (red). (C) Average phastCons score for candidate liver enhancers (grey), including those validated experimentally (red).

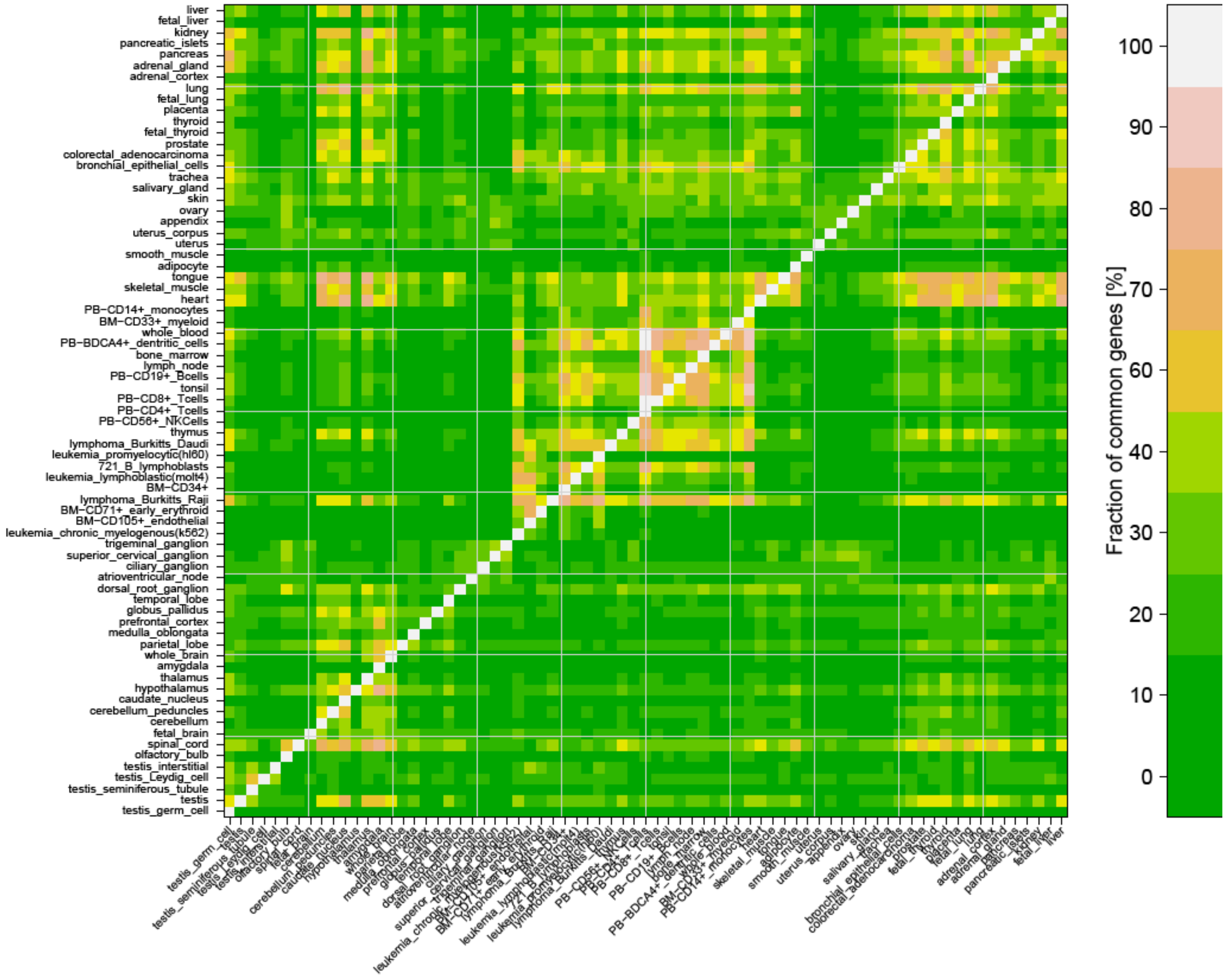

Figure S11. Fraction of genome-wide tissue-specific enhancer predictions overlapping between any two tissues. Tissues were sorted according to the similarity among the top 200 most highly expressed genes between any two tissues, using Pearson correlation and complete linkage.

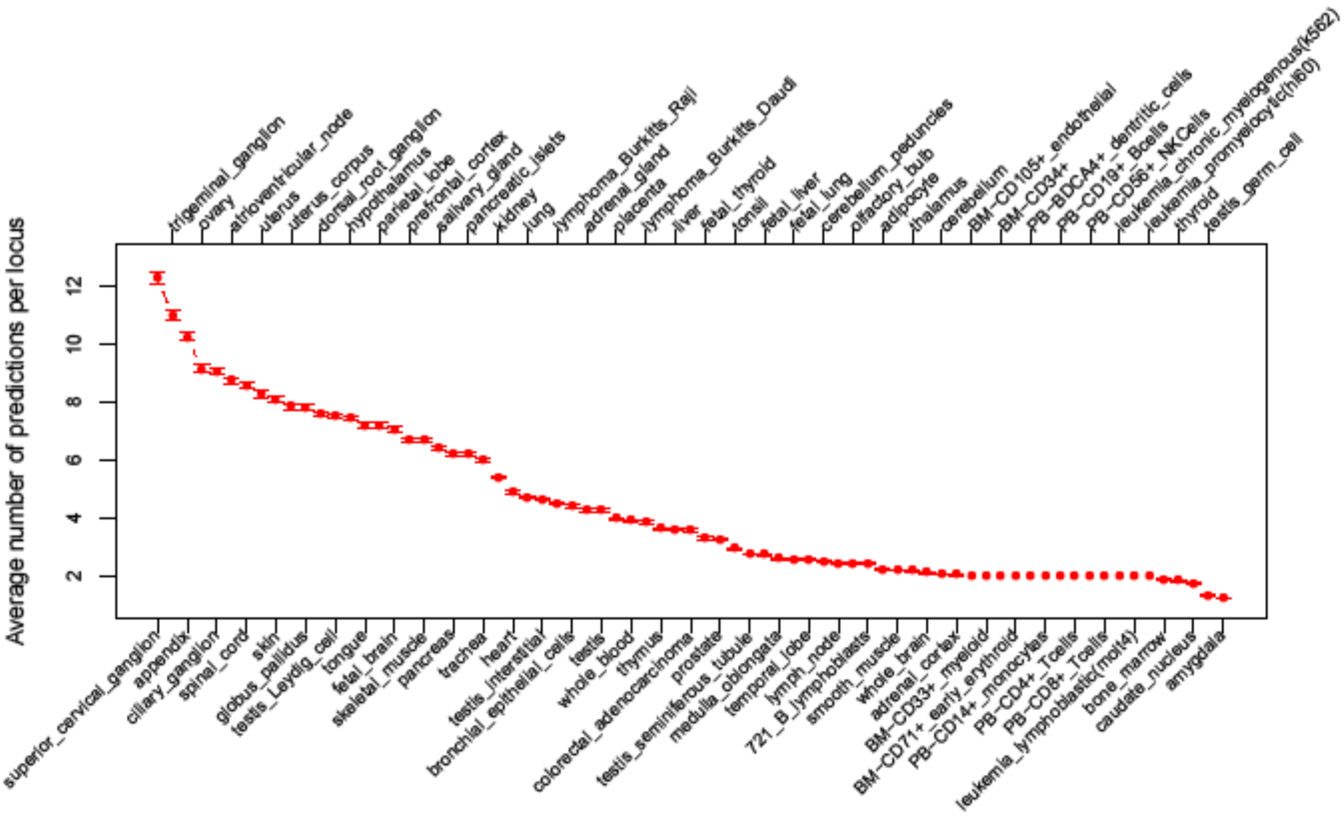

Figure S12. Average number of genome-wide tissue-specific enhancer predictions.

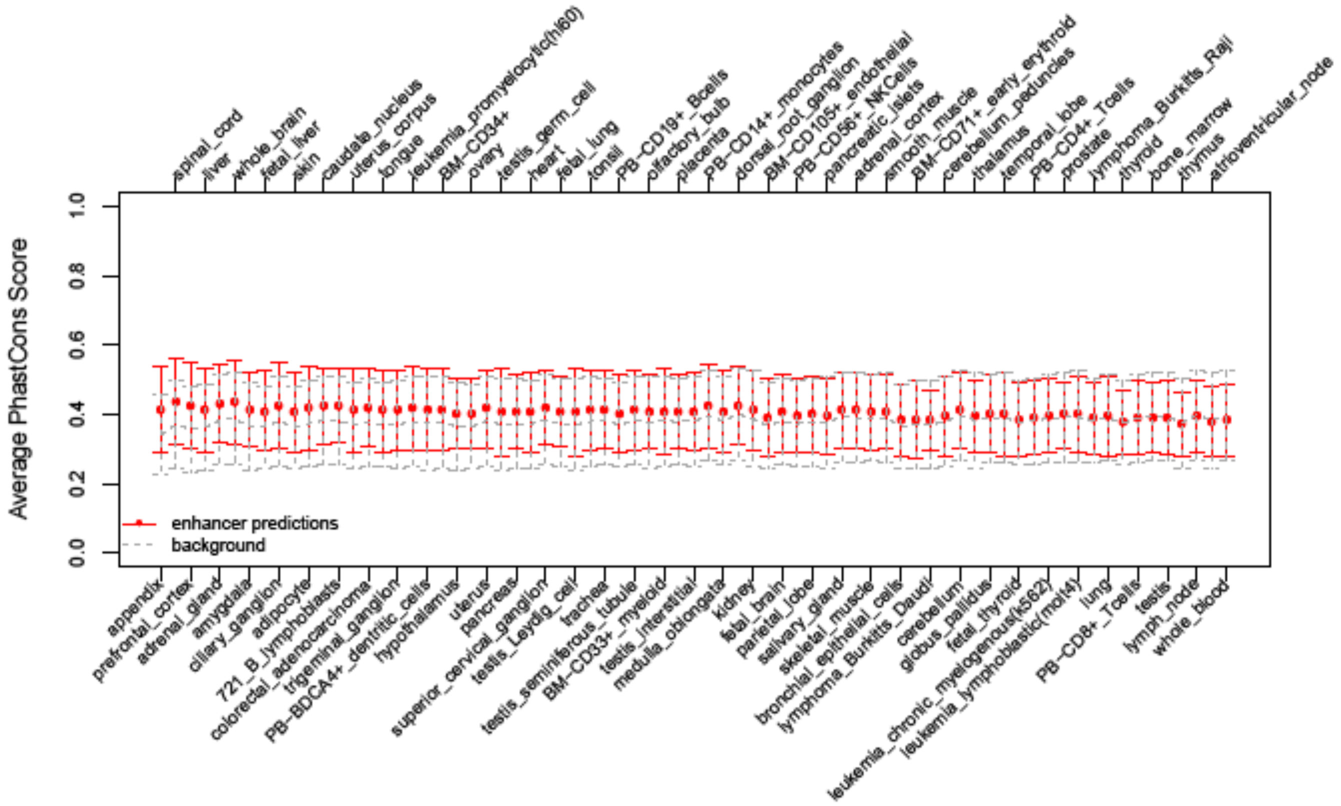

Figure S13. Average PhastCons score and associated standard error for enhancer predictions (in red), compared to randomly selected conserved noncoding elements (CNEs) with at least 70% identity across the human and mouse genomes in loci of highly expressed genes.

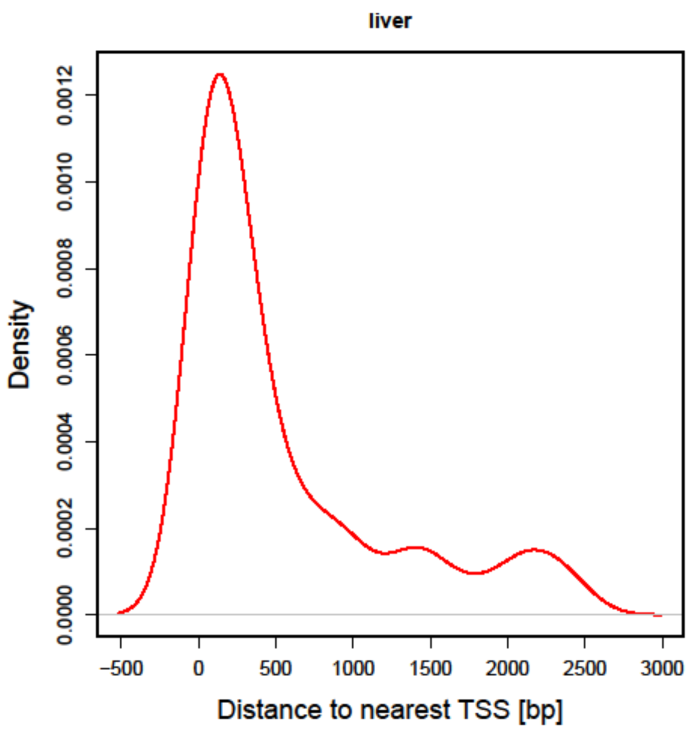

Figure S14. Distribution of conserved noncoding elements (CNEs) within the promoter regions of the 200 most highly expressed genes in liver that were used to train the promoter-based liver model for enhancer prediction (see Methods).

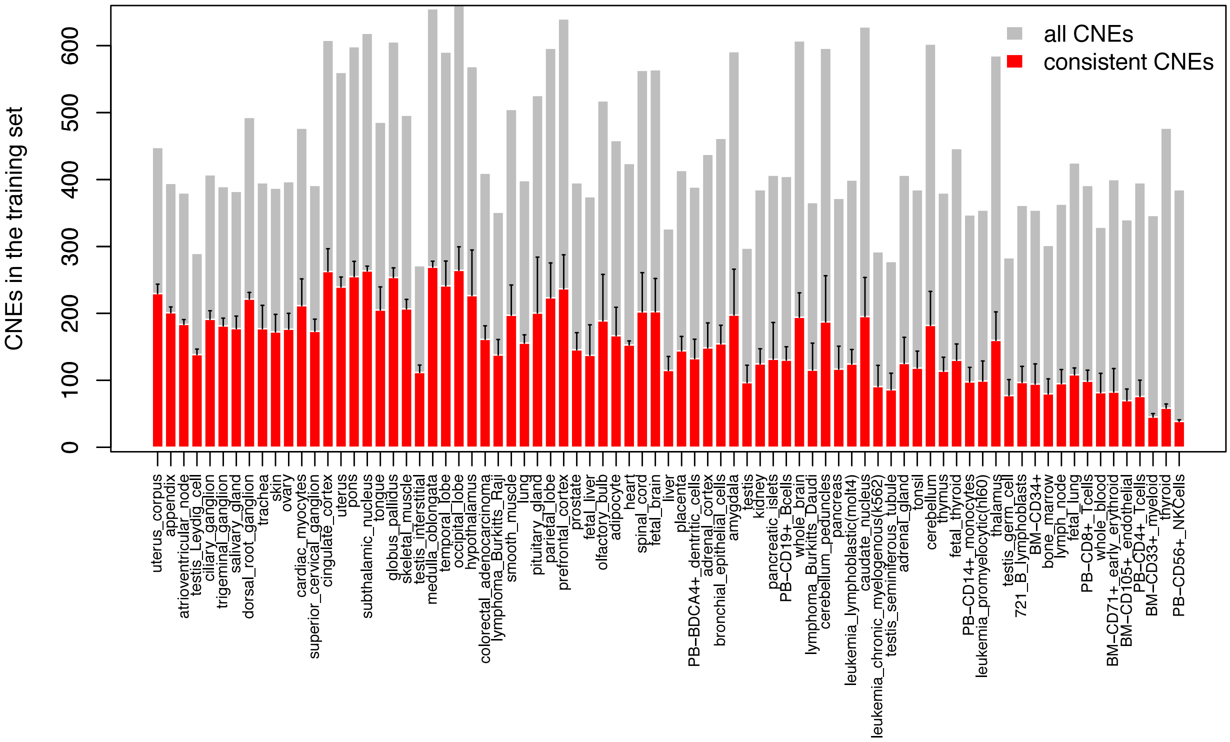

Figure S15. Number of CNEs in the consistent positive sets for each of the 79 tissues considered, computed in the cross-validation framework.

Supplementary Notes

Promoter-based models are robust to changes in the number of promoters in the training set

To assess to which degree our models describing tissue-specific promoter activity could be exploited to discover enhancers, we applied each of the 73 reliable models trained on promoter regions to predict enhancers in the loci of genes that were among the 200 most highly or lowly expressed in the corresponding tissue (see Methods). Although this cut-off of 200 promoters is admittedly somewhat arbitrary, our results are relatively robust to this choice. A comparison of the predictions of liver classifiers trained with the promoters of the top 200, 400, 600, and 1000 most highly and lowly expressed genes in the loci of the 200 most highly expressed genes for liver is shown below. The classifier trained on 200 promoters produces a slightly larger number of predictions than the others, but, in general, there is approximately a ~75% overlap (see Figure I).

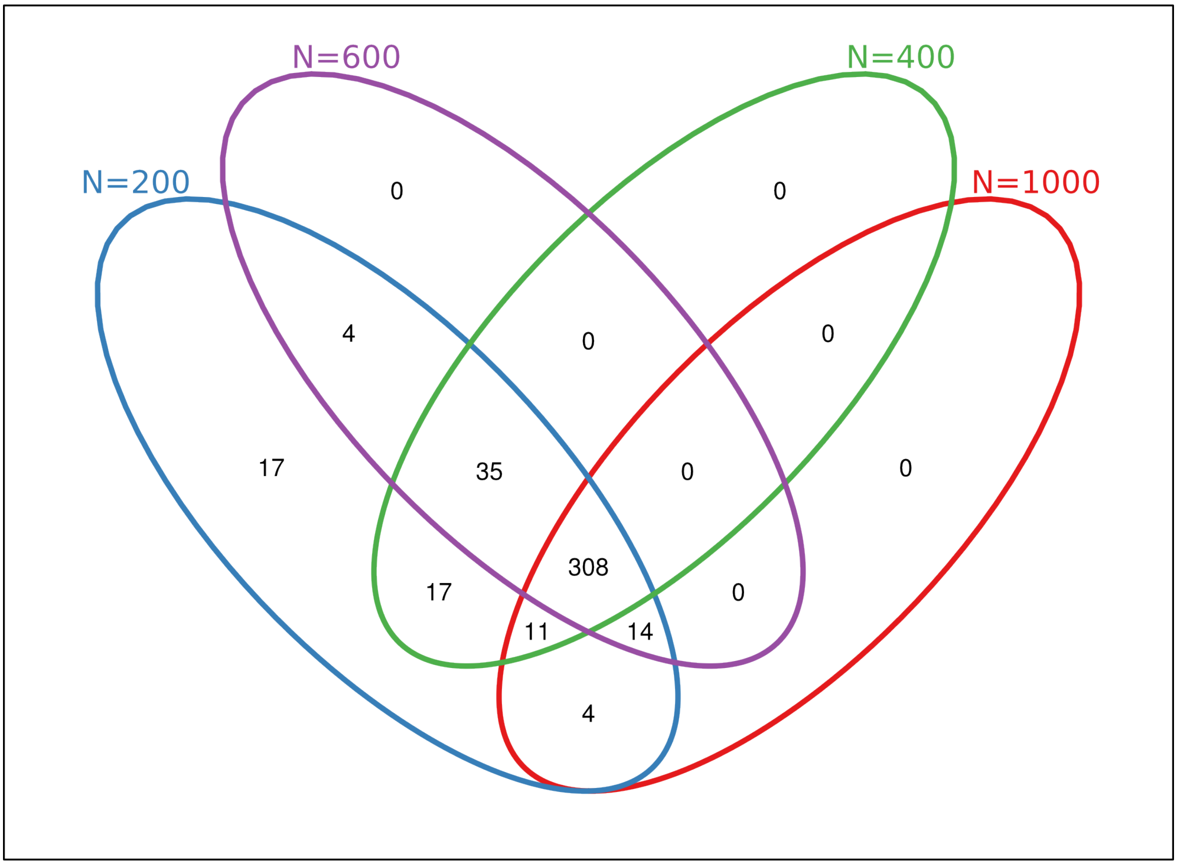

Figure I. Overlap of conserved liver enhancer predictions in loci of the 200 most highly expressed genes in liver, computed by classifiers trained on different numbers N of promoters of most highly and lowly expressed genes. For example, N=1000 indicates a model that was trained on 1000 promoters of most highly expressed and 1000 promoters of most lowly expressed genes in liver.

Sets of lowly expressed genes are tissue-specific

Depending on the tissues involved in the analysis, specific gene families with very restricted expression domains may be over-represented among sets of most lowly expressed genes. However, we did not observe a large overlap between the sets of most lowly expressed genes chosen for each tissue. Between any pair of tissues we found an overlap of 7%, as reflected in Figure II. Also clusters computed on pairwise

overlaps reflect relationships between the tissues. For these clusters we found pairwise overlaps close to 40%.

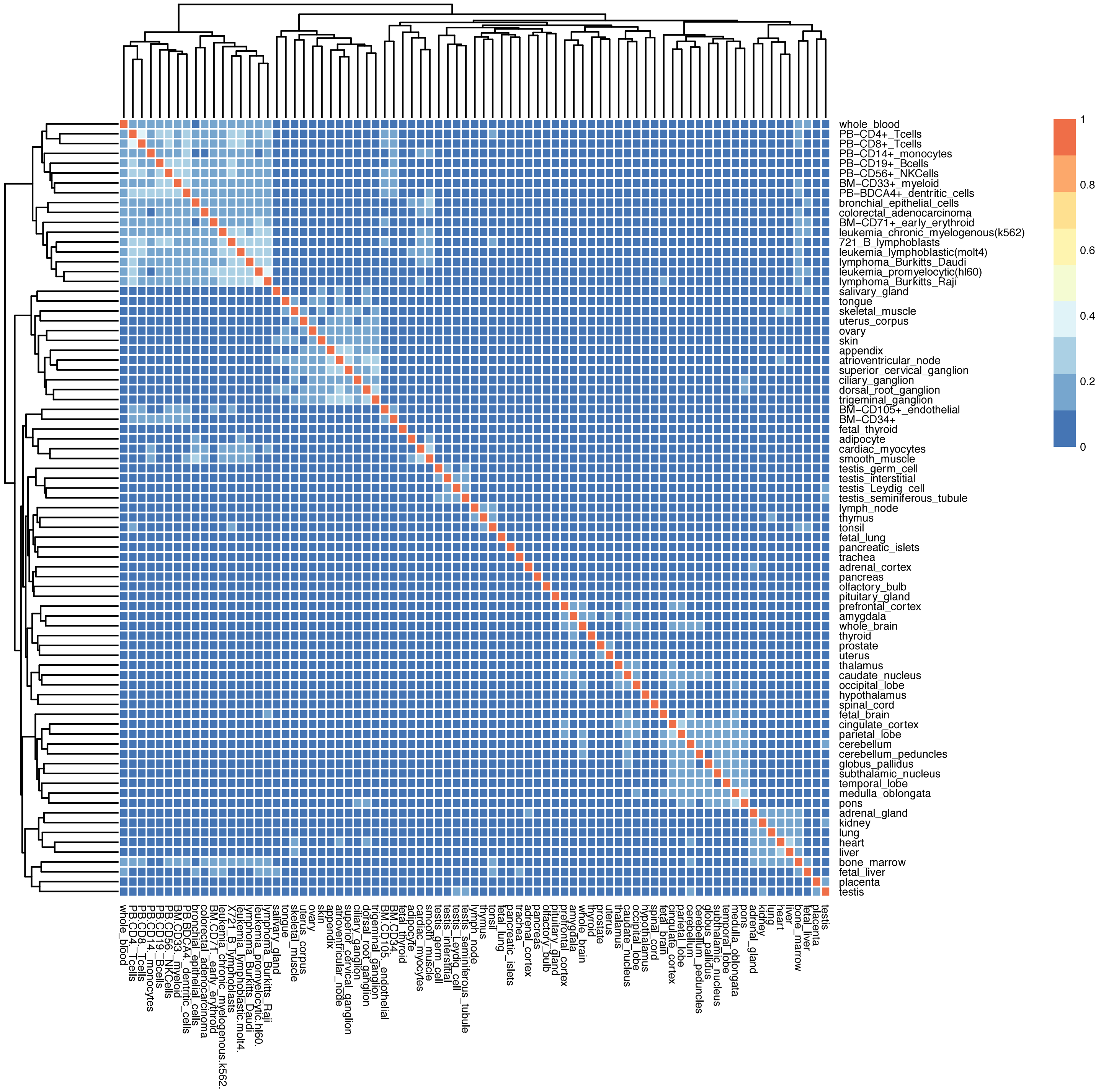

Figure II . Fraction of overlap between tissue-specific sets of lowly expressed genes. Fractions of genes overlapping between the sets of most lowly expressed genes chosen for any two tissues are clustered using Euclidean distance and average linkage.

Highly expressed genes in each of the 79 tissues considered exhibit distinct structural and genomic characteristics

In contrast to housekeeping genes, which are ubiquitously expressed and perform basic cellular functions, tissue-specific genes are selectively expressed at a usually much higher level in a few tissues only. Little is known

about the genomic features and mechanisms responsible for both basal and tissue-specific expression. For this reason, we first examined the differences between the 200 most and least highly expressed genes in each of 79 human tissues whose transcriptomes are available in the GNF Gene Expression Atlas 2 [7]. We found that for most tissues, the median of the expression value of the most highly expressed genes in a given tissue is not only significantly higher than the expression of an average gene (log ratios 2.1 to 5.2, Figure III), but also with respect to the median of the expression value in the remaining tissues (log ratios 2.0 to 5.0, Figure IV), suggesting that most of these genes are expressed in a rather tissue-specific manner.

Sequence analysis reveals differences among highly expressed genes in different tissues. For example, genes most highly expressed in the nervous system are relatively well conserved, with over 70% of these genes preceding the tetrapod radiation, as compared to only 60% of genes highly expressed in testis, for example (Figure V). Also, genes highly expressed in the nervous system are separated from other genes by relatively long stretches of noncoding sequence enriched with deeply conserved noncoding elements (CNEs). For example, genes highly expressed in the fetal brain and the adult brain are separated from their immediate neighboring genes by an average distance of 54 and 39 kb, respectively, while genes highly expressed in the heart are at an average distance of only 9 kb from their nearest neighbors (Figure VI). In turn, intergenic regions flanking genes highly expressed in the nervous system contain a large number of CNEs, with the average of $3.0x10^{-5}$ and $2.6x10^{-5}$ human/zebrafish CNEs per kb per intergenic region for fetal and adult brain, respectively, as compared with the global average of $5.0x10^{-6}$ for the human genome (P-values << 0.05, computed with the Wilcoxon Rank-Sum Test, Figure VII). Such evolutionary and structural peculiarities may underlie distinct mechanisms governing tissue-specific gene expression.

In summary, these results support the hypothesis that requirements for tissue-specific regulation impose selection constraints on coding and noncoding sequences, hence shaping gene structure, genomic distribution, and evolutionary conservation of tissue-specific genes and their regulatory regions. We speculate that tight tissue-specific control is achieved through specific interaction between enhancers and promoters, and thus, that particular signatures within tissue-specific promoters can be identified and used to predict active enhancers. Identifying such signatures would provide a computational, genome-wide means of identifying potential distant enhancers helping to prioritize costly experimental assays.

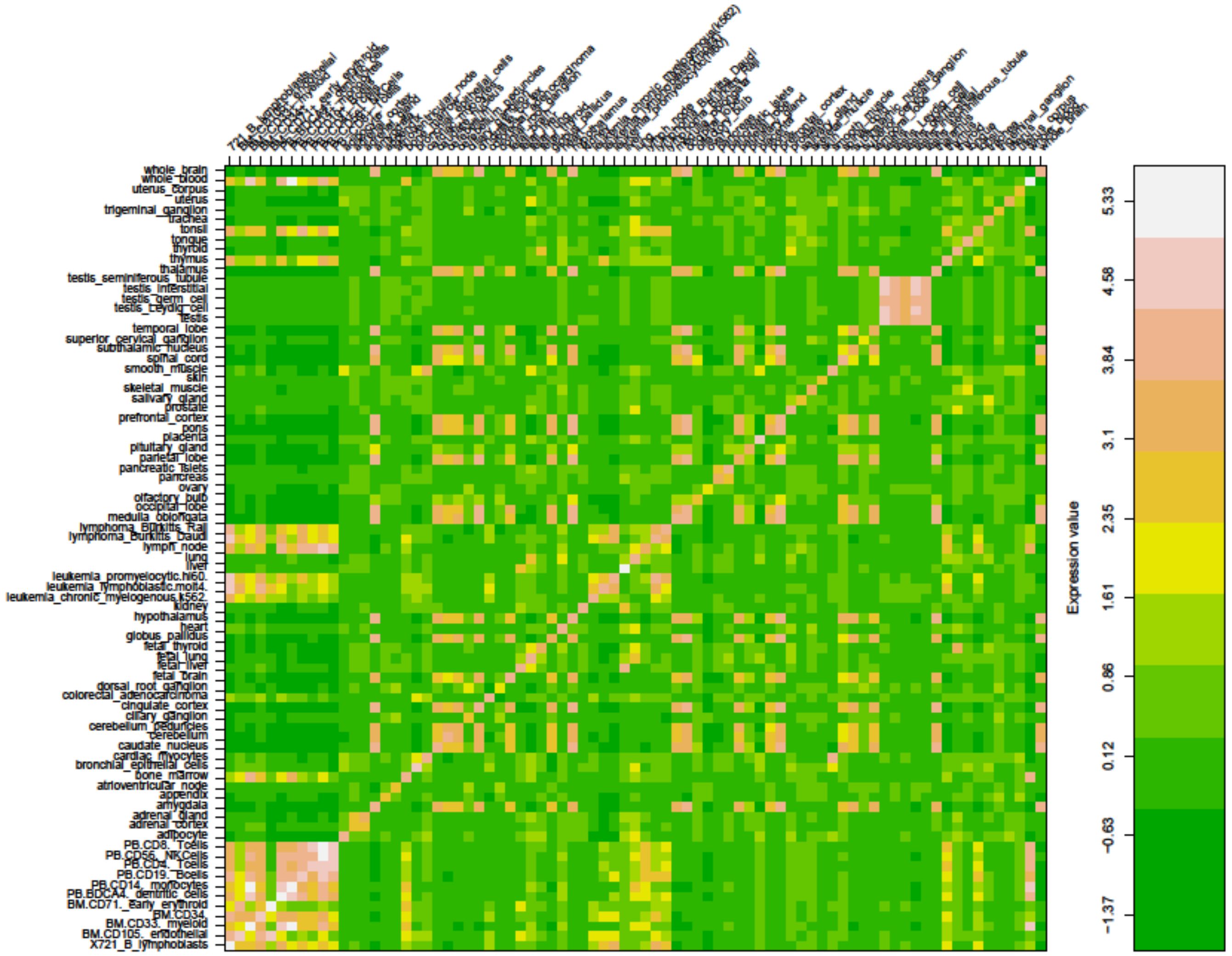

Figure III. Average expression value in each of the 79 human tissues considered for the 200 most highly expressed genes in each of the tissue.

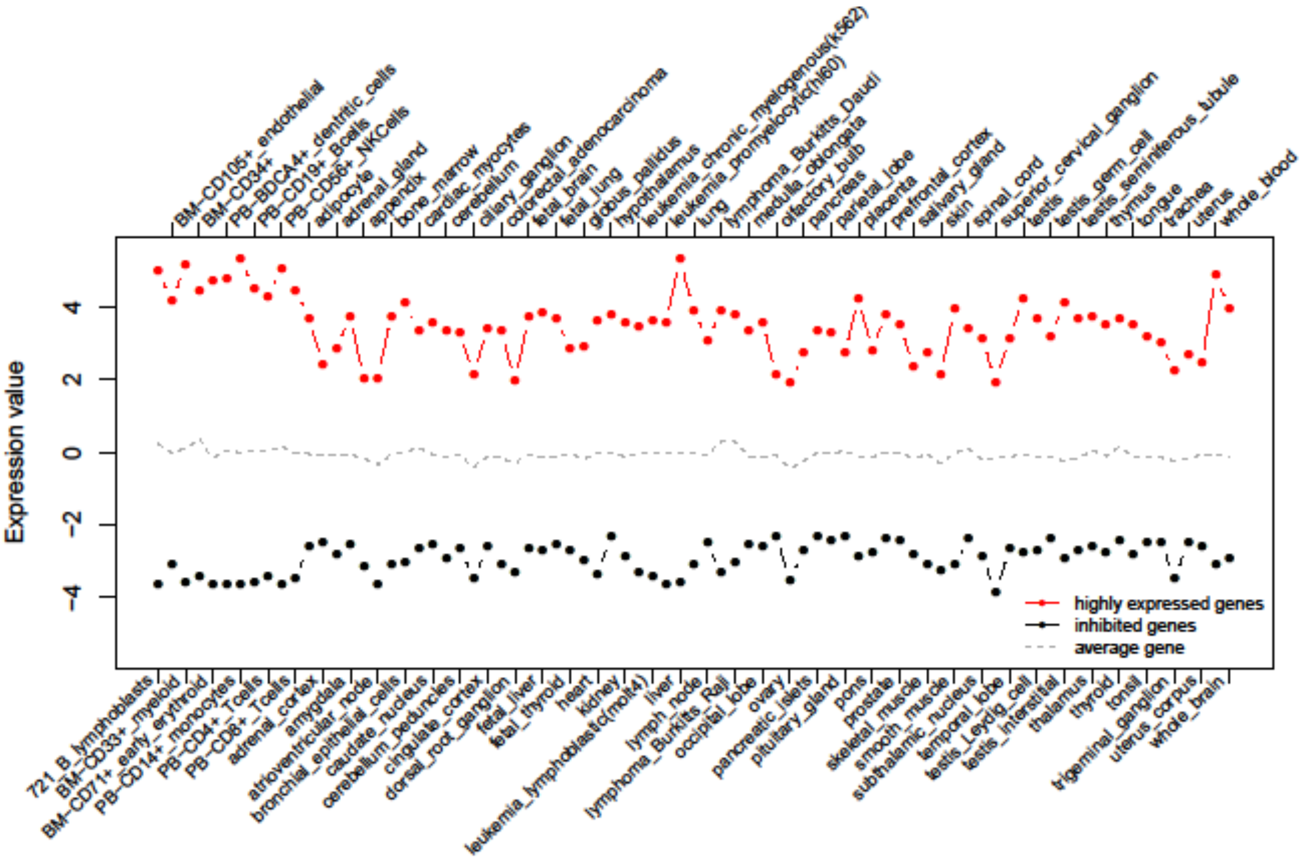

Figure IV. Median of the expression value for the 200 most highly expressed (in red) and 200 most inhibited (in black) genes in 79 human tissues. The median of the expression value of an average gene (in gray) was estimated by randomly selecting 200 genes from the human genome.

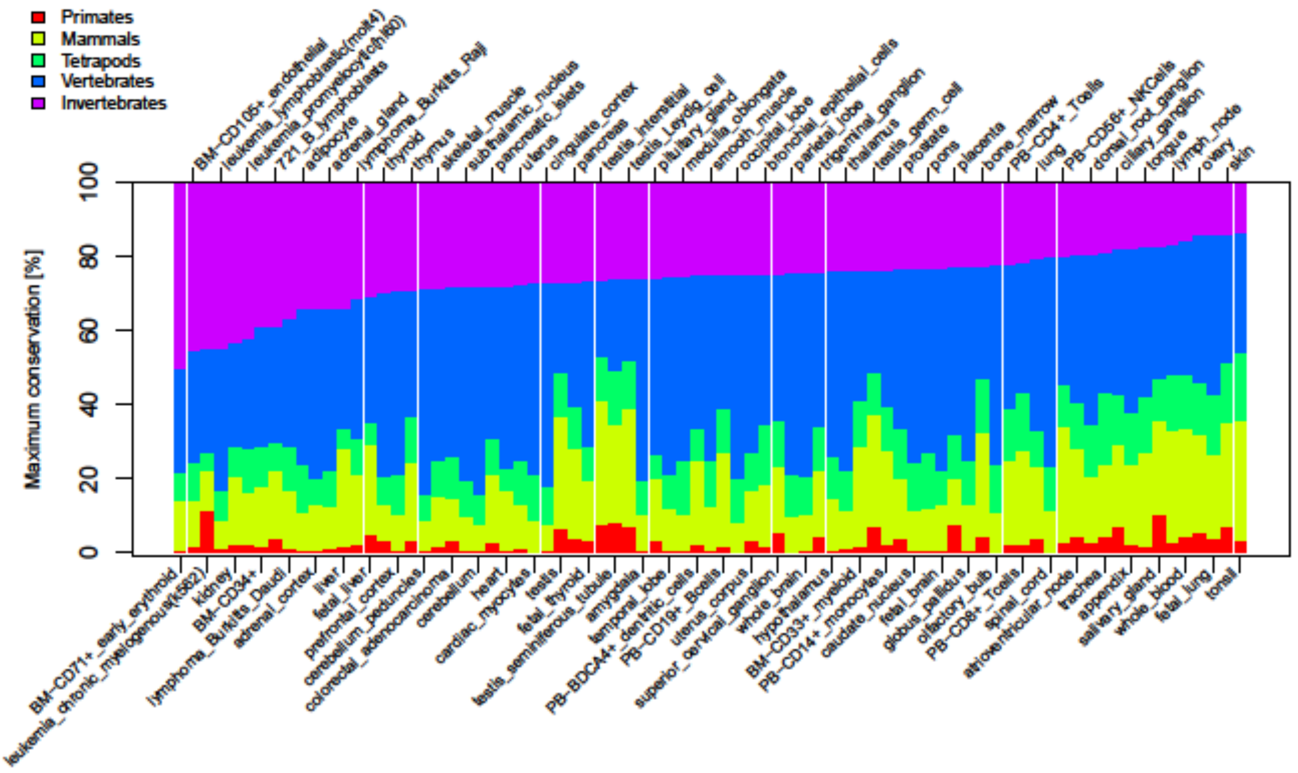

Figure V. Sequence conservation of 200 most highly expressed genes in 79 tissues. Fraction of genes conserved across different lineages.

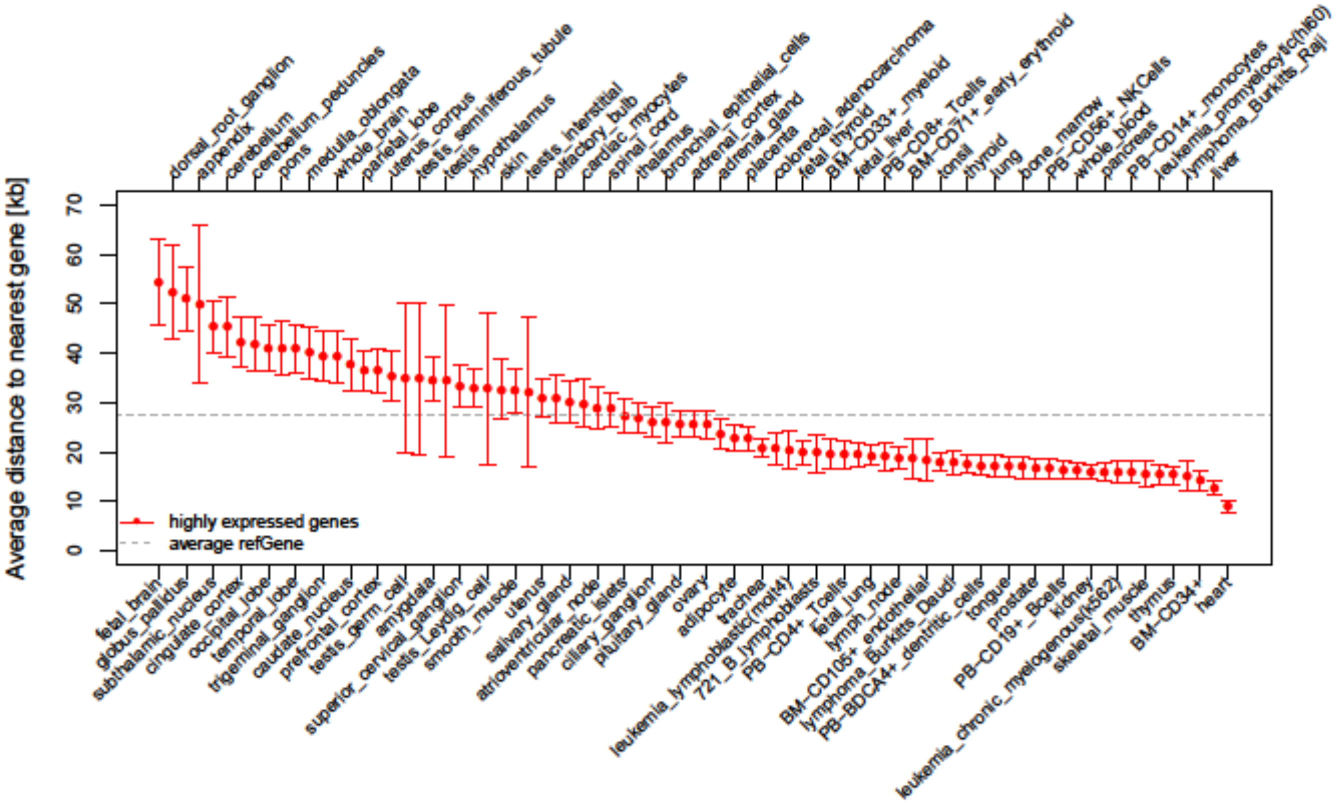

Figure VI. Average distance (in kbp) to the nearest gene (independently of its expression profile) for each of the 200 most highly expressed genes in each of 79 human tissues (in red), compared to the average distance between any two genes in the human genome (refGene, in gray).

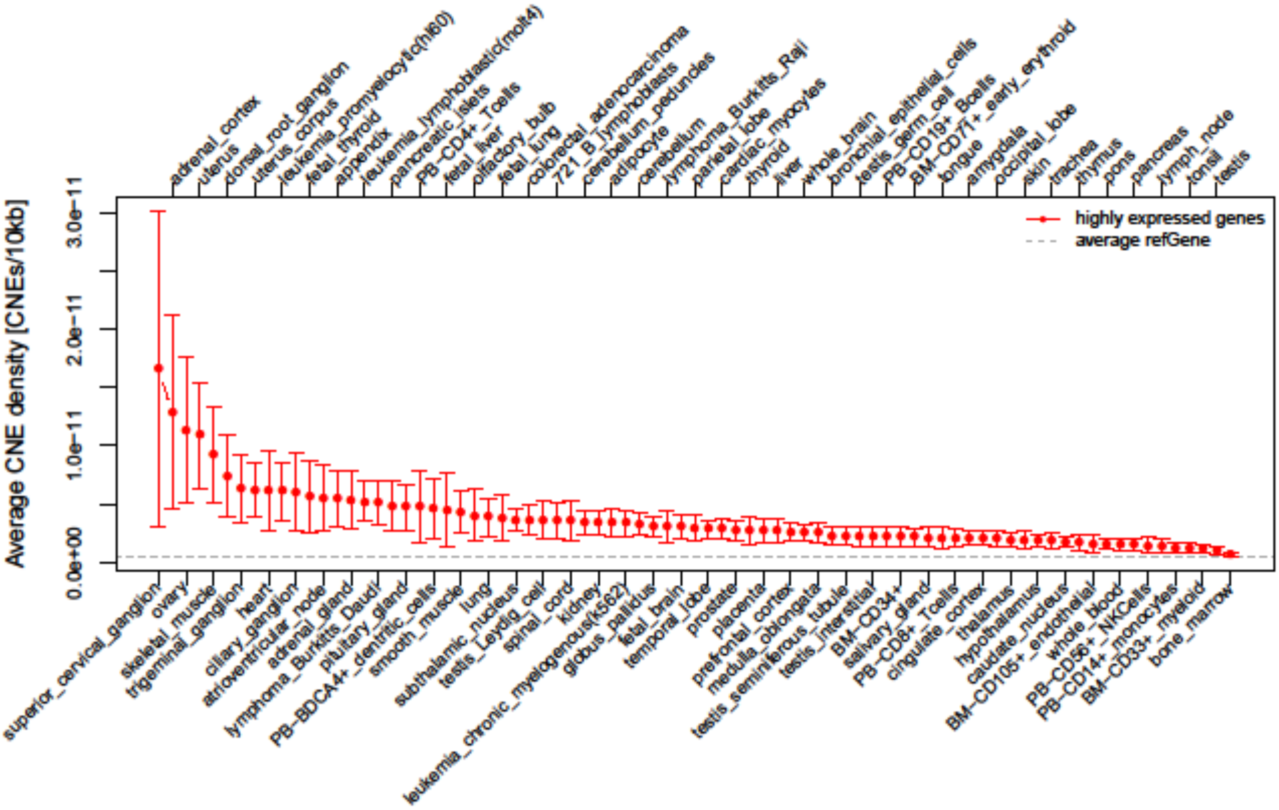

Figure VII. Number of human/zebrafish conserved noncoding elements (CNEs) per 10 kb of sequence per intergenic region, computed for the 200 most highly expressed genes in 79 tissues (in red), compared to the average observed for the genes in the human genome (refGene, in gray).

The promoters of highly expressed genes in each of the 79 tissues considered are well conserved

In general, the promoter regions of these genes are evolutionary conserved, particularly in close proximity to the TSS (with a median of 63% sequence identity between human and mouse around the TSS, as compared to 58% for an average human promoter, Figure VIII). However, the degree of conservation varies depending on the tissue where the corresponding genes are expressed. For example, genes most highly expressed in the brain are characterized by extremely conserved promoters (e.g., 74% identity as compared to mouse, in the case of cerebellum peduncles), while promoters of genes expressed in other tissues, such as testis, are less conserved (e.g., 54% identity for testis interstitial cells). Consistent with previous studies, we observed that promoter conservation is modestly but positively correlated with coding conservation (r-squared = 0.4, p-value = 2.2×10^{-9}, computed using the F-test, [8]). In addition, most promoter regions of most highly expressed genes contain at least two conserved noncoding elements [CNEs, [9], data not shown], supporting the existence of multiple discrete regulatory units in close proximity to the TSS.

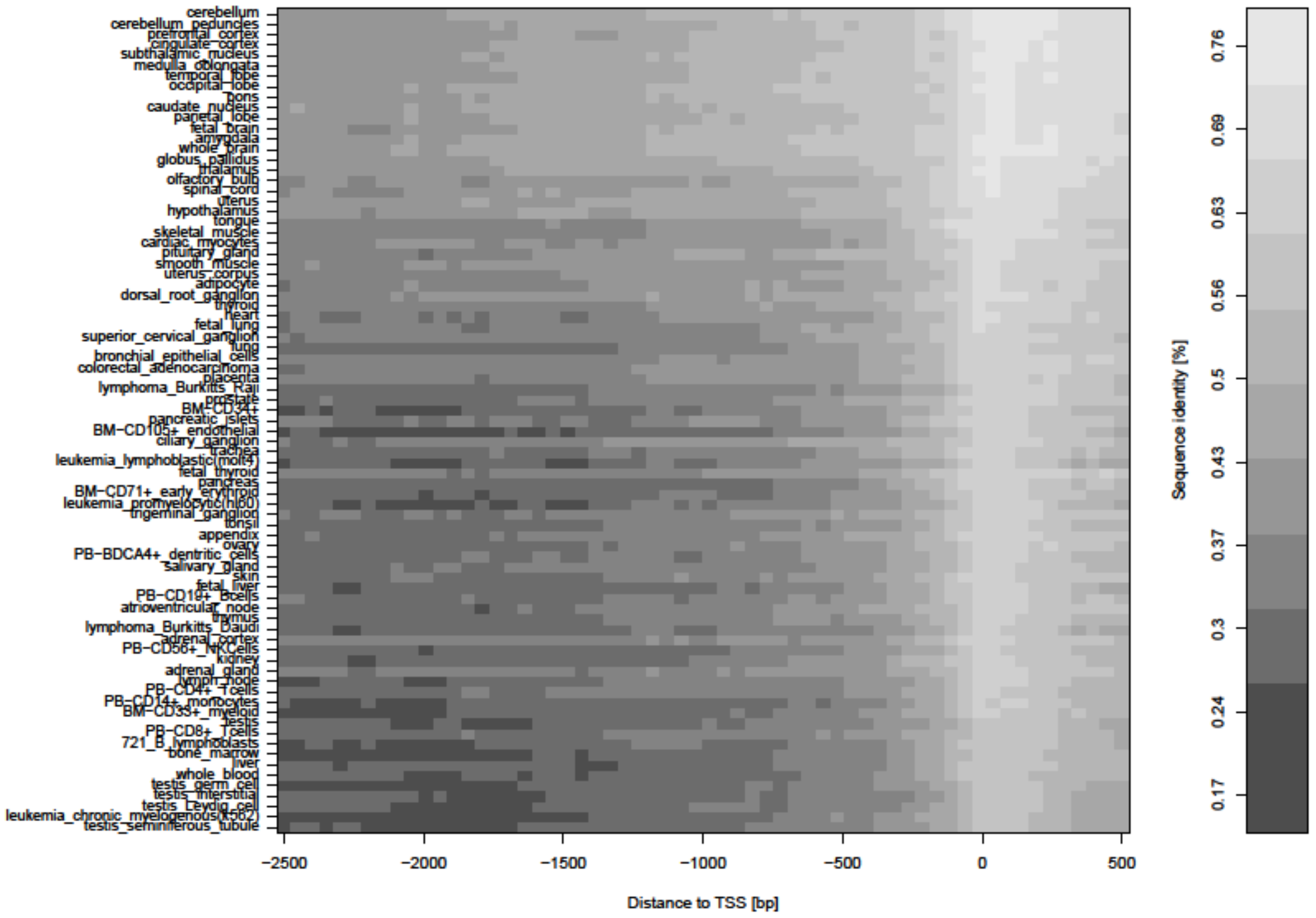

Figure VIII. percent of human identity between the human and mouse sequence of the promoter regions of 200 most highly expressed genes in 79 tissues. Sequence identity was calculated for bins of 50 bp, and is shown as a function of the distance to the TSS.

The promoters of highly expressed genes are enriched in TF binding sites

For most tissues, promoters of most highly expressed genes are significantly enriched in motifs as compared to the promoters of lowly expressed genes (p-values ≤ 0.05, Wilcoxon Rank-Sum test, corrected for multiple testing), containing binding sites for a median of 15 TFs (see Methods). Differences in the number of TFs with enriched binding sites may reflect biases in motif databases as well as reflect the size of TF families with members with similar binding sites. However, such differences are also likely to highlight contrasts in gene regulation across different tissues, both in terms of complexity, as well as differential usage of promoters and enhancers. Although CpG-rich promoters typically harbor relatively few overrepresented motifs [10], for promoters of most highly expressed genes we found that the number of TFs with significantly enriched binding sites does not strongly correlate with CpG content, and ranges between 94 for colorectal adenocarcinoma, to 0 in the case of BM-CD71+ early erythroid cells, leukemia promyelocytic (hl60), pituitary gland, testis germ cells, testis interstitial, and uterus corpus (Figure IX). Our inability to identify relevant TFs may suggest that, for some tissues, tissue-specific regulation mainly depends on enhancers or other factors such as chromatin structure.

Notably, the known biological role of the TFs with enriched binding sites matched their respective tissues. For instance, while 9 motifs enriched among promoters of genes expressed in fetal liver mainly correspond to binding sites for HNF1A [11], the 44 motifs enriched among promoters of genes expressed in the adult liver are consistent with the binding sites of a more extensive list of TFs, including HNF4A, PPARA, PPARG, NR5A2, and NR2F1, each known to play a major role in adult liver function [12-16]. Together, these observations are largely consistent with the established model in which ubiquitous regulatory activity is exerted by promoters [17-19], but also suggest that some promoters function in a tissue-specific manner, in agreement with recent evidence from next-generation sequencing projects [2].

We also detected differences in the distribution of enriched TF binding sites, which is not uniform along the promoter region. Enriched TF binding sites tend to co-localize towards the TSS (Figure X), but, in agreement with observations in yeast (e.g.,[20]), we observed differences between the architecture of TATA-containing and TATA-less promoters. In TATA-containing promoters, such as those of most genes expressed in the peripheral nervous system (trigeminal, dorsal root, and superior cervical ganglion), TF binding sites tend to be more broadly distributed further upstream of the TSS, possibly suggesting differences in the recruitment of pre-initiation complexes and implying different modes of regulation for transcription initiation among different groups of promoters, and, therefore, tissues.

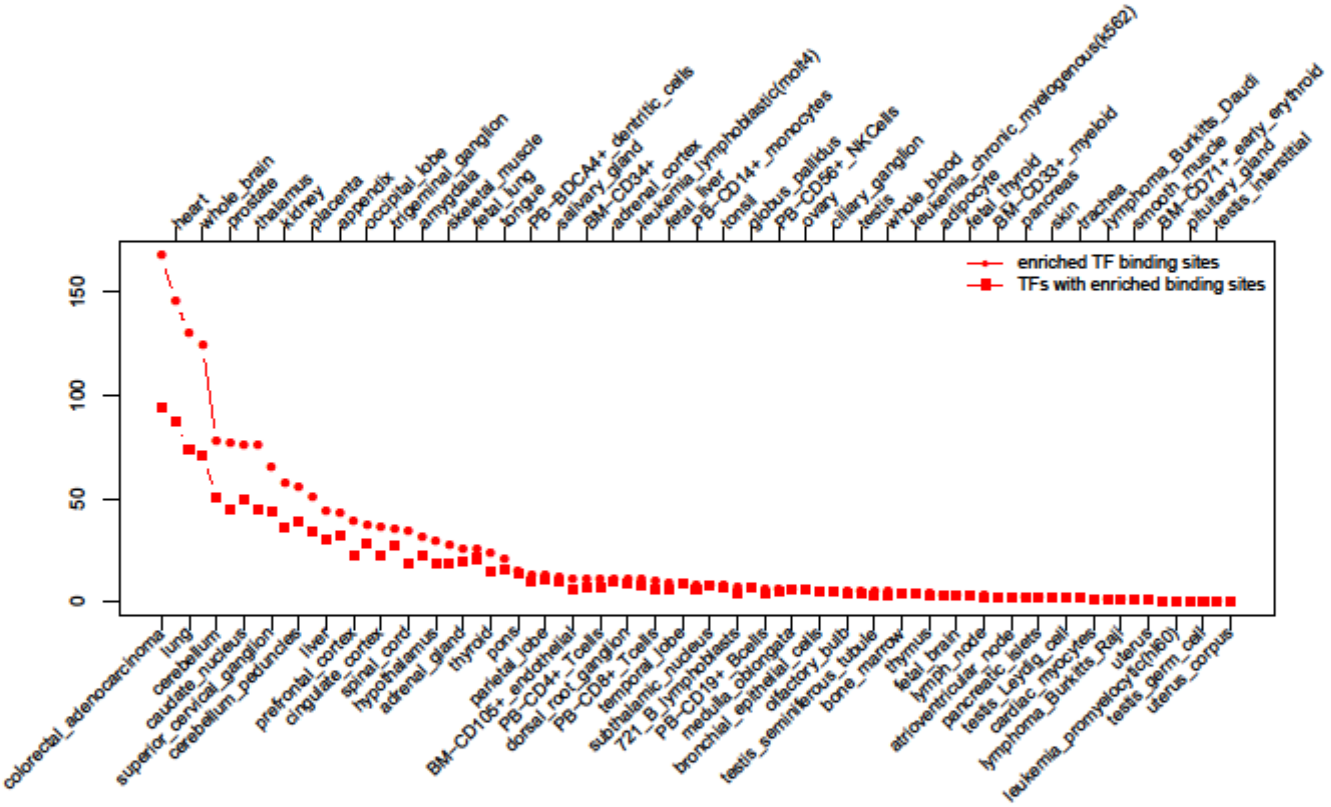

Figure IX. Enrichment of TF binding sites among the promoters of 200 most highly expressed genes in 79 tissues, compared to promoters of 200 most inhibited genes. Because of redundancy in TF binding site databases, most TFs are associated with multiple TF binding sites.

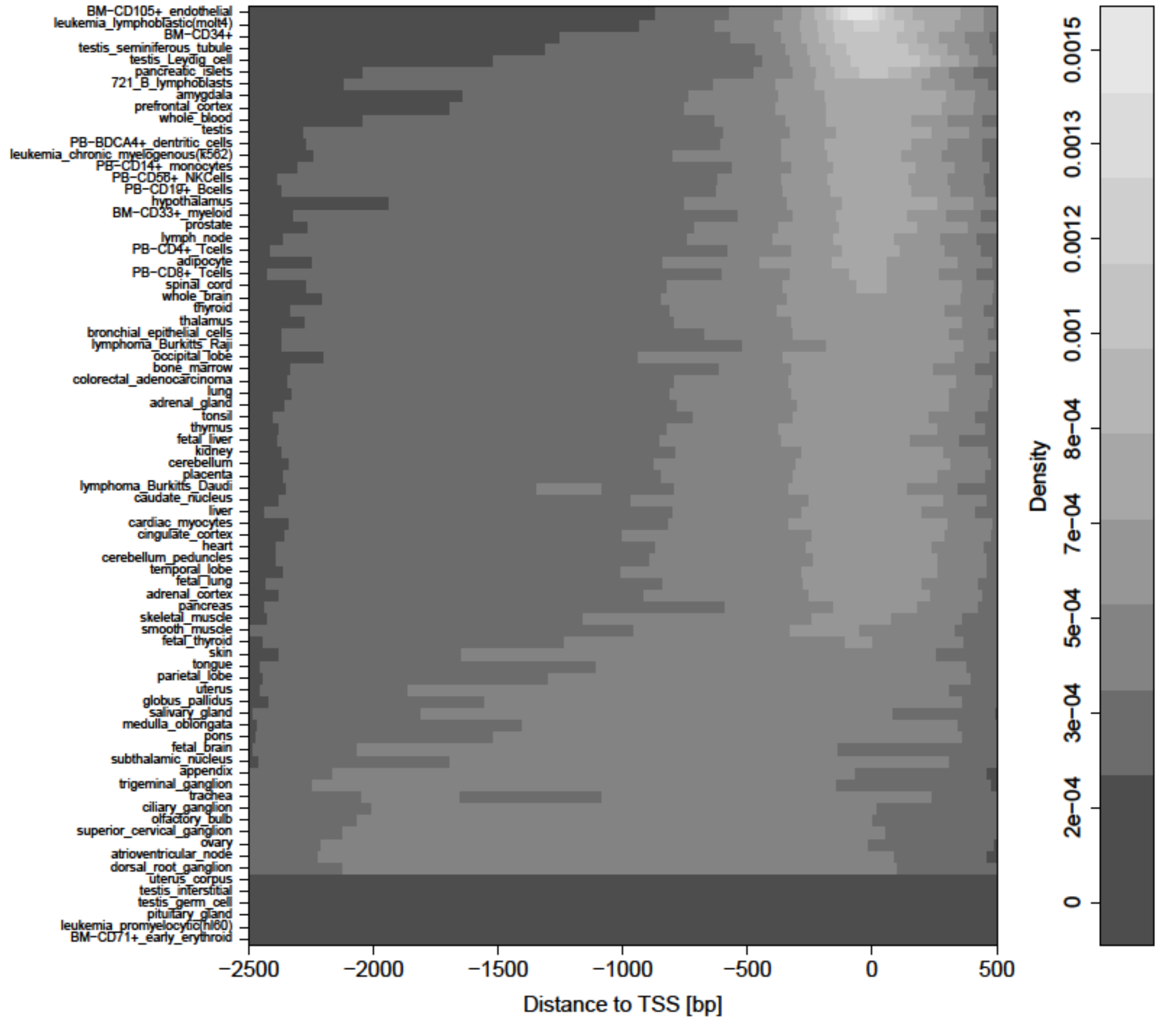

Figure X. Average density of significantly enriched TF binding sites per bp in promoters of the 200 most highly expressed genes in a given tissue as compared to the promoters of the 200 most inhibited genes in the same tissue, shown as a function of the distance to the TSS. Enriched TF binding sites tend to co-localize towards the TSS.

Differences in GC-content between promoters of highly expressed and inhibited genes contribute, but do not fully explain cross-validation performance of promoter-based models

Several hypotheses have been proposed to explain GC-content heterogeneity along the human genome, including a role for GC-content in chromatin organization and gene regulation [21, 22]. We did not observe a strong correlation between differences in the GC-content of the promoters associated with highly expressed and inhibited genes (p-value > 0.05, Spearman Rank Correlation Test). Moreover, the differences in GC-content tend to be smaller for reliable models (log2 fold-change = -0.02), as compared with those for unreliable models (log2 fold-change = -0.05). Furthermore, the GC-content of the most predictive TF binding sites does not necessarily reflect the difference in GC-content between the promoters of highly expressed and inhibited genes in a given tissue. For example, in the case of the liver model, which yielded an AUC value of 0.96 ± 0.04, promoters of highly expressed genes have significantly higher GC-content as compared to those of inhibited genes (0.53 and 0.50, respectively, p-value = 9.8×10^{-7}, Wilcoxon Rank-Sum Test), but there is no significant difference between the GC-content of the top and bottom 50 predictive TF binding sites (p-value > 0.5, Wilcoxon Rank-Sum Test). Also, we obtained an AUC value of

0.93 ± 0.05 after randomly permuting the nucleotide probabilities in the position-weight matrices (PWMs) which were used to identify TF binding sites (see Methods), which is significantly lower than the AUC value achieved by the original model (p-value = 0.01, Wilcoxon Rank-Sum Test). Taken together, these results demonstrate that differences in GC-content contribute but cannot fully explain the variation in performance of the models.

TF-based promoter-enhancer interactions

Our results suggest that TFs bound to both promoters and enhancers can at least partially explain the specificity observed in enhancer-promoter interactions. This mechanism may be of particular importance in complex genetic loci. To further investigate this relationship, we measured the number of putative enhancers associated with each promoter region. We found that for ~50% of tissues, the loci of genes with promoters exhibiting tissue-specific signatures harbor more enhancer predictions than those of genes with promoters with no tissue-specific signatures (p-values ≤ 0.05, Wilcoxon Rank-Sum test, Figure XI; the number of enhancer predictions was normalized to account for variations in locus length). For the remaining tissues we did not observe any significant differences. We hypothesize that while the expression of genes with promoters containing weak tissue-specific signatures might be regulated mainly by epigenetic modifications, genes relying heavily on promoter-enhancer interactions would incorporate an additional layer of control in the fine-tuning of tissue-specific transcription.

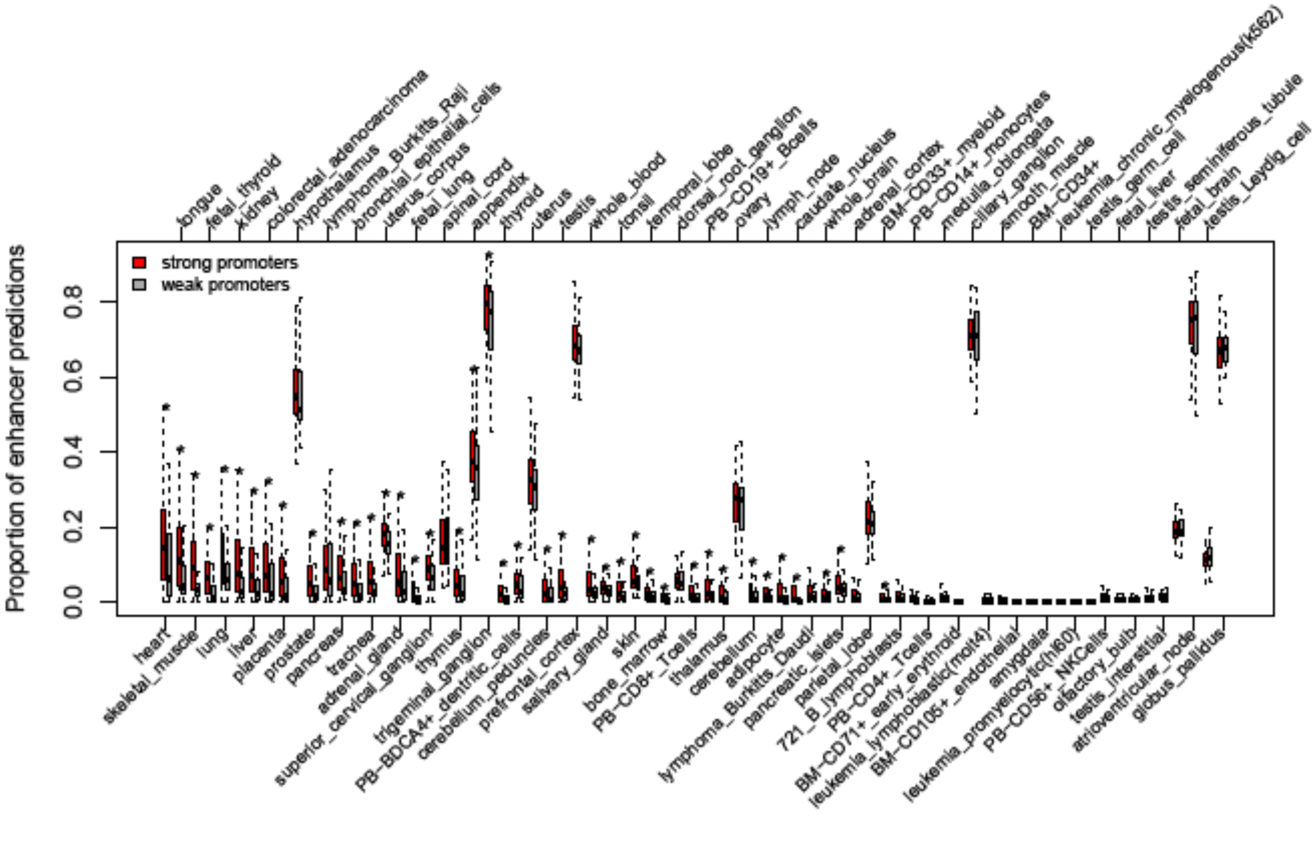

Figure XI. Proportion of enhancer predictions as a function of promoter strength. The proportion of enhancer prediction is computed as the number of enhancer predictions in loci of highly expressed genes divided by the total number of sequences scanned in loci of highly expressed genes. Promoter strength was assumed to be a function of the maximum score calculated for the CNEs in the promoter region with the corresponding tissue-specific model. While for 50% of the tissues considered loci of genes associated with stronger promoters tend to harbor more enhancers, for the remaining 50% loci of genes associated with stronger promoters tend to harbor less enhancers. The differences, however, are mainly significant for the former, which also display higher proportion of enhancer predictions. Significant differences in the

number of enhancer predictions relative to the total number of sequences scored in the loci of genes with strong promoters (red) as compared to weak promoters (gray) is indicated with an asterisk.

Conservation of promoter-based enhancer predictions

To determine the generality of this trend and detect enhancers that are under no detectable evolutionary constraint [23-25], we extended our functional analysis to sequences that are not necessarily conserved in mouse. To this end, we applied the promoter-based models to the sequence of loci of highly expressed and inhibited genes using a sliding window approach. For the size of the window, we chose the average length of conserved region between human and mouse [9], namely 230 bp. The sliding window was shifted by 115 bp. Windows overlapping the sequence 2.5 kb upstream and 0.5 kb downstream of the nearest TSS according to the refGene.txt and knownGene.txt tables (available in the UCSC Genome Browser database, [26]) were excluded from further analysis. A given sequence was considered an enhancer prediction if its score was greater than $s=min 0,\delta$, where δ is the lowest score of the top 5% sequences scored in the control loci.

As for predictions based on conserved noncoding sequences across the human and mouse genome, we evaluated the performance of the models by comparing the proportion of enhancer predictions and scanned sequences. For 70 % of the tissues our enhancer predictions are strongly enriched in loci of highly expressed genes as compared to inhibited genes (p-values $\leq$ 0.05, Fisher's Exact Test, Figure XII). The three healthy tissues showing the most pronounced enrichment are also heart, lung, and liver, with fold differences of 25, 9, and 6, respectively. Furthermore, our enhancer predictions show extensive overlap with noncoding sequences associated with enhancer activity, such as DHS sites (39 %) and H3K4me1 histone marks (55 %) in various ENCODE cell lines (with fold enrichments 1.3 and 1.2, respectively, p-values < 0.001, computed based on 1000 randomized sequences genome-wide), suggesting that the promoter-based models are also able to predict distal enhancers genome-wide, independent from sequence conservation. However, and although the level of conservation of these enhancer predictions varied depending on the tissue, we found that most predictions were well conserved in mammals (with 37 to 71% of predictions, depending on the tissue, overlapping CNEs, see Figure XIII). Consistent with previous results, we observed that whereas 11 % of predictions for enhancers active in the brain were conserved beyond mammals and birds, only 4 % of predictions for enhancers active in the skin were as deeply conserved. Indeed, p300-binding regions have been shown to exhibit a variable degree of evolutionary conservation across different tissues [25]. Also, for 80% of tissues, including brain, heart and liver, we observed that most conserved mammalian noncoding sequences [27] are significantly enriched in our predictions, as compared to the random expectation (p-value $\leq$ 0.05, calculated using the Wilcoxon Rank-Sum Test, Figure XIV), substantiating the focus on evolutionary conserved regions in genome-wide functional analysis.

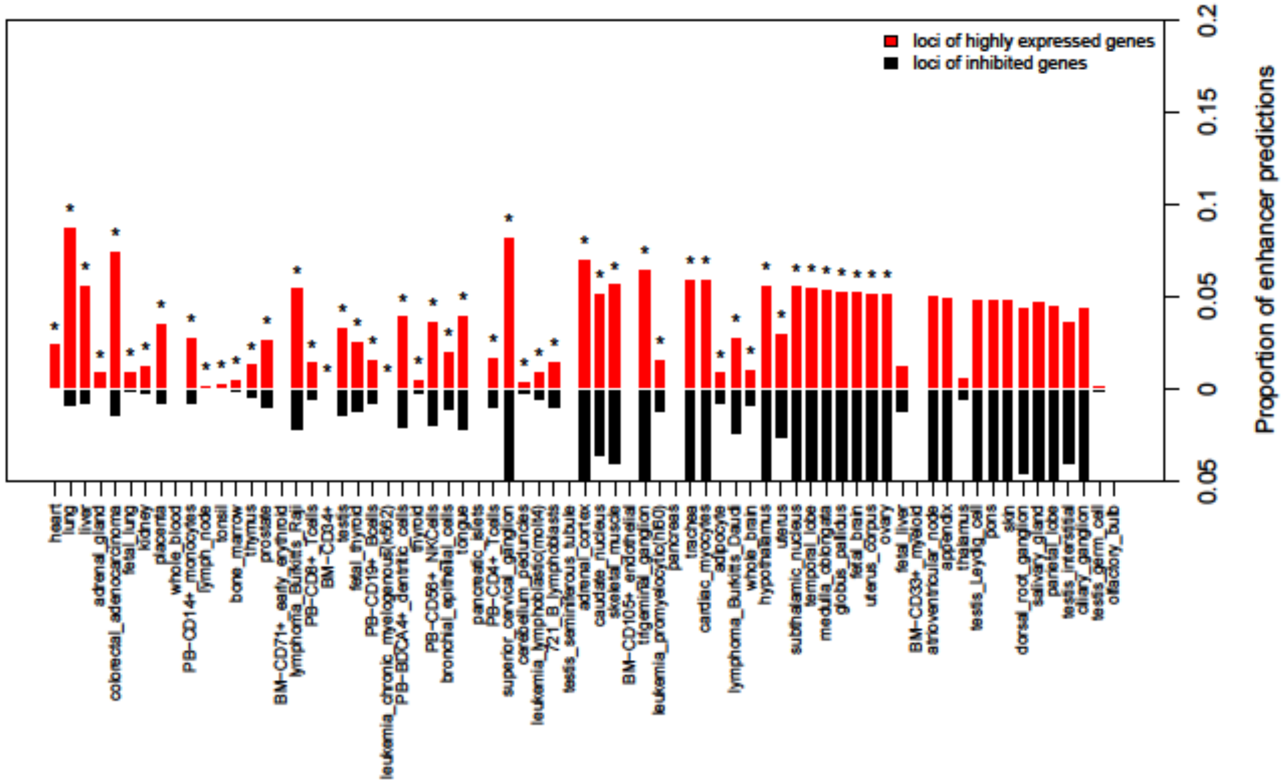

Figure XII. Proportion of tissue-specific enhancer predictions generated without sequence conservation constraints in loci of highly expressed and inhibited genes. Number of enhancer predictions in loci of highly expressed genes divided by the total number of sequences scanned in loci of highly expressed genes (in red), as compared to the number of enhancer predictions in loci of inhibited genes divided by the total number of sequences scanned in loci of inhibited genes (in black), for 71 promoter-based models. Statistically significant differences are indicated by asterisks (p-values ≤ 0.05, Fisher's Exact Test).

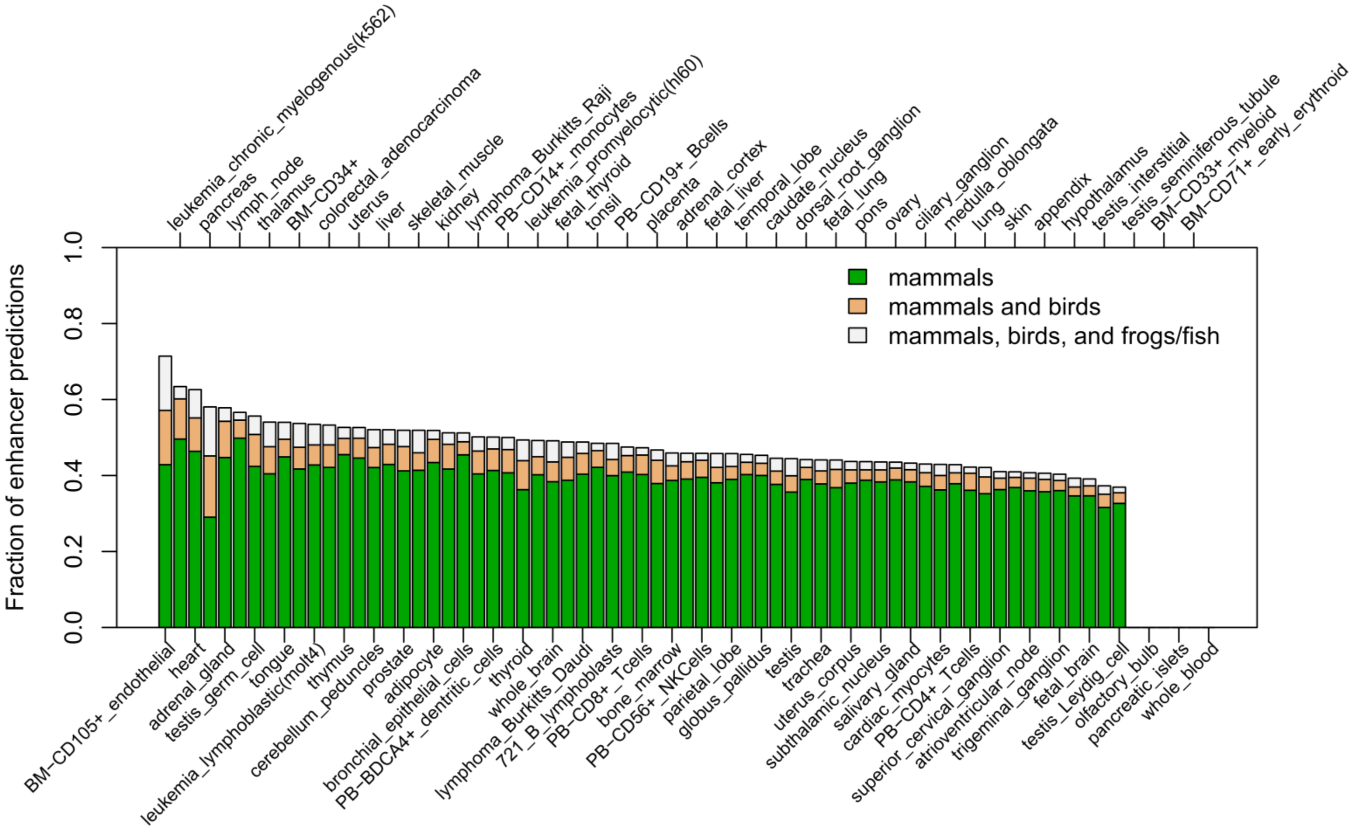

Figure XIII. Conservation level for tissue-specific enhancer predictions generated without sequence conservation constraints. Overlap of predictions in the human genome with conserved noncoding elements (CNEs) in mammals (mouse), birds (chicken), and frogs or fish for the tissues for which we obtained reliable promoter-based models.

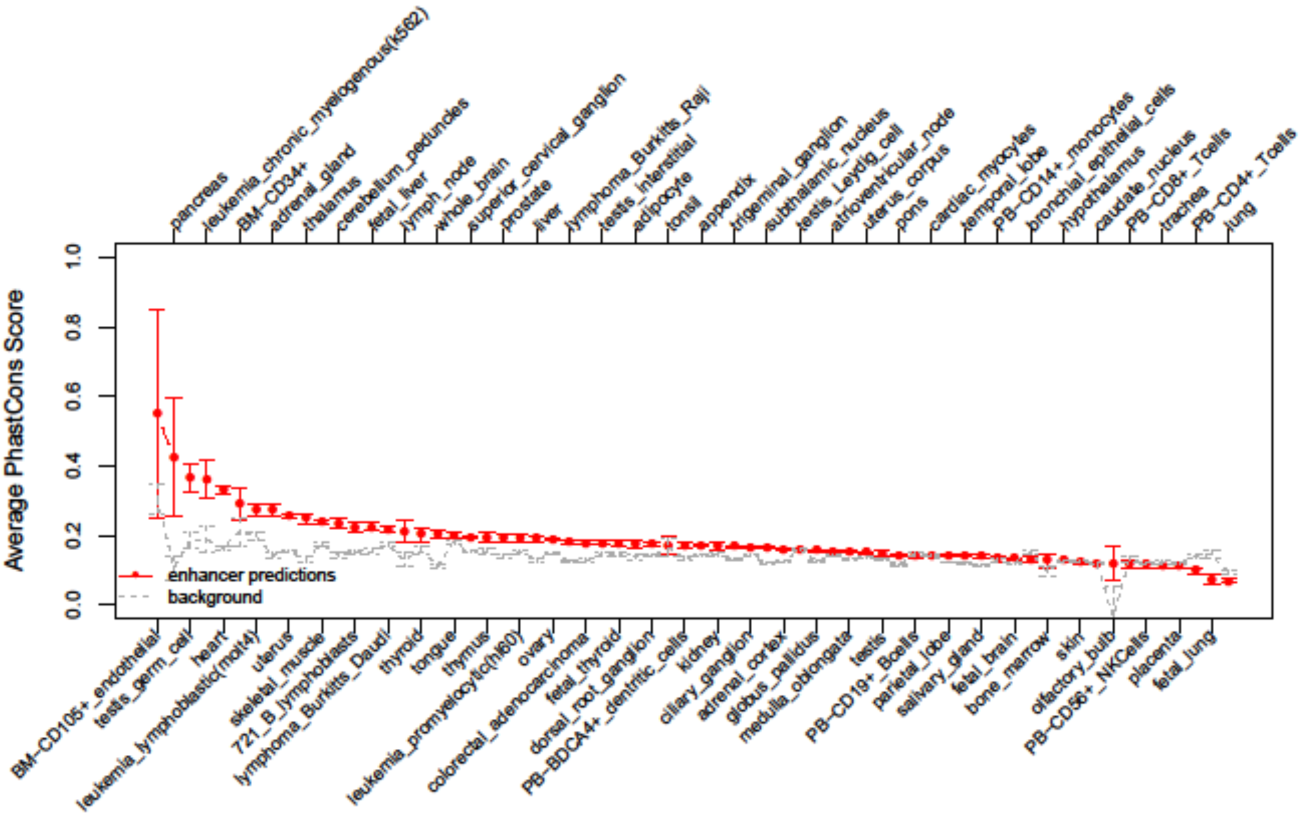

Figure XIV. Average PhastCons score for tissue-specific enhancer predictions generated without sequence conservation constraints (in red), compared to randomly selected sequences of similar length in loci of highly expressed genes.

References

1. MacIsaac KD, Lo KA, Gordon W, Motola S, Mazor T, Fraenkel E: **A quantitative model of transcriptional regulation reveals the influence of binding location on expression.** *PLoS Comput Biol* 2010, **6:**e1000773.
2. Shen Y, Yue F, McCleary DF, Ye Z, Edsall L, Kuan S, Wagner U, Dixon J, Lee L, Lobanenkov VV, Ren B: **A map of the cis-regulatory sequences in the mouse genome.** *Nature* 2012, **488:**116-120.
3. Ling G, Sugathan A, Mazor T, Fraenkel E, Waxman DJ: **Unbiased, genome-wide in vivo mapping of transcriptional regulatory elements reveals sex differences in chromatin structure associated with sex-specific liver gene expression.** *Mol Cell Biol* 2010, **30:**5531-5544.
4. Ernst J, Kheradpour P, Mikkelsen TS, Shoresh N, Ward LD, Epstein CB, Zhang X, Wang L, Issner R, Coyne M, et al: **Mapping and analysis of chromatin state dynamics in nine human cell types.** *Nature* 2011, **473:**43-49.
5. Hinrichs AS, Karolchik D, Baertsch R, Barber GP, Bejerano G, Clawson H, Diekhans M, Furey TS, Harte RA, Hsu F, et al: **The UCSC Genome Browser Database: update 2006.** *Nucleic Acids Res* 2006, **34:**D590-598.
6. The ENCODE Project Consortium: **The ENCODE (ENCyclopedia Of DNA Elements) Project.** *Science* 2004, **306:**636-640.
7. Su AI, Wiltshire T, Batalov S, Lapp H, Ching KA, Block D, Zhang J, Soden R, Hayakawa M, Kreiman G, et al: **A gene atlas of the mouse and human protein-encoding transcriptomes.** *Proc Natl Acad Sci U S A* 2004, **101:**6062-6067.
8. Farre D, Bellora N, Mularoni L, Messeguer X, Alba MM: **Housekeeping genes tend to show reduced upstream sequence conservation.** *Genome Biol* 2007, **8:**R140.
9. Loots G, Ovcharenko I: **ECRbase: database of evolutionary conserved regions, promoters, and transcription factor binding sites in vertebrate genomes.** *Bioinformatics* 2007, **23:**122-124.
10. Roider HG, Lenhard B, Kanhere A, Haas SA, Vingron M: **CpG-depleted promoters harbor tissue-specific transcription factor binding signals--implications for motif overrepresentation analyses.** *Nucleic Acids Res* 2009, **37:**6305-6315.
11. Courtois G, Morgan JG, Campbell LA, Fourel G, Crabtree GR: **Interaction of a liver-specific nuclear factor with the fibrinogen and alpha 1-antitrypsin promoters.** *Science* 1987, **238:**688-692.
12. Sladek FM, Zhong WM, Lai E, Darnell JE, Jr.: **Liver-enriched transcription factor HNF-4 is a novel member of the steroid hormone receptor superfamily.** *Genes Dev* 1990, **4:**2353-2365.
13. Aoyama T, Peters JM, Iritani N, Nakajima T, Furihata K, Hashimoto T, Gonzalez FJ: **Altered constitutive expression of fatty acid-metabolizing enzymes in mice lacking the peroxisome proliferator-activated receptor alpha (PPARalpha).** *J Biol Chem* 1998, **273:**5678-5684.
14. Gavrilova O, Haluzik M, Matsusue K, Cutson JJ, Johnson L, Dietz KR, Nicol CJ, Vinson C, Gonzalez FJ, Reitman ML: **Liver peroxisome proliferator-activated receptor gamma contributes to hepatic steatosis, triglyceride clearance, and regulation of body fat mass.** *J Biol Chem* 2003, **278:**34268-34276.
15. Fayard E, Auwerx J, Schoonjans K: **LRH-1: an orphan nuclear receptor involved in development, metabolism and steroidogenesis.** *Trends Cell Biol* 2004, **14:**250-260.
16. Zhang P, Bennoun M, Gogard C, Bossard P, Leclerc I, Kahn A, Vasseur-Cognet M: **Expression of COUP-TFII in metabolic tissues during development.** *Mech Dev* 2002, **119:**109-114.
17. Arnone MI, Davidson EH: **The hardwiring of development: organization and function of genomic regulatory systems.** *Development* 1997, **124:**1851-1864.
18. Pennacchio LA, Loots GG, Nobrega MA, Ovcharenko I: **Predicting tissue-specific enhancers in the human genome.** *Genome Res* 2007, **17:**201-211.
19. Michelson AM: **Deciphering genetic regulatory codes: a challenge for functional genomics.** *Proc Natl Acad Sci U S A* 2002, **99:**546-548.
20. Erb I, van Nimwegen E: **Transcription factor binding site positioning in yeast: proximal promoter motifs characterize TATA-less promoters.** *PLoS One* 2011, **6:**e24279.
21. Romiguier J, Ranwez V, Douzery EJ, Galtier N: **Contrasting GC-content dynamics across 33 mammalian genomes: relationship with life-history traits and chromosome sizes.** *Genome Res* 2010, **20:**1001-1009.

22. Vinogradov AE: **Dualism of gene GC content and CpG pattern in regard to expression in the human genome: magnitude versus breadth.** *Trends Genet* 2005, **21:**639-643.
23. Schmidt D, Wilson M, Ballester B, Schwalie P, Brown G, Marshall A, Kutter C, Watt S, Martinez-Jimenez C, Mackay S, et al: **Five-Vertebrate ChIP-seq Reveals the Evolutionary Dynamics of Transcription Factor Binding.** *Science* 2010, **328:**1036-1040.
24. Fisher S, Grice EA, Vinton RM, Bessling SL, McCallion AS: **Conservation of RET Regulatory Function from Human to Zebrafish Without Sequence Similarity.** *Science* 2006**:**1124070.
25. Blow MJ, McCulley DJ, Li Z, Zhang T, Akiyama JA, Holt A, Plajzer-Frick I, Shoukry M, Wright C, Chen F, et al: **ChIP-Seq identification of weakly conserved heart enhancers.** *Nat Genet* 2010, **42:**806-810.
26. Kent WJ, Sugnet CW, Furey TS, Roskin KM, Pringle TH, Zahler AM, Haussler D: **The human genome browser at UCSC.** *Genome Res* 2002, **12:**996-1006.
27. Siepel A, Bejerano G, Pedersen JS, Hinrichs AS, Hou M, Rosenbloom K, Clawson H, Spieth J, Hillier LW, Richards S, et al: **Evolutionarily conserved elements in vertebrate, insect, worm, and yeast genomes.** *Genome Res* 2005, **15:**1034-1050.
